# Favorable pleiotropic loci for fiber yield and quality in upland cotton (*Gossypium hirsutum*)

**DOI:** 10.1038/s41598-021-95629-9

**Published:** 2021-08-05

**Authors:** Pengpeng Wang, Shoupu He, Gaofei Sun, Zhaoe Pan, Junling Sun, Xiaoli Geng, Zhen Peng, Wenfang Gong, Liru Wang, Baoyin Pang, Yinhua Jia, Xiongming Du

**Affiliations:** 1 Institute of Cotton Research, Chinese Academy of Agricultural Sciences/Zhengzhou Research Base, State Key Laboratory of Cotton Biology, Zhengzhou University, Zhengzhou, Henan 450000 People’s Republic of China; 2 School of Computer Science and Information Engineering, Anyang Institute of Technology, Anyang, Henan 455000 People’s Republic of China

**Keywords:** Computational biology and bioinformatics, Plant sciences

## Abstract

Upland cotton (*Gossypium hirsutum *L.) is an important economic crop for renewable textile fibers. However, the simultaneous improvement of yield and fiber quality in cotton is difficult as the linkage drag. Compared with breaking the linkage drag, identification of the favorable pleiotropic loci on the genome level by genome-wide association study (GWAS) provides a new way to improve the yield and fiber quality simultaneously. In our study restriction-site-associated DNA sequencing (RAD-seq) was used to genotype 316 cotton accessions. Eight major traits in three categories including yield, fiber quality and maturation were investigated in nine environments (3 sites × 3 years). 231 SNPs associated with these eight traits (− log_10_(*P*) > 5.27) were identified, located in 27 genomic regions respectively by linkage disequilibrium analysis. Further analysis showed that four genomic regions (the region 1, 6, 8 and 23) held favorable pleiotropic loci and 6 candidate genes were identified. Through genotyping, 14 elite accessions carrying the favorable loci on four pleiotropic regions were identified. These favorable pleiotropic loci and elite genotypes identified in this study will be utilized to improve the yield and fiber quality simultaneously in future cotton breeding.

## Introduction

Upland cotton (*Gossypium hirsutum* L.) is one of the most important economic crops, contributing 90% of global cotton fiber production, and is considered the main source of renewable textile fibers^1^. The cotton industry is an important component of the world economy^2^. Yield and fiber quality, which are closely linked with cotton fiber production, are the most important traits of cotton^3–6^. In early cotton breeding, yield and fiber quality were negatively associated due to the introduction of Beasley’s triple hybrid^7^. Pleiotropic loci or linkage drag were the main reasons for this negative correlation^8,9^.

Pleiotropy occurs when one gene contributes to multiple phenotypic traits^10,11^. A gene that influences two or more phenotypic expressions is called a pleiotropic gene^12^. Pleiotropy can occur if the gene or its targets function at different development stages or in different signal pathways^13^. Pleiotropy can arise from several distinct but potentially overlapping mechanisms, such as gene pleiotropy and region pleiotropy^14^. Region pleiotropy occurs when a region is linked with and influences two or more traits. Pleiotropic regions can be divided into two categories by their functions; one is unwanted linkage and is called linkage drag, the other is favorable linkage^14^.

Linkage drag is often observed in crop breeding^15–18^. When a gene of interest is identified, it can be transferred from one species to another using interspecific hybridization followed by backcrossing or intergeneric gene transfer^16^. However, unfavorable associations between the gene of interest and other characteristics may be found; for example, the introduction of a tobacco mosaic virus (TMV) resistance gene resulted in a yield decrease in tobacco because of pleiotropy of the gene and/or linkage drag^19^. This phenomenon has also been found in other crops. In rice, linkage drag between disease-resistance and yield was detected on chromosome 6^20^. Linkage drag between root development and heading date was found in wheat^21^. Additionally, in tomato, genes underlying fruit chemistry can affect metabolic quality, as a result of linkage drag. Linkage drag has limited the improvement of crops via conventional breeding. For example, in tomato, linkage drags affect nearly 200 Mb genome regions (25.6% of the assembled genome)^22^.

Linkage drag is equally serious in cotton breeding^23^. Among cotton species, *Gossypium barbadense* has excellent fiber quality, while the yield of *G. hirsutum* is high^24–26^. Linkage drag not only makes it difficult to transfer favorable traits from *G. barbadense* to *G. hirsutum*^4^, but also significantly limits the simultaneous improvement of yield and fiber quality in *G. hirsutum*^8,9^. With the development of crop breeding, the linkage drag of yield and fiber quality was disrupted by the work of Culp et al.^27^. Although the relationship between fiber quality and yield has changed from linkage drag to favorable linkage, the molecular mechanism underlying this change has never been reported.

The identification of favorable pleiotropic loci for fiber yield and quality is very important for cotton breeding. With the development of sequencing technologies, single nucleotide polymorphism (SNP)-based genome-wide association study (GWAS) has become an effective method to identify favorable pleiotropic loci across the whole genome in plant^28^. Many regions or genes associated with multiple traits have been identified by GWAS. For example, two pleiotropic regions were identified by GWAS in rice. One region was located on chromosome 11 and was associated with plant height and panicle length; the other region was associated with panicle number, spikelet number and leaf blade width, and was located on chromosome 5^29,30^. In soybean, the *Ln* gene for four-seed pods and leaf shape was identified by GWAS^31^. In cotton, four QTL regions associated with multiple traits affecting yield and fiber quality were identified using the CottonSNP63K array^32^. It was also reported that 4820 genes were associated with different traits simultaneously in upland cotton by resequencing and GWAS^33^. Some regions or genes associated with more than one yield trait have been identified, but they nearly all had no effect on fiber quality^4^. Few loci both benefited to fiber quality and yield have been reported.

Our work aimed to identify favorable pleiotropic loci associated with fiber quality and yield, and accessions carrying these pleiotropic loci. In our experiment, RAD-seq and GWAS were combined to identify favorable pleiotropic loci and related genes in cotton. Phenotypic data were obtained from three locations over three years (3 × 3), which help us to explain the relationship between fiber quality and yield. Four regions (regions 1, 6, 8 and 23) and 14 elite accessions were picked as carrying favorable pleiotropic loci, could be utilized in future cotton breeding directly.

## Results

### Correlations between yield and fiber quality in upland cotton

Data for the eight traits under nine environments, including yield components-, fiber quality- and maturity-related traits of upland cotton are list (Additional file 1: Table S1). After obtaining the trait data, we did descriptive statistics analysis, ANOVA analysis and correlation analysis. Through descriptive statistics analysis, the Coefficient of variation (CV) of fiber yield was bigger than fiber quality (Additional file 1: Table S2), indicting the dispersion degree of fiber quality was lower than fiber yield. By two-way ANOVA analysis (Additional file 1: Table S3 and Additional file 2: Fig. S1a), a high proportion of genotype effect was identified in most of the trait, like boll weight (BW), seed index (SI), lint percentage (LP), fiber length (FL) and fiber strength (FS). But for flowering data (FD) trait, the environment effect took up a great proportion. In addition, there was a significant genotype x environment interaction (G × E) for all the traits.

The pairwise Pearson’s correlation coefficients between phenotypes are calculated, the Best Linear Unbiased Prediction (BLUP) result of each trait under nine environments were used as input data (Additional file 2: Fig. S1b). For the yield components, a highly significant positive correlation (0.25) was observed between BW and SI, while LP had a negative correlation (− 0.21) with SI. As important indicators of fiber quality, FS had a strong positive correlation (0.78) with FL. Negative relationships were found between FL and fiber micronaire (MIC) (− 0.29), FL and fiber elongation (FE) (− 0.47), FS and MIC (− 0.25), and FS and FE (− 0.56).

We also found a complicated relationship between the yield and fiber quality traits (Additional file 2: Fig. S1b). Negative correlations were found between BW and FE (− 0.14), SI and MIC (− 0.2), and LP and FS (− 0.14). Significant positive correlations were found between BW and FL (0.30), BW and FS (0.37), and LP and MIC (0.35).

Positive correlations were found between FD and BW (0.24), SI (0.15), FL (0.18), and FS (0.28), while negative correlations were found between FD and LP (− 0.18) and FE (− 0.34), indicating a later FD contributes to better yield and fiber quality. The relationship of fiber yield, quality and FD may facilitate the GWAS identification of pleiotropic regions and genes.

The next correlation analyses of each traits under the nine environments showed that some traits were stable under different environment including FL, FS, BW, SI and LP, while some traits were not, including FE and FD (Additional file 2: Figs. S2 and S3). These data indicated there are complex relationships among yield, quality and FD, with positive correlations, negative correlations and non-correlation.

### SNP identification and population structure analysis

**Figure 1:**
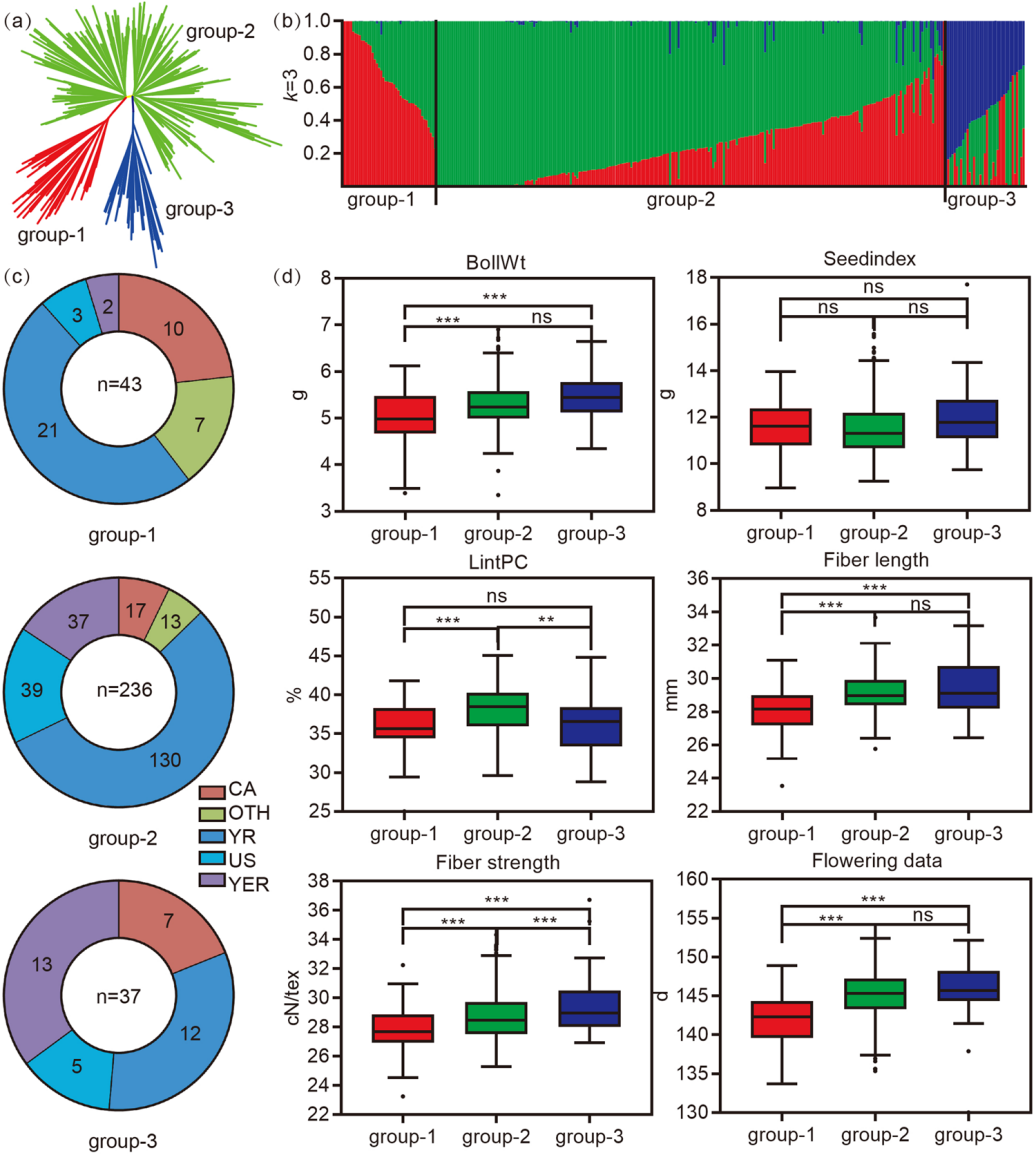
Phylogenetic relationships of 316 cotton accessions: (**a**) A neighbor-joining tree was constructed using whole-genome SNP data. The accessions were divided into three groups, group-1 (red), group-2 (cyan) and group-3 (blue); (**b**) Population structure of cotton accessions. The cotton samples were divided into three groups when k = 3; (**c**) Geographic origin of the three groups, Central Asia (CA), the United States (US), the Yellow River (YR), the Yangtze River (YZR) and other places (OTH); (**d**) Phenotype distributions of yield and fiber quality traits, the group divided by the structure of the 316 accessions, Boll weight (BW), Seed index (SI), Lint PC (LP), Fiber length (FL), Fiber strength (FS) and Flowering data (FD). Within boxplots, the bold line represents the median, box edges represent upper and lower quantiles, and whiskers are 1.5 times the quantile of the data. Outliers are shown as open dots (*P < 0.05, **P < 0.01 and ***P < 0.001, two-sided t-test). The neighbor-joining tree (**a**) was constructed using the software PHYLIP (v3.696, https://evolution.genetics.washington.edu/phylip.html). Population structure of cotton accessions (**b**) was determined using the software Admixture (v1.30, http://dalexander.github.io/admixture/index.html). The others were created by the software GraphPad Prism 9 (ver. 9.0.0, http://www.graphpad.com).

The read numbers and paired read mapping ratios are shown in Table S2. In total, 93,687 high-quality SNPs were detected in our study, 42,564 of which (MAF > 0.05, missing < 0.1, heterogeneity < 0.3) were used to construct a phylogenetic tree (Fig. 1a) and determine the population structure (Fig. 1b). According to the phylogenetic tree and population structure, the 316 upland cotton accessions were divided into three groups, with 43 accessions in group-1, 236 accessions in group-2 and 37 accessions in group-3 (Fig. 1c, Additional file 1: Tables S4 and S5). Group-3 had the most abundant genetic diversity, compared with group-1 and -2 (Fig. 1b). According to their ecological characteristics, the 316 upland cotton accessions were divided into five groups (Central Asia region, CA, Yellow River region, YR, United States, US, Yangtze River region, YZR and other regions, OTH) (Fig. 1c and Additional file 1: Table S4). In group-1, the accessions from YR represented the largest proportion, while the accessions from YR in the group-2 and the accessions from YER in the group-3 represented the largest proportion (Fig. 1c), respectively.

For the yield and fiber quality in the three groups, group-3 with greater percentage of YZR accessions had higher BW and SI, better FL and FS than that in group-1 and group-2 (Fig. 1c,d). Group-1, with bigger percentage of accessions from CA than group-2 and group-3, had a shorter FD (Fig. 1c,d). Thus, the accessions from YZR had better yield and fiber quality, while those from CA had a shorter FD.

### Identification of pleiotropic regions by GWAS

We performed genome-wide association analysis based on 93,687 high-quality SNPs and eight traits using the single-locus GWAS methods (EMMAX)^34^ and multi-locus GWAS methods (mrMLM). Trait data from different environments were calculated independently to compare environmental effects and screen common loci with strong signals. In total, 231 key SNPs (− log_10_(*P*) > 5.27) were identified, and 54 common SNPs can also be identified by multi-locus GWAS (Additional file 1: Tables S6 and S7). Among them, 223 SNPs were located in intergenic regions and the Chromosome A07 contained the most associated SNPs (161) among all chromosomes. Among the traits, SI, FS and BW had 80, 69 and 33 stronger associated SNPs, respectively (Additional file 1: Tables S8 and S9). 37 SNPs were detected multiple for different traits or different environments (Additional file 1: Table S6). For example, the SNP A07_72184095 was commonly detected for SI, FE and FS (Additional file 1: Table S6). A07_72184095 was also the SNP most significantly related to both yield components and fiber quality (− log_10_(*P*) = 8.56).

Based on the LD block analysis, all key SNPs were categorized into 27 regions, including 200 genes (Additional file 1: Table S10). Among the 27 regions, 12 regions were only associated with yield traits, 11 regions were only associated with fiber quality, two regions were only associated with flowering date, and four regions were pleiotropic regions with three regions associated to fiber quality and yield, and one region associated with yield and flowering date (Additional file 1: Tables S10 and S11).

### Four pleiotropic regions associated with fiber yield, fiber quality and flowering date

In our experiment, four regions (LD block 1, 6, 8 and 23) were identified as pleiotropic regions, located on chromosomes A06, A07 and D11 (Additional file 1: Table S11). LD blocks 6, 8 and 23 were commonly associated with yield and fiber quality, while LD block 1 on A06 chromosome was associated with yield and flowering date. Among the four pleiotropic regions, LD block 8 and 23 could be verified by the multi-locus GWAS, 38 and 12 SNPs involved respectively (Additional file 1: Table S7).

**Figure 2:**
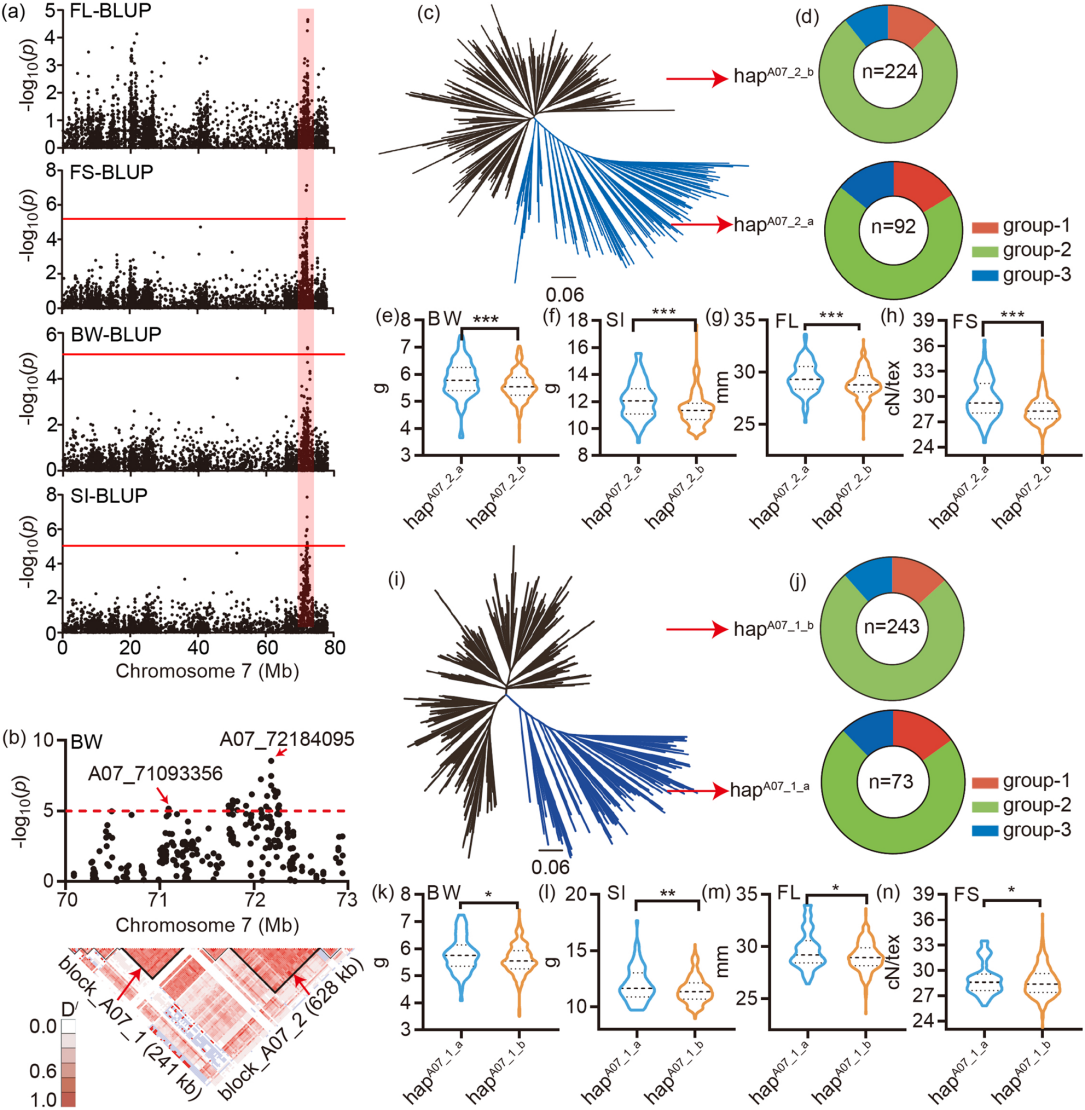
Identification and genotyping of the pleotropic region LD block 6 and 8 on chromosome A07: (**a**) The Manhattan block of the FL, SI, FS and BW, the red horizontal dashed lines indicate the significance threshold (− log_10_(*P*) > 5.27); the red box indicates the region identified by the GWAS of FL-blup, FS-blup, BW-blup and SI-blup, located between 70 to 73 Mb on A07 chromosome; (**b**) Local Manhattan block (top) and LD heat map (bottom). The red arrows indicate the most significance SNPs in the two LD blocks at the top and the location of the two LD blocks (block_A07_1 and block_A07_2) at the bottom; (**c**) A neighbor-joining tree was constructed using the SNP data located in the LD block 12. The accessions were divided into two haplotypes, hap^A07_2_a^ and hap^A07_2_b^; (**d**) The composition of the two haplotypes, group-1 (red), group-2 (cyan) and group-3 (blue); (**e**–**h**) Box plots for boll weight, length, strength and seed index for the two haplotypes (n = 92 and 224); (**i**) A neighbor-joining tree was constructed using the SNP data located in the LD block 10. The accessions were divided into two haplotypes, hap^A07_1_a^ and hap^A07_1_b^; (**j**) The composition of the two haplotypes, group-1 (red), group-2 (cyan) and group-3 (blue); (**k**–**n**) Box plots for boll weight, length, strength and seed index for the two haplotypes (n = 243 and 73). Center line, median; box limits, upper and lower quartiles. whiskers, 1.5× the interquartile range; dots, outliers (*P < 0.05, **P < 0.01 and ***P < 0.001, two-sided t-test). The neighbor-joining tree (**c** and **i**) was constructed using the software PHYLIP (v3.696, https://evolution.genetics.washington.edu/phylip.html). The LD heatmap (the bottom of **b**) was created by the Haploview software (https://www.broadinstitute.org/haploview/haploview). The others were created by the software GraphPad Prism 9 (ver. 9.0.0, http://www.graphpad.com).

Among the four pleiotropic regions, LD block 6 and LD block 8 were both located on the chromosome A07 (Fig. 2a,b) and could not be distinguished by the Manhattan plot (Fig. 2a). Importantly, the two regions could be identified by the BLUP was used for GWAS analysis of the trait BW, SI and FS. For the trait FL, SNP peak could be identified, while − log_10_(*P*) = 4.65 (Figs. 2a and S4). The two regions were associated with BW at four environments (Additional file 2: Fig. S5), FL at three environments (Additional file 2: Fig. S6), FS at five environments (Additional file 2: Fig. S7) and SI at nine environments (Additional file 2: Figs. S8 and S9).

The LD block 8 between 71.73 and 72.36 Mb genome region on chromosome A07, was the most significant pleiotropic region and associated with four traits, BW, SI, FL and FS (Fig. 2a,b). The phylogenetic tree was construct by the SNP involved in the block 12 (Fig. 2c), the result showed that 316 accessions could be divided into two group, hap^A07_2_a^ and hap^A07_2_b^. Through genotyping, the group hap^A07_2_a^ had higher fiber yield (BW and SI) and better fiber quality (FL and FS) than the group hap^A07_2_b^ (Fig. 2e–h). And the number of accessions carrying the hap^A07_2_a^ was only 92, 29 percent of the total accessions). This indicated the hap^A07_2_a^ was favorable rare haploid type for better fiber quality and yield. In total, 18 genes were located in the LD block 12 region (Additional file 1: Table S10). Among them, 3 genes had different expression pattern in our transcript data (Additional file 2: Fig. S10, Additional file 1: Tables S10 and S13). *Gh_A07G1767* was highly expressed at 5 and 10 DPA fiber; *Gh_A07G1771* was highly expressed at 5 DPA fiber (Additional file 2: Fig. S10a,b). These two genes maybe regulate to fiber length. *Gh_A07G1774* had a high expression level at 3 DPA in the better fiber quality line J02-508, nearly threefold change than that in the poor-quality line ZRI015 (Additional file 2: Fig. S10c).

LD block 6 was located between 71.0 and 71.3 Mb on chromosome A07 (Fig. 2a,b), associated with four traits, FL, BW, SI and FS. 316 accessions were clustered two groups, hap^A07_1_a^ and hap^A07_1_b^ by the phylogenetic tree (Fig. 2i). The genotyping showed that the group hap^A07_1_a^ had higher yield (BW and SI) and better fiber quality (FL and FS) (Fig. 2k–n). The group hap^A07_1_a^ had less accessions (n = 73), compared with the group hap^A07_1_b^ (Fig. 2j), indicting the elite haplotype hap^A07_1_a^ have not widely been used in cotton breeding. 17 genes were located in this region (Additional file 1: Table S10). Among the 17 genes in the LD block 10 (Additional file 2: Fig. S10c, Additional file 1: Tables S10 and S13), *Gh_A07G1731* had a higher expression level at 5, 10 and 15 DPA, nearly fourfold change at 15 DPA in J02-508 than ZRI015, indicating a role in the regulation of fiber quality (Additional file 2: Fig. S10a).

**Figure 3:**
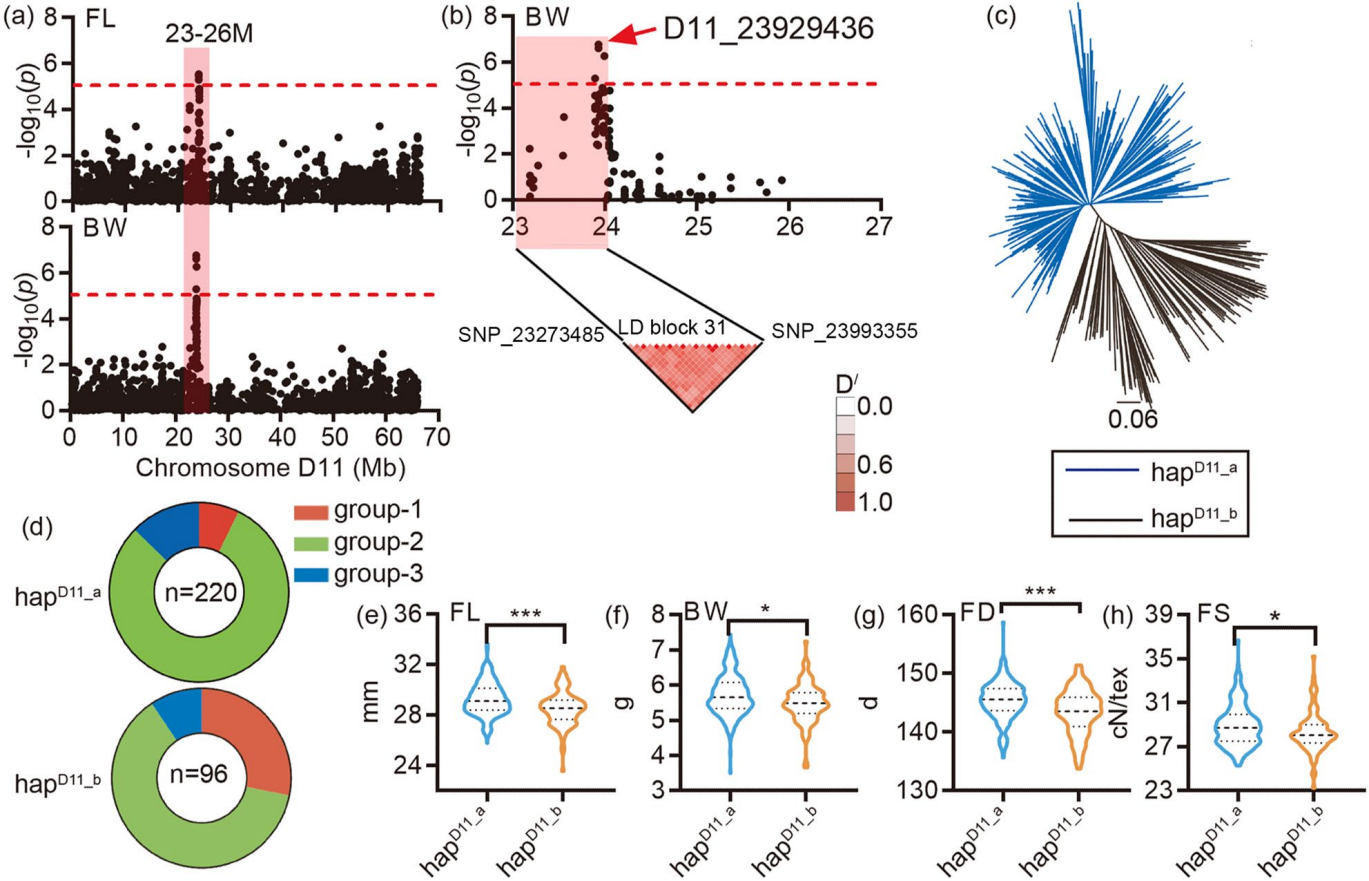
Identification and genotyping of the pleotropic region LD block 23 on chromosome D11: (**a**) The Manhattan block of the FL and BW, the red horizontal dashed lines indicate the significance threshold (− log_10_(*P*) > 5.27). The red box indicates the region identified by the GWAS of FL and BW, located between 23 to 26 Mb on D11 chromosome; (**b**) Local Manhattan block (top) and LD heat map (bottom). The red arrows indicate the most significance SNP in the LD block at the top and the location of the block (LD block 31) at the bottom; (**c**) A neighbor-joining tree was constructed using the SNP data located in the LD block 31. The accessions were divided into two groups, hap^D11_a^ and hap^D11_b^; (**d**) The composition of the two haplotypes, group-1 (red), group-2 (cyan) and group-3 (blue); (**e**–**h**) Box plots for FL, BW, FD and FS for the two haplotypes (n = 220 and 96). Center line, median; box limits, upper and lower quartiles; whiskers, 1.5× the interquartile range; dots, outliers (*P < 0.05, **P < 0.01 and ***P < 0.001, two-sided t-test). The neighbor-joining tree (**c**) was constructed using the software PHYLIP (v3.696, https://evolution.genetics.washington.edu/phylip.html). The LD heatmap (the bottom of **b**) was created by the Haploview software (https://www.broadinstitute.org/haploview/haploview). The others were created by the software GraphPad Prism 9 (ver. 9.0.0, http://www.graphpad.com).

The next pleiotropic region associated with yield and fiber quality was LD block 23, located on chromosome D11, associated with BW and FL (Fig. 3a), which only could be detected at one same environment Kuche_2007 (Additional file 2: Fig. S11b). The most significance of the SNP in this region was D11_23929436 (Fig. 3b), was located in the LD block 31 (Table S10). 316 accessions were clustered two groups, hap^D11_a^ and hap^D11_b^ by the phylogenetic tree (Fig. 3c). The group hap^D11_a^ had more accessions (n = 220) than the group hap^D11_b^ (Fig. 3d), indicting the elite haplotype hap^D11_a^ have widely been used in cotton breeding. The genotyping results indicated that the group hap^D11_a^ had higher yield (BW and SI) and better fiber quality (FL and FS) (Fig. 3e–h). 15 genes were located in the LD block 31 region (Additional file 2: Fig. S10, Additional file 1: Tables S7 and S10). *Gh_D11G1919* had a higher expression level at − 3, 0, 3, 5, 10 DPA according to our transcript data. *Gh_D11G1919* worked as a CDPK-related kinase in the Signal transduction pathway, maybe regulating the fiber quality and yield traits in cotton.

**Figure 4:**
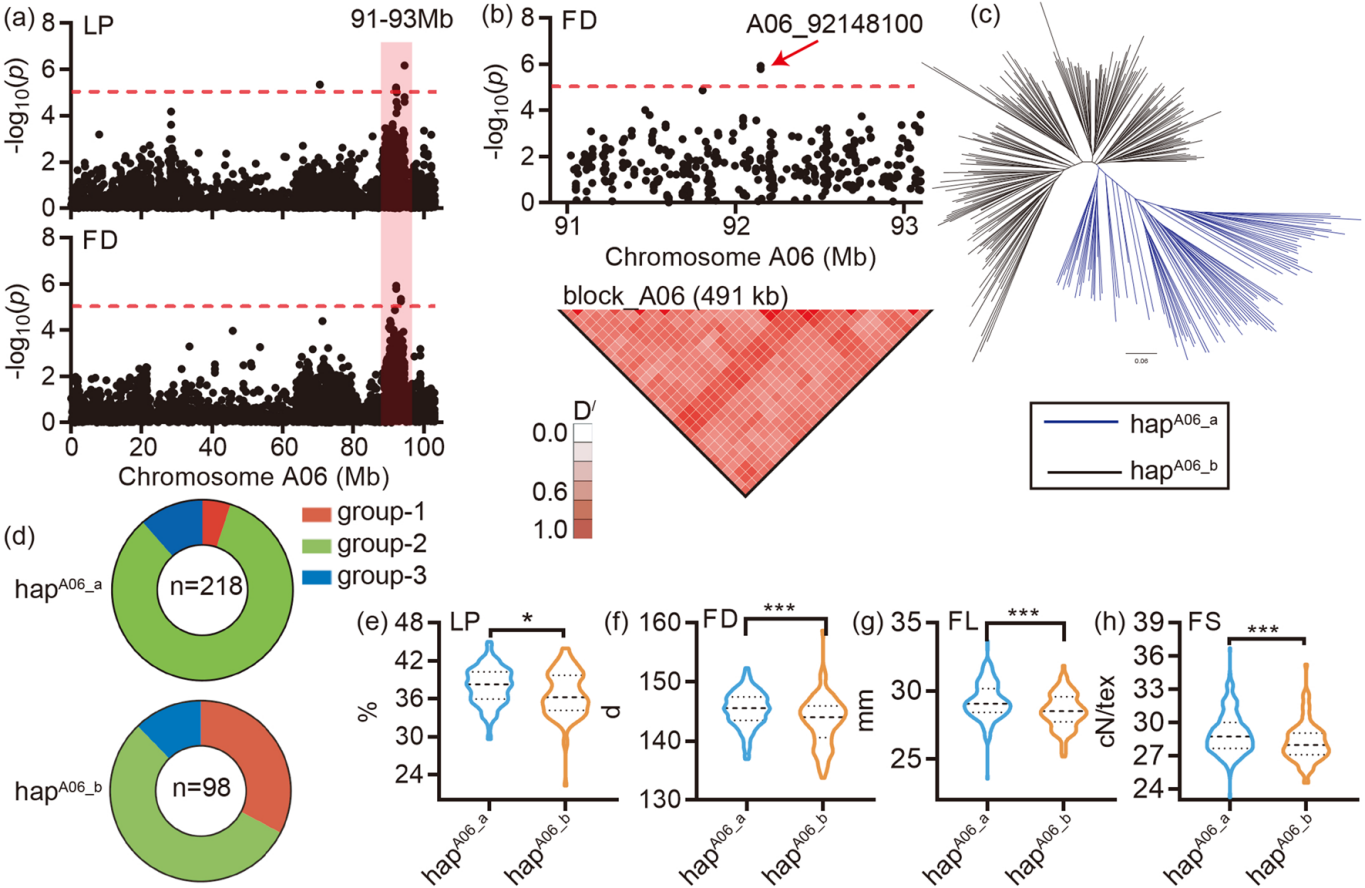
Identification and genotyping of the pleotropic region LD block 1 on chromosome A06. (**a**) The Manhattan block of the LP and FD, the horizontal dashed lines indicate the significance threshold (− log_10_(*P*) > 5.27). The red box indicates the region identified by the GWAS of LP and FD, located between 91 and 93 Mb on A06 chromosome; (**b**) Local Manhattan block (top) and LD heat map (bottom). The red arrows indicate the most significance SNP in the LD block at the top; (**c**) A neighbor-joining tree was constructed using the SNP data located in the LD block 3. The accessions were divided into two groups, hap^A06_a^ and hap^A06_b^; (**d**) The composition of the two haplotypes, group-1 (red), group-2 (cyan) and group-3 (blue); (**e**) Box plots for LP, FD, FL and FS for the two haplotypes (n = 218 and 98). Center line, median; box limits, upper and lower quartiles; whiskers, 1.5× the interquartile range; dots, outliers (*P < 0.05, **P < 0.01 and ***P < 0.001, two-sided t-test). The neighbor-joining tree (**c**) was constructed using the software PHYLIP (v3.696, https://evolution.genetics.washington.edu/phylip.html). The LD heatmap (the bottom of **b**) was created by the Haploview software (https://www.broadinstitute.org/haploview/haploview). The others were created by the software GraphPad Prism 9 (ver. 9.0.0, http://www.graphpad.com).

LD block 1 was located between 91.7 and 92.2 Mb on chromosome A06 and was associated with LP and FD (Fig. 4a,b). The phylogenetic tree was construct by the SNP involved in the LD block 3 (Fig. 4c), the result showed that 316 accessions could be divided into two group, hap^A06_a^ and hap^A06_b^. The group hap^A06_a^ had more accessions (218) than the group hap^A06_b^ (Fig. 4d). The genotyping results indicated that the group hap^A06_a^ had higher LP, better fiber quality (FL and FS) and later maturity, compared with hap^A06_b^ (Fig. 4e–h). 12 genes were located in the LD block 3 region (Additional file 2: Fig. S10, Additional file 1: Tables S10 and S13), *Gh_A06G1269* had high expression levels in leaf, according to our transcript data (Additional file 2: Fig. S10d). This gene was related to ‘Coenzyme transport and metabolism’ in COG, maybe work in the regulation of flowering date through the photoperiod pathway as its expression pattern in cotton.

### Elite accessions containing the pleiotropic regions

All 184 key SNPs were genotyped (Additional file 1: Table S11). Compared with the others, the SNPs included in LD blocks 6 and 8 were found in fewer accessions, indicting not have widely been used in cotton breeding (Fig. 2d,j).

By constructing phylogenetic trees, the 316 accessions were divided into two groups per pleiotropic region (Additional file 1: Table S14). Combine with the grouping result of all the four pleiotropic regions, the 316 accessions were then divided into five groups including class_1 (aaaa) to class_5 (bbbb) according to the number of elite haplotypes (Table 1).

**Table 1:** All the 316 accessions were classified to five new group, class_1 to class_5, according to the 4 pleotropic regions.

Group	Content
Class_1	aaaa^a^ (14)^b^
Class_2	aaab (8), aaba (19), abaa (36), baaa (2)
Class_3	bbaa (7), baba (12), baab (3), abba (93), abab (12), aabb (6)
Class_4	baaa (30), abaa (9), aaba (10), aaab (37)
Class_5	bbbb (18)

The number of hap^_a^ in the five groups were 4, 3, 2, 1, 0.

aaaa ^a^ = hapA06_a/hapA07_1_a/hapA07_2_a/hapD11_a. ^b^The number of the accessions belong to the aaaa was followed.

**Figure 5:**
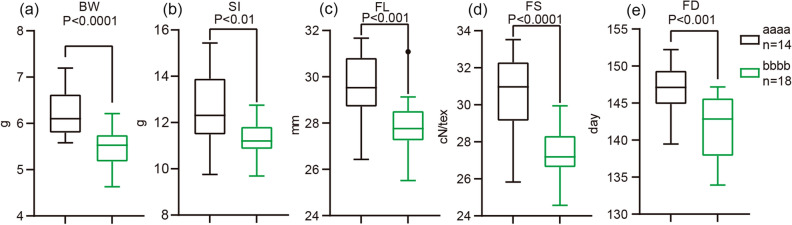
Genotyping of the five class (class_1 to class_5). (**a**–**e**) Violin plot for BW, SI, FL, FS and FD. Center line (red), median; box limits, upper and lower quartiles; whiskers, 1.5× the interquartile range. P values in this were derived with two-sided t-test. The figure was created by the software GraphPad Prism 9 (ver. 9.0.0, http://www.graphpad.com).

The genotyping results indicated that the accessions belong to class_1 had bigger BW and SI (Fig. 5a,b), better FL and FS (Fig. 5c,d), and longer FD (Fig. 5e) compared with class_5.

In short, the four pleiotropic regions showed additive effects. The 18 accessions belong to the class_5, with low fiber yield and poor fiber quality, were early maturing. The 14 accessions in the class_1 (Additional file 1: Table S14), carrying four elite pleiotropic haplotypes respectively, were identified as elite accessions with high yield (Fig. 5a,b) and superior fiber quality (Fig. 5c,d). The elite accessions mainly including zhongR and other big boll cotton, were obtained by mutation breeding and distant hybridization (Additional file 1: Table S14). Thus, the 14 elite accessions could be suggested to use in future cotton breeding to simultaneously improve fiber quality and yield.

## Discussion

GWAS has been used to identify trait-related regions recently, and many such regions have been reported^5,33,35^. For example, 119 trait-associated loci (71 for yield-related traits, 45 for fiber quality and three for resistance to Verticillium wilt) were identified by GWAS^5^. In particular, Ma et al. identified 7,383 unique SNPs and 4,820 genes associated with fiber-related traits^33^, and Du et al. identified 98 significant peak associations for 11 agronomically important traits in *G. arboretum*^35^.

Many GWAS have been done, and many loci and related genes have been identified, but nearly all of these loci are associated with one trait^36–39^. Some are associated with more than one trait, but all the trait belongs to yield trait and no effect on the fiber quality^5^. Few loci associated with both fiber quality and yield have been reported^32^. Our work identified four pleiotropic regions associated with fiber quality and yield, and 14 accessions carrying these pleiotropic regions. Among the four LD blocks we identified, LD blocks 6 and 8 were located on chromosome A07. Compared with other reports^5,32^, LD block 8 was smaller and was associated with more traits in our experiment. Additionally, LD block 6 has not been reported previously. The region from 24.02 to 24.09 Mb on chromosome D11 was reported to be associated with fiber length only^23^. In our study, LD block 31 (23.2–24.0 Mb) on chromosome D11 was considered as a pleiotropic region associated with BW and FL. LD block 1 on chromosome A06 was first identified to be associated with LP and FD in our work.

In contrast to other works, we also aimed to identify elite accessions carrying the four LD blocks (associated with yield, fiber quality and maturity). Consequently, we identified 14 accessions belonging to group class_1 and 65 accessions belonging to group class_2 with better fiber quality and higher yield.

Thus, GWAS on a natural population can identify many trait-related regions and elite accessions and is an efficient way to identify pleiotropic regions.

As previously reported, yield and fiber quality, which are the most important traits of cotton, were negatively correlated with each other for a long time after the introduction of Beasley’s triple hybrid to improve fiber strength^7^. Thus, the simultaneous improvement of yield and fiber quality has long been the goal of cotton breeding^8,9^. Culp et al. showed that the linkage drag of yield and fiber quality was disturbed^27^. However, the molecular mechanism underlying this linkage drag has not been reported yet.

As a result of the development of cotton breeding, their relationship of fiber quality and yield showed a positive correlation in our study. We analyzed 316 accessions, including many elite germplasm resources. The phenotypic data analysis showed that yield and fiber quality have become positively correlated. Significant positive correlations were found between BW and FL (0.30), BW and FS (0.37), and LP and MIC (0.35). Some pleiotropic regions were identified, providing an explanation for the molecular mechanism underlying the simultaneous improvement of yield and fiber quality.

Our genotyping showed that few accessions carried the pleotropic regions LD blocks 6 and 8 (Additional file 1: Table S12), indicating that these loci have not widely been used in cotton breeding. Further analysis showed that these accessions were mainly obtained by mutation breeding and distant hybridization. Thus, mutation breeding and distant hybridization could be two other methods for disrupting the linkage of yield and fiber traits.

## Conclusions

We identified four favorable pleiotropic loci and 14 elite accessions carrying the favorable loci, will be utilized in future cotton breeding for the improvement of yield and fiber quality.

## Methods

### Plant materials, planting and phenotyping

In total, 316 *Gossypium hirsutum* accessions (Additional file 1: Table S1) were selected from the National Cotton Mid-term Gene Bank (Anyang, Henan, China). Using previous investigation data, we comprehensively considered diversity of geographical origins, breeding years and phenotypic variety when selecting accessions^33^. The 316 accessions were divided into five cotton growing regions according to different ecological characteristics, including 34 accessions from Central Asia (CA), 47 from the United States (US), 162 from the Yellow River (YR), 52 from the Yangtze River (YZR) and 20 from other places (OTH) (Additional file 1: Table S2). Eight important traits were selected for GWAS. These traits were categorized into three groups, including yield components (boll weight/BW, seed index/SI, lint percentage/LP), fiber quality (fiber length/FL, fiber strength/FS, micronaire/MIC, fiber elongation/FE) and maturation (flowering date/FD) of *Gossypium hirsutum* (Additional file 1: Table S1). All 316 varieties were grown in three representative cotton production regions of China, including Anyang in Henan Province (representing the Huanghe River production zone), Nanjing in Jiangsu Province (representing the Yangtze River production zone) and Kuche in Xinjiang Province (representing the Xinjiang production zone). All the accessions were grown in the same fields with three replications for three continuous years (2007–2009). The nine environments (three locations x three years) were defined as: 1, AY_2007; 2, NJ_2007; 3, Kuche_2007; 4, AY_2008; 5, NJ_2008; 6, Kuche_2008; 7, AY_2009; 8, NJ_2009; 9, Kuche_2009. Each plot contained one row 7 m in length and 76 cm between rows, with 20–22 plants per row at the Anyang and Nanjing location (33 cm between plants with each row), whereas each plot contained 60–66 plants per row at the Kuche location (10 cm between plants with each row)^33^. All field management, including watering, weed management, and fertilization, was performed according to the usual local management in each test location for all accessions during the growing period.

All trait measurements followed the standards described in the Descriptors and Data standard for cotton (*Gossypium *spp.). FD was calculated from the sowing day to the day that 50% of the plants had appeared the first flower in per plot. Thirty naturally mature bolls from each accession were hand-harvested to calculate BW and to gin the fiber. The SI was obtained by weighing 100 cotton seeds. Fiber samples were separately weighed for calculating LP, MIC, FL, FS, FE. The average of the three replicates in the same environment were calculated as the phenotypic data for each accession for phenotypic analysis^33^.

All data statistics, including Student’s two-tailed t test and two-way analysis of variance (ANOVA), were calculated by GraphPad Prism 9 (ver. 9.0.0, http://www.graphpad.com). Pearson’s correlation coefficient between pairwise phenotypes was obtained with the R software^40^, using the package “psych”^41^.

### Sampling, DNA extraction and RAD sequencing

In 2012, we planted seeds of all 316 accessions in small pots in a greenhouse for DNA extraction, five full seeds for each accession. After the two cotyledons spread, the seedlings were collected and frozen at − 80 °C immediately^33^. Genomic DNA was extracted following the CTAB method with some modifications^42^. The DNA was quantified on a Qubit 2.0 fluorometer. After the concentration was calculated, the genomic DNA was diluted to 50 ng/μl, and 1 μg of each sample was transferred to a clean 200 μL PCR plate (Axygen). The genomic DNA in each well was digested with 1 μl Fast Digest TaqI (Fermentas) for 10 min at 65 °C in a volume of 30 μl. For ligation reactions, 1 μl of barcoded adapters (10 μM) were added to each well, along with T4 DNA ligase (Fermentas), in a total volume of 40 μl. The ligation reaction was incubated for 1 h at 22 °C and heat-inactivated at 65 °C for 20 min. The ligation products for 24 different samples were pooled into a single tube, and 2 µl chloroform was added to inactivate the restriction enzyme. The mixtures were centrifuged at 12,000×*g* for 1 min and the supernatant was transferred to a new tube. DNA fragments between 400 and 700 bp were selected on a 2% agarose gel (Amresco) and purified using a QIA quick Gel Extraction Kit (Qiagen). The samples were resuspended in 50 elution buffer and amplified with 10 cycles of PCR. The PCR reaction included 8 μl of library DNA, 25 μl of Phusion Master Mix (Finnzymes), 1 μl common primer (10 μM), 1 μl index primer (10 μM) and 15 μl water. The amplified library was purified using a QIA quick PCR Purification Kit (Qiagen), further quantified on the Agilent 2100 Bioanalyzer and sequenced on an Illumina Hiseq 2000 instrument.

### Alignment and genotyping

The paired end reads from each individual were identified by their barcodes and aligned against the reference genome^5^ using the software BWA v0.5.9^43^. Samtools v0.1.18^44^ was used to generate consensus sequences for each individual and prepare input data for SNP calling with realSFS based on Bayesian estimation. SNPs matching the following criteria were removed: (1) Raw SNPs with sequencing depth > 6500× or < 60 ×  (SNPs with extremely high sequencing depth most likely result from repetitive regions or alignment errors). (2) When the length between two adjacent SNP loci was < 5 bp. (3) SNPs with a call rate < 70% in the whole population. (4) Minor allele frequency (MAF) was > 0.05. (5) The proportion of heterozygous genotypes was > 30%. SNP locations in the genome were categorized into different regions according genomic annotations, including noncoding, CDS, intron, UTR and 1000 bp up/downstream of gene.

### Population genetic analysis and GWAS

A pairwise distance matrix derived from the simple matching distances for all SNPs was calculated to construct unweighted neighbor joining trees using the software PHYLIP v3.696^45^. Population structure was determined using the software Admixture v1.30 with default settings^46^. Association analysis was conducted using the EMMA eXpedited (EMMAX) software package^34^. The EMMAX package was downloaded from http://csg.sph.umich.edu//kang/emmax/download/index.html. A total of 93,687 high-quality SNPs (MAF > 0.05, missing rate < 20%) in 316 *G. hirsutum* accessions were used to perform GWAS for eight traits under ten environments (Additional file 1: Table S1) in EMMAX software. With the help of the emmax-kin-intel package of EMMAX, Population stratification and hidden relatedness were modeled with a kinship (K) matrix^35^.

The multi-locus GWAS was conducted by the software mrMLM.GUI (version 4.02) to verify the SNPs identified by single-locus GWAS^47^. The mrMLM package was downloaded from https://cran.r-project.org/web/packages/mrMLM.GUI/index.html^48–53^.

In this study, the *P* value was used to define the threshold signals of the GWAS, for which we used a significance cutoff of 5.34e−6 (0.5/n, − log P = 5.27)^35^.

### RNA sample collection, extraction and sequencing

Two accessions were selected for RNA-Seq, J02-508 with higher yield and better fiber quality compared with Zhong870203, sampled at root, steam, leaf, − 3, 0, + 3, + 5, + 10, + 15, + 20, and + 25 DPA. Sample collection was performed as described in Zou’s research^54^ and RNA extraction followed the instructions of RNA Isolation Kit (Tiangen Biotech).

After be qualified, the RNA sample was used to construct RNA sequencing library. The main process was as follows: First, rRNA was eliminated by the Ribo-Zero rRNA Removal Kit (Epicentre); second, rRNA-depleted RNA was broken with the help of Fragmentation Buffer; third, cDNA Synthesis; and fourth, end repair, add 3ʹ A tail, ligate adapters, PCR, sequencing was carried out on Illumina HiSeq X Ten platform. After filtering adapter and low-quality reads, the clean data was got and used to mapped onto the genome of Upland cotton^5^, using HISAT2 (ver. 2.2.0). Gene expression levels (FPKM) were calculated using StringTie (ver. 2.13)^39^.

### LD block and candidate gene finding

As a confidence interval, linkage disequilibrium (LD) decay was used to identify LD blocks and candidate genes. LD blocks of all the significance SNPs (− log_10_(*P*) > 5.27) were calculated on the whole genome level by the Haploview software, with the ‘Solid Spine of LD’ algorithm^55^. If the LD block contained SNPs associated with multi-trait, then it was defined as the pleiotropic loci. The expression patterns of the genes in the LD blocks were obtained from the data of Zhang^56^ and our own RNA sequencing. Genes with specific expression patterns in specific tissues or development stages were supposed to affect related traits^32^.

### Favorable pleotropic haplotype identification

Among the 27 LD block obtained by the LD block analysis above, four LD block were associated with multi-traits and considered as pleotropic LD block.

For per LD block identified above, the favorable haplotype definition was described as follow:

The genotype of the SNP locating in the LD block were filtered and considered as input data in the TASSEL 5 (ver. 20200709). By constructing phylogenetic tree, 316 accessions were classified into two groups, hap^_a^ and hap^_b^. The favorable haplotype was identified through phenotyping by the software GraphPad Prism 9 (ver. 9.0.0, http://www.graphpad.com).

## Supplementary Information

Supplementary Information 1.

Supplementary Information 2.

Supplementary Information 3.

Supplementary Information 4.

## Data Availability

All raw sequencing data are available from the SRA database under the following project number: PRJNA353524. The RNA-Seq data from Zhang’s research were downloaded from the NCBI Sequence Read Archive (http://www.ncbi.nlm.nih.gov/sra/) under accession PRJNA248163^56^.

## Abbreviations

BW: Boll weight
SI: Seed index
LP: Lint percentage
FL: Fiber length
FS: Fiber strength
FE: Fiber elongation
MIC: Micronaire
FD: Flowering data
GWAS: Genome-wide association study
LD: Linkage disequilibrium
RAD-seq: Restriction-site-associated DNA sequencing
CV: Coefficient of variation
BLUP: Best Linear Unbiased Prediction
CA: Central Asia region
YR: Yellow River region
US: United States
YZR: Yangtze River region
OTH: Other regions
