# Supplementary material for: Favorable pleiotropic loci for fiber yield and quality in upland cotton (Gossypium hirsutum)
Source: Sci Rep. 2021 Aug 5;11:15935. doi: 10.1038/s41598-021-95629-9 (PMC8342446; doi:10.1038/s41598-021-95629-9)
Supplement: Supplementary file 1 — Supplementary Information 1. [file 41598_2021_95629_MOESM1_ESM.docx]

**Supplementary information**

**Additional file 1:** **Table S1**. The result of the trait used in GWAS. **Table S2**. Statistics of all the phenotypical data. **Table S3** Sum of squares of deviation from mean (SS) of the 8 agronomic traits measurements in 9 environments. **Table S4**. All 316 accessions used in this study. **Table S5**. Number of accessions and geographic origin in different group. Central Asia (CA), the United States (US), the Yellow River (YR), the Yangtze River (YZR) and other places (OTH). **Table S6**. All the SNP, -log_10_(*P*) not less than 5.27, used in our study. **Table S7**. All the SNP, -log_10_(*P*) not less than 5.27, identified by multi-locus GWAS in our study. **Table S8**: The key SNPs associated with yield, fiber quality and FD. **Table S9**. The location of the key SNPs in 26 chromosomes. **Table S10**. The expression profiles of genes located in the 27 LD block. The RNA-Seq data were from Zhang’s research, downloaded from the NCBI Sequence Read Archive under accession PRJNA248163. **Table S11**. The four LD block were predicted to be associated with more than one trait. **Table S12**. Genotyping of 231 key SNPs identified in our study. **Table S13**. The expression pattern and annotation of the gene involved in the four pleotropic regions according to our transcript data. J02-508 with higher yield and better fiber quality compared with Zhong870203. **Table S14**. The five groups classified by the combination of the haplotype of the four pleotropic regions.

**Additional file 2: Fig. S1**: (a) The percent of SS (sum of squares of deviation from mean) show the effect of Environment (E), Genotype (G) and G x E interaction. (b) Phenotypic analysis reveals trait relationships. **Fig. S2**: Pearson`s correlation coefficients of per trait under nine environments, each trait result under nine environments were used as input data. **Fig. S3**: Pearson`s correlation coefficients of the eight traits in per nine environments. **Fig. S4**: The Manhattan block of FL_blup、SI_blup、FS_blup and BW_blup, the black horizontal lines indicate the significance threshold (-log_10_(*P*) > 5.27), rectangular areas indicate the pleotropic regions on chromosome A07. **Fig. S5**: The Manhattan block of BW_AY_2007、BW_NJ_2007、BW_AY_2008 and BW_NJ_2008, the black horizontal lines indicate the significance threshold (-log_10_(*P*) > 5.27). **Fig. S6**: The Manhattan block of FL_NJ_2008、FL_AY_2009 and FL_NJ_2009, the black horizontal lines indicate the significance threshold (-log_10_(*P*) > 5.27). **Fig. S7**: The Manhattan block of FS_Kuche_2007、FS_AY_2008、FS_NJ_2008、FS_AY_2009 and FS_NJ_2009, the black horizontal lines indicate the significance threshold (-log_10_(*P*) > 5.27). **Fig. S8**: The Manhattan block of SI_AY_2007、SI_NJ_2007、SI_Kuche_2007、SI_AY_2008 and SI_NJ_2008, the black horizontal lines indicate the significance threshold (-log_10_(*P*) > 5.27). **Fig. S9**: The Manhattan block of SI_Kuche_2008、SI_AY_2009、SI_NJ_2009 and SI_Kuche_2009, the black horizontal lines indicate the significance threshold (-log_10_(*P*) > 5.27). **Fig. S10**: (a) The expression pattern of the genes involved in the four pleotropic regions, according to our own transcriptome data. (b) The expression pattern of the genes involved in the four pleotropic regions, according to zhang’s transcriptome data. (c) The expression pattern of the candidate genes involved in the pleotropic regions on chromosome A07. (d) The expression pattern of the candidate genes involved in the pleotropic regions on chromosome A06. **Fig. S11**: (a) The Manhattan block of LP_Kuche_2007 and FD_Kuche_2007, (b) The Manhattan block of BW_Kuche_2007 and FL_Kuche_2007, the black horizontal lines indicate the significance threshold (-log_10_(*P*) > 5.27).

**Additional file 3:** All the basic Manhattan plot used in this study.
