# Supplementary material for: Favorable pleiotropic loci for fiber yield and quality in upland cotton (Gossypium hirsutum)
Source: Sci Rep. 2021 Aug 5;11:15935. doi: 10.1038/s41598-021-95629-9 (PMC8342446; doi:10.1038/s41598-021-95629-9)
Supplement: Supplementary file 3 — Supplementary Information 3. [file 41598_2021_95629_MOESM3_ESM.docx]

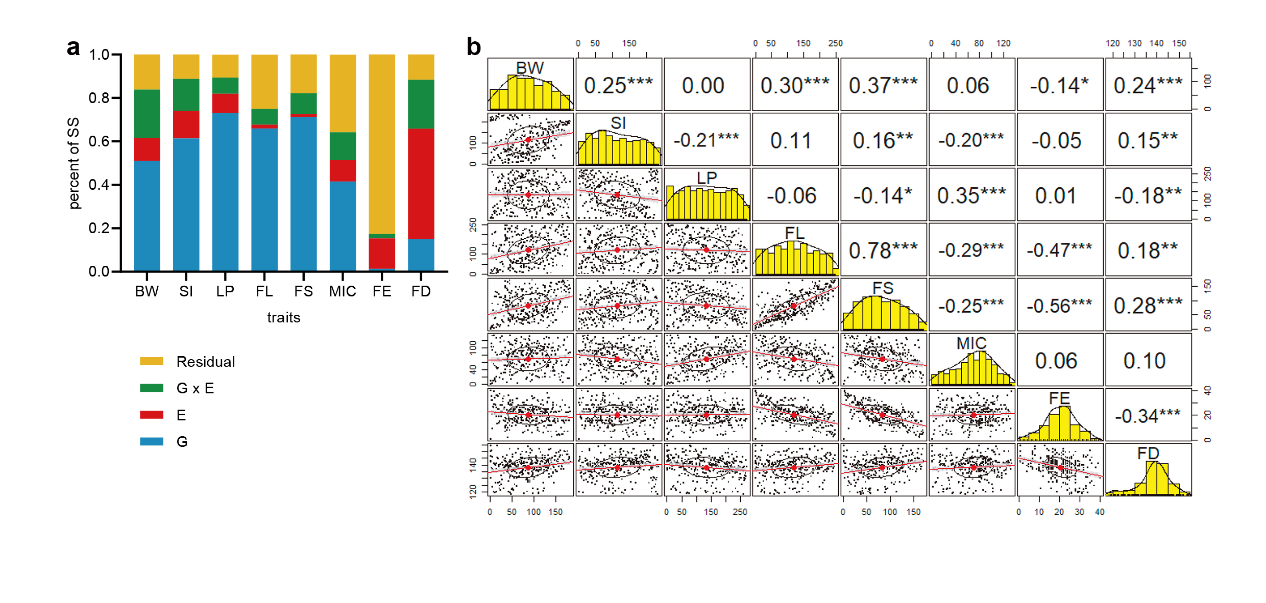


Fig. S1: **a** The percent of SS (sum of squares of deviation from mean) show the effect of Environment (E), Genotype (G) and G x E interaction. **b** Phenotypic analysis reveals trait relationships.


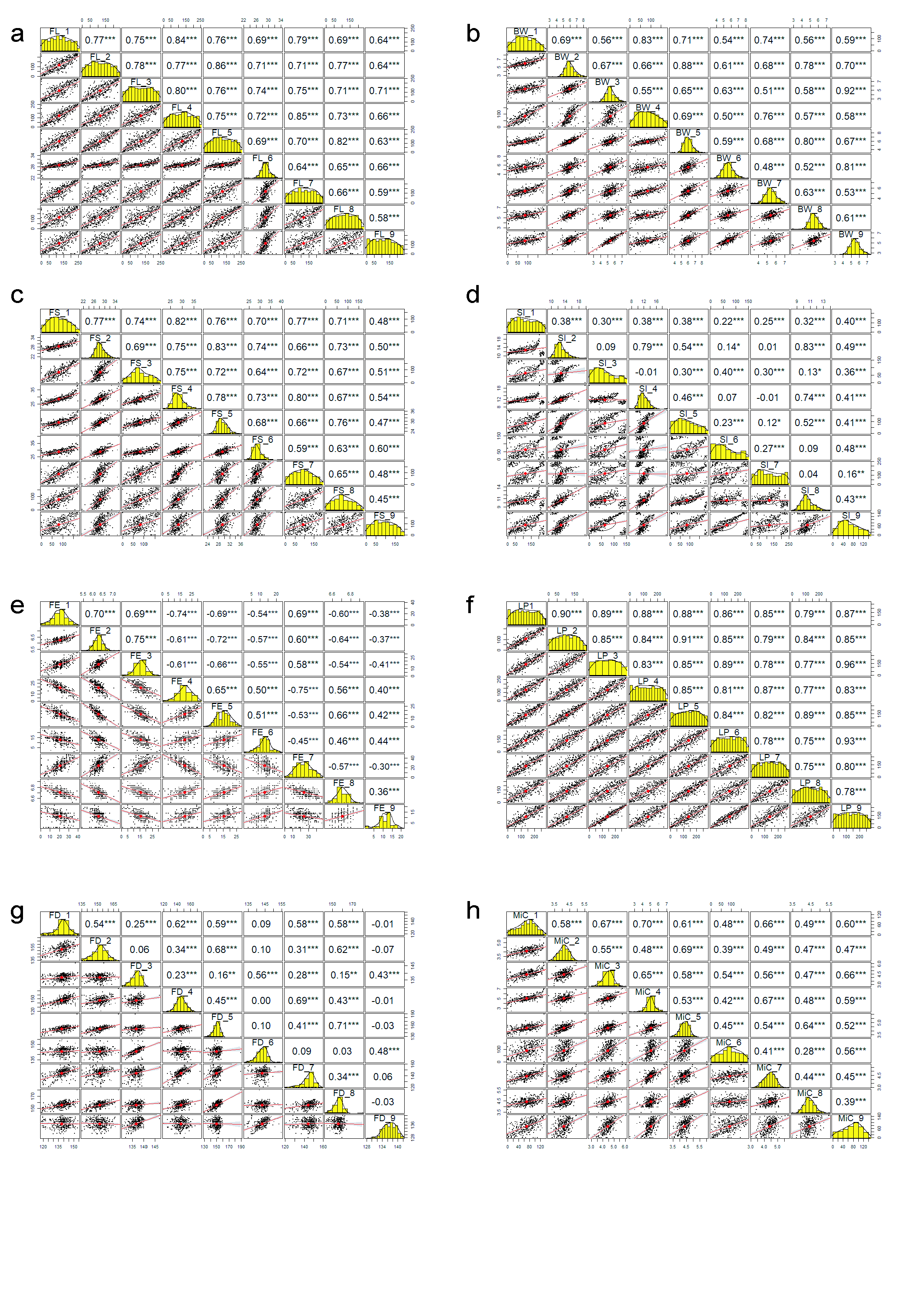


Fig. S2: Pearson`s correlation coefficients of per trait under nine environments, each trait result under nine environments were used as input data.


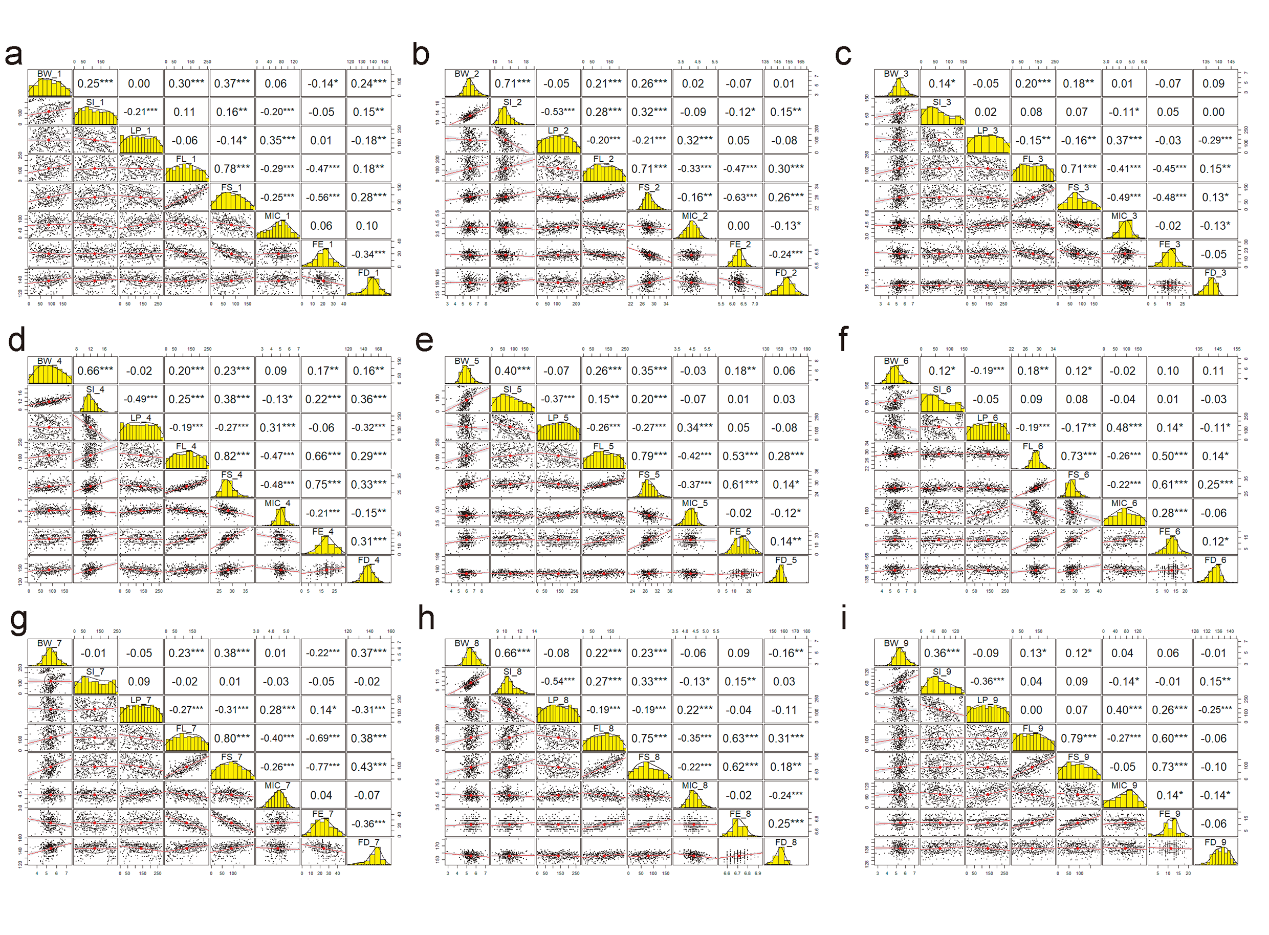


Fig. S3: Pearson`s correlation coefficients of the eight traits in per nine environments.


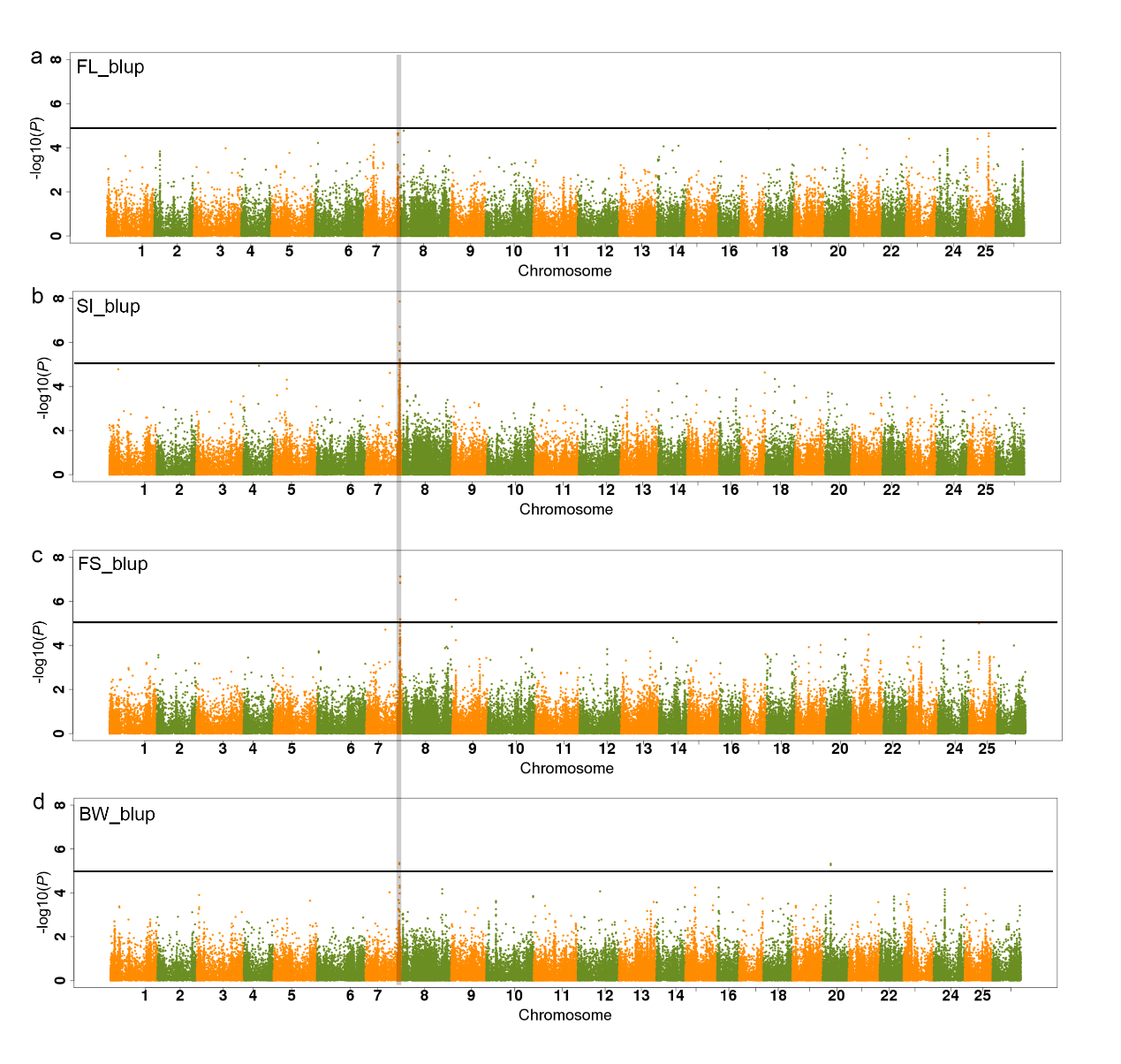


Fig. S4: The Manhattan block of FL_blup、SI_blup、FS_blup and BW_blup, the black horizontal lines indicate the significance threshold (-log_10_(*P*) > 5.27), rectangular areas indicate the pleotropic regions on chromosome A07.


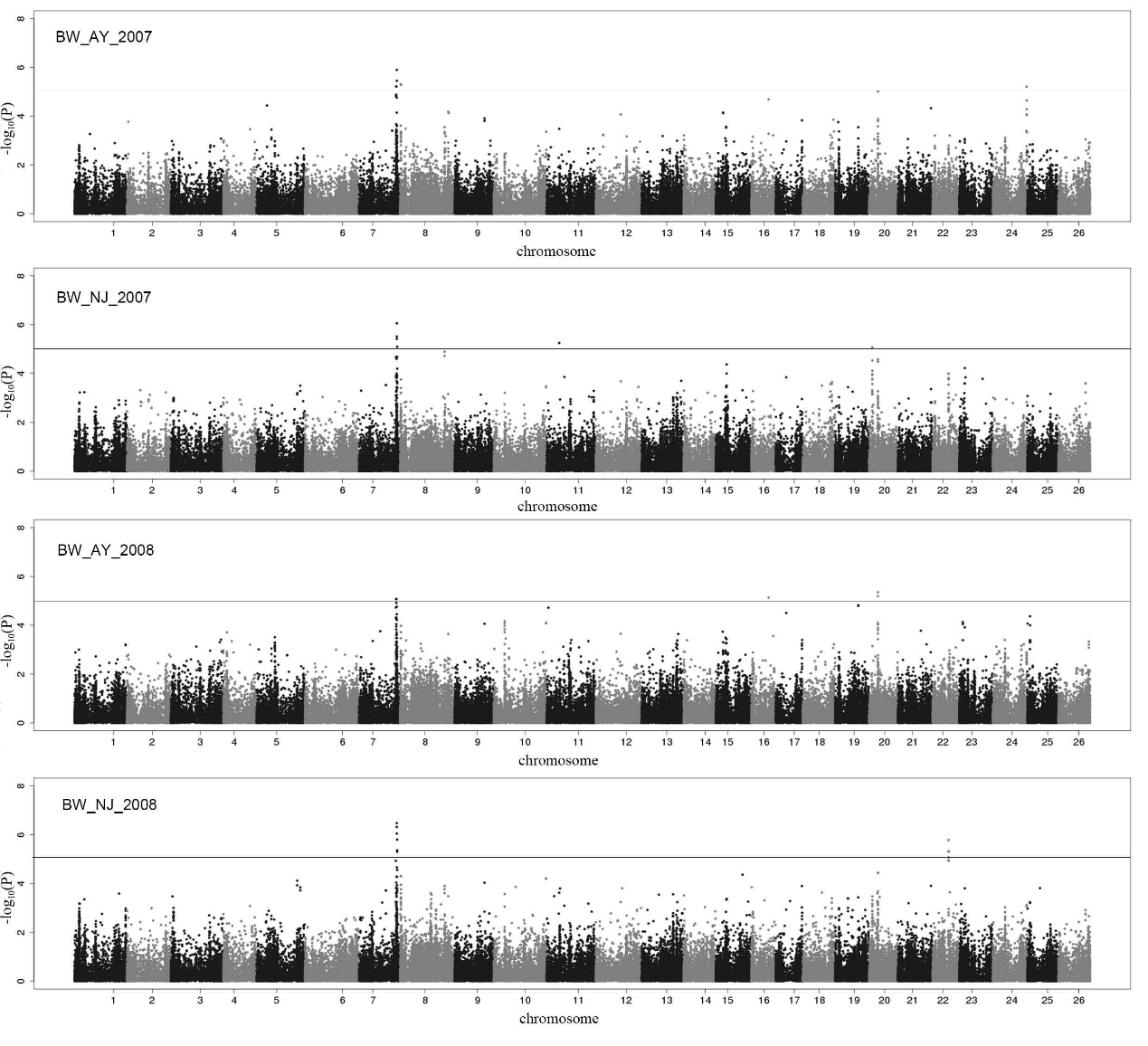


Fig. S5: The Manhattan block of BW_AY_2007、BW_NJ_2007、BW_AY_2008 and BW_NJ_2008, the black horizontal lines indicate the significance threshold (-log_10_(*P*) > 5.27).


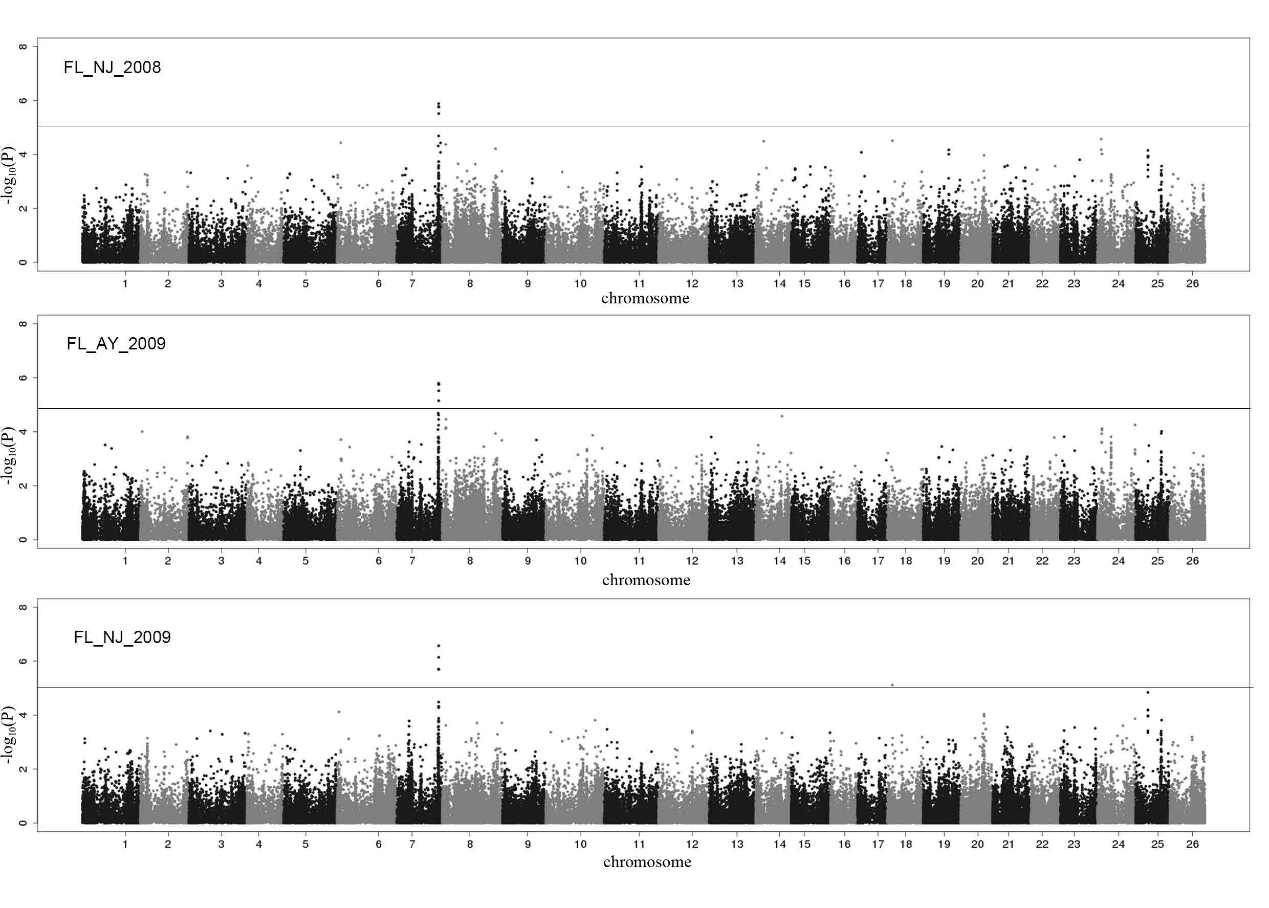


Fig. S6: The Manhattan block of FL_NJ_2008、FL_AY_2009 and FL_NJ_2009, the black horizontal lines indicate the significance threshold (-log_10_(*P*) > 5.27).


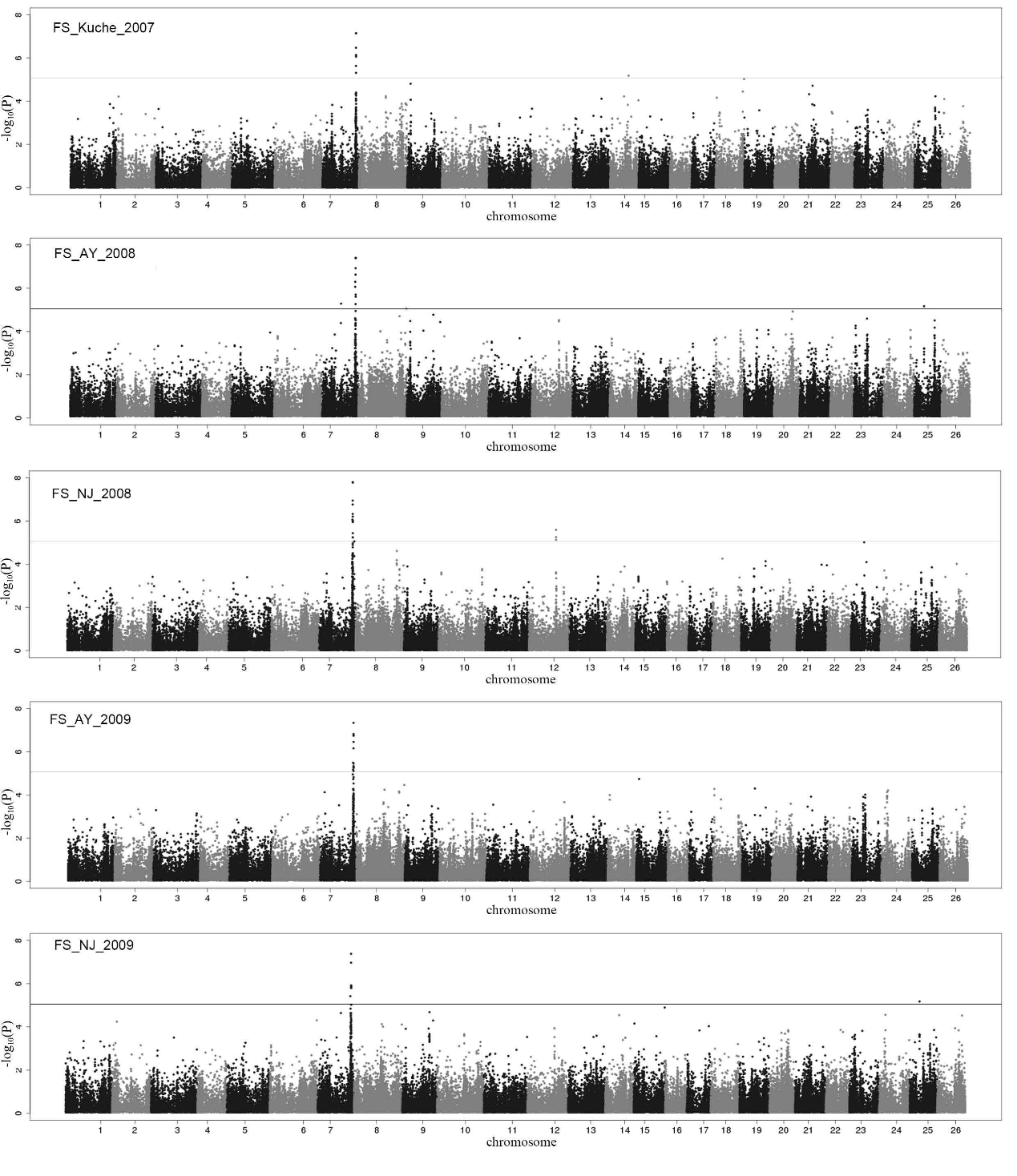


Fig. S7: The Manhattan block of FS_Kuche_2007、FS_AY_2008、FS_NJ_2008、FS_AY_2009 and FS_NJ_2009, the black horizontal lines indicate the significance threshold (-log_10_(*P*) > 5.27).


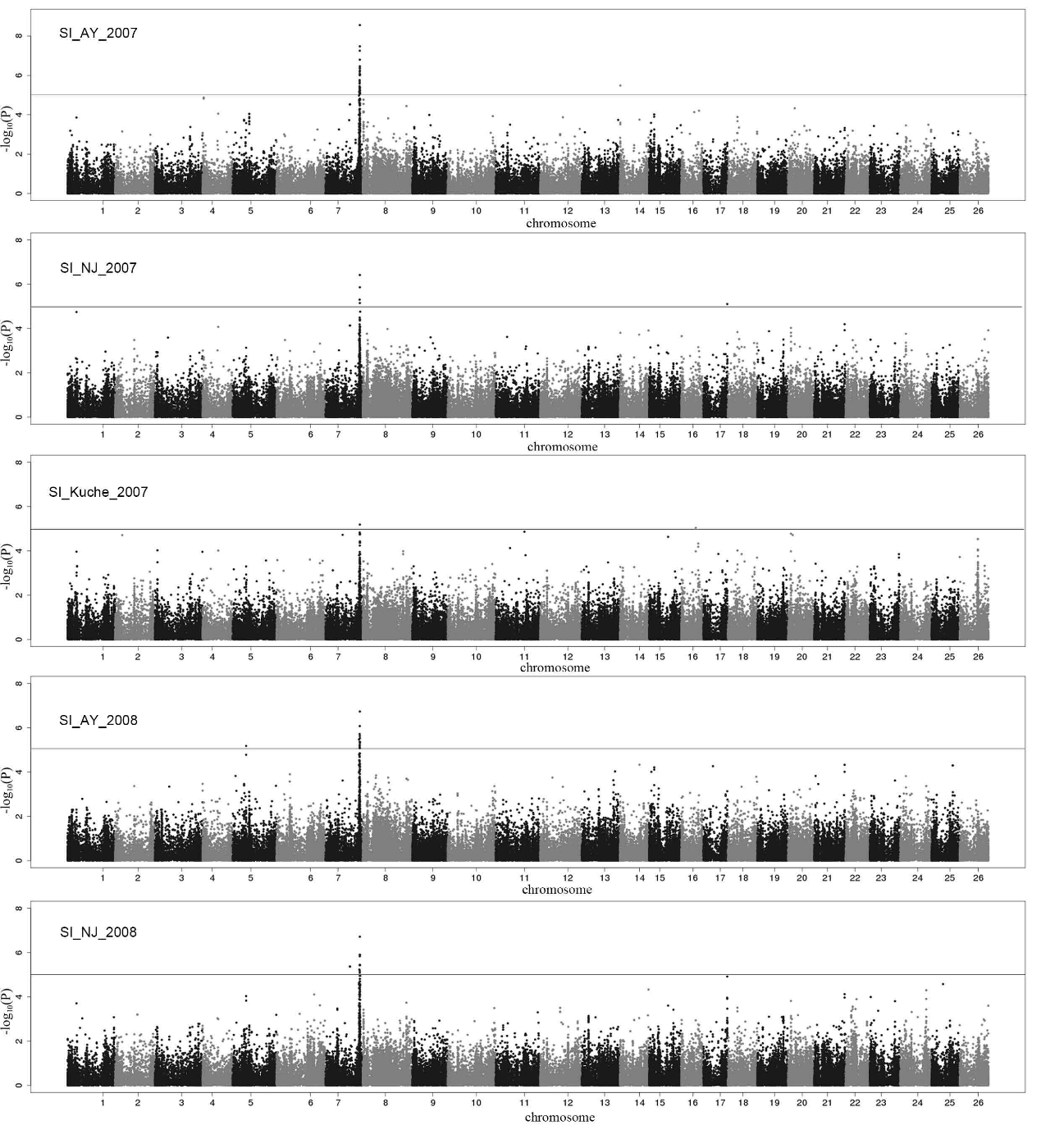


Fig. S8: The Manhattan block of SI_AY_2007、SI_NJ_2007、SI_Kuche_2007、SI_AY_2008 and SI_NJ_2008, the black horizontal lines indicate the significance threshold (-log_10_(*P*) > 5.27).


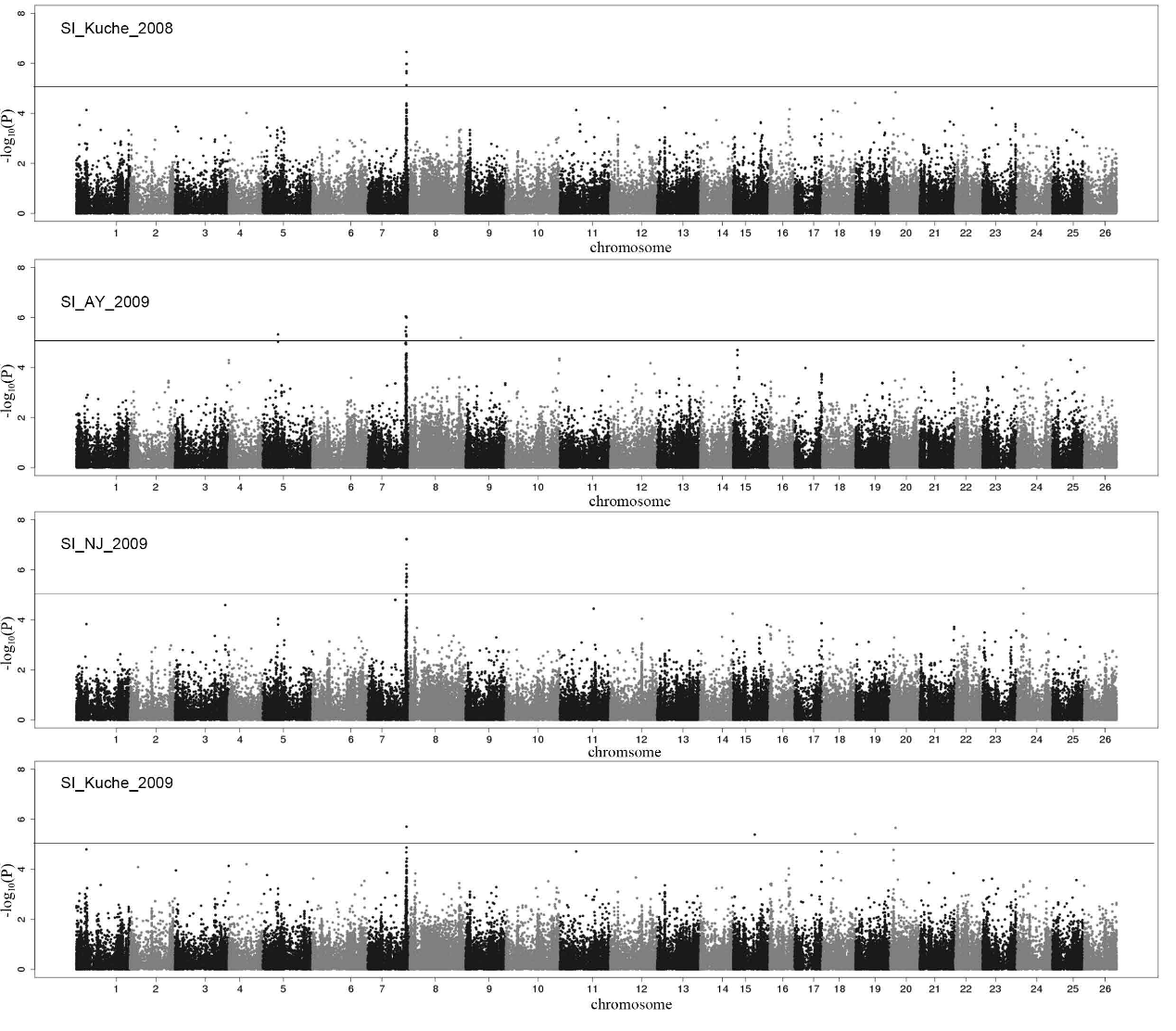


Fig. S9: The Manhattan block of SI_Kuche_2008、SI_AY_2009、SI_NJ_2009 and SI_Kuche_2009, the black horizontal lines indicate the significance threshold (-log_10_(*P*) > 5.27).


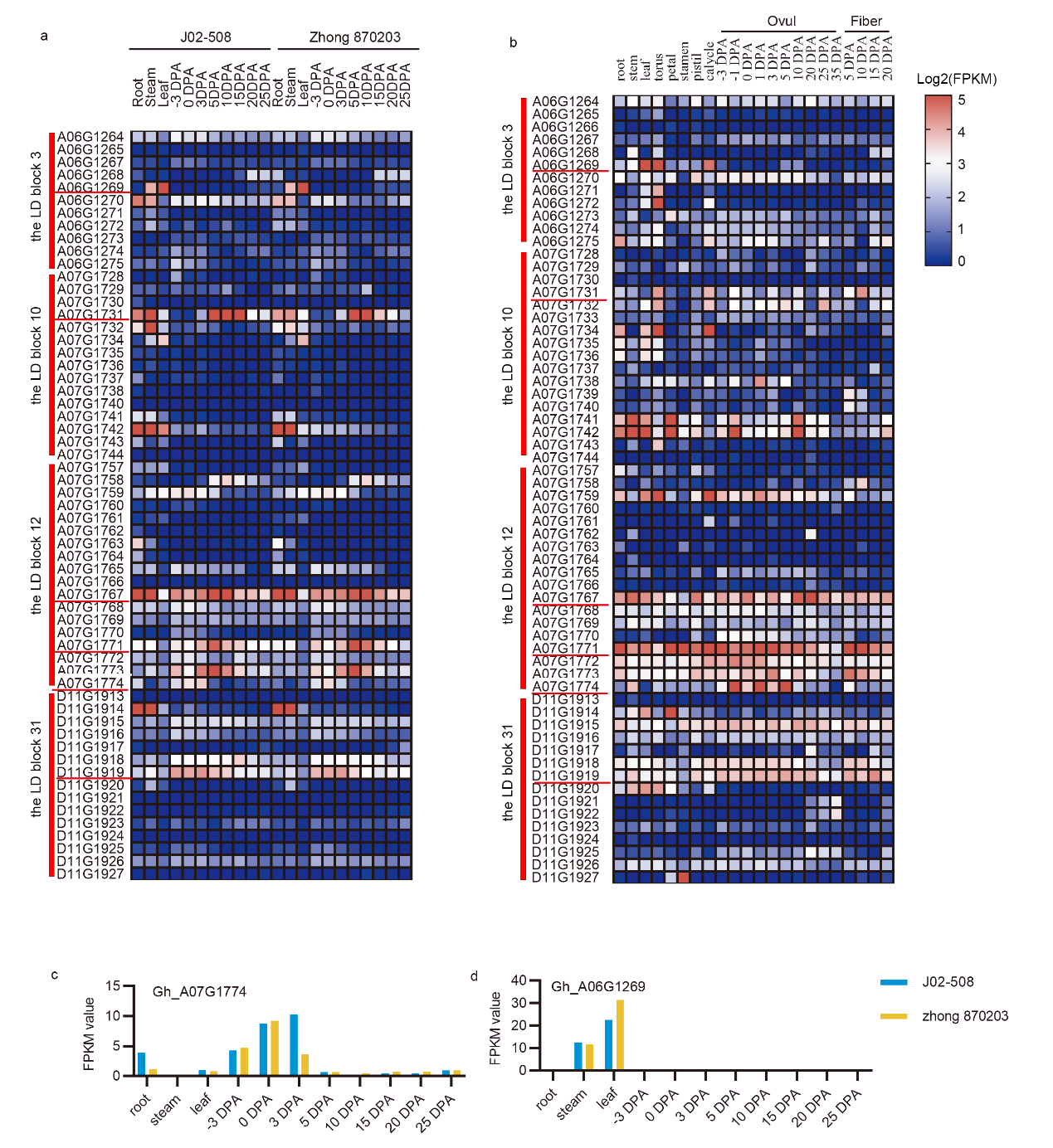


Fig. S10: a The expression pattern of the genes involved in the 4 pleotropic regions, according to our own transcriptome data. b The expression pattern of the genes involved in the 4 pleotropic regions, according to zhang’s transcriptome data. c The expression pattern of the candidate genes involved in the pleotropic regions on chromosome A07. d The expression pattern of the candidate genes involved in the pleotropic regions on chromosome A06.


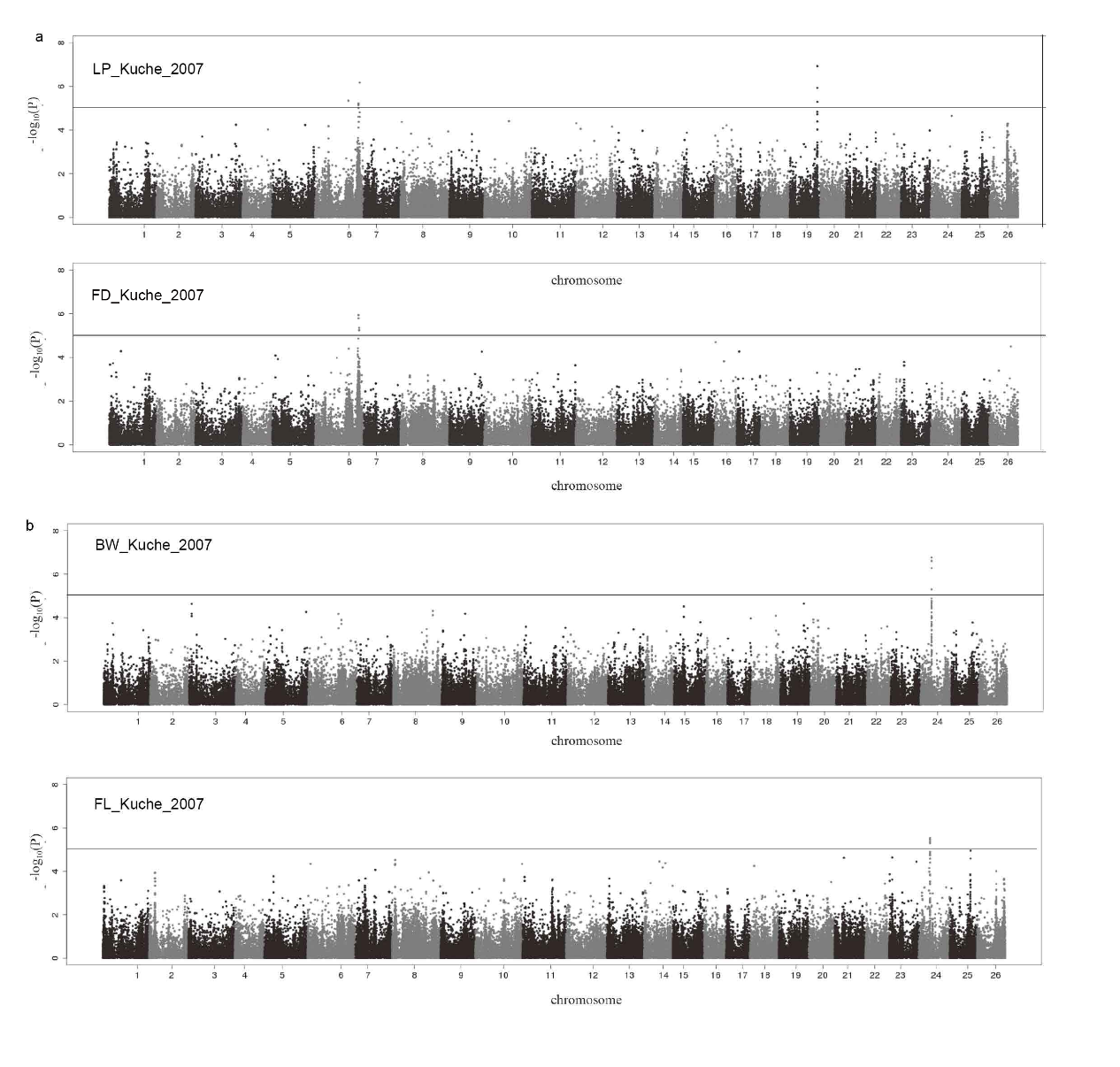


**Fig. S11**: a The Manhattan block of LP_Kuche_2007 and FD_Kuche_2007, b The Manhattan block of BW_Kuche_2007 and FL_Kuche_2007, the black horizontal lines indicate the significance threshold (-log_10_(*P*) > 5.27)
