# Supplementary material for: Favorable pleiotropic loci for fiber yield and quality in upland cotton (Gossypium hirsutum)
Source: Sci Rep. 2021 Aug 5;11:15935. doi: 10.1038/s41598-021-95629-9 (PMC8342446; doi:10.1038/s41598-021-95629-9)
Supplement: Supplementary file 4 — Supplementary Information 4. [file 41598_2021_95629_MOESM4_ESM.docx]

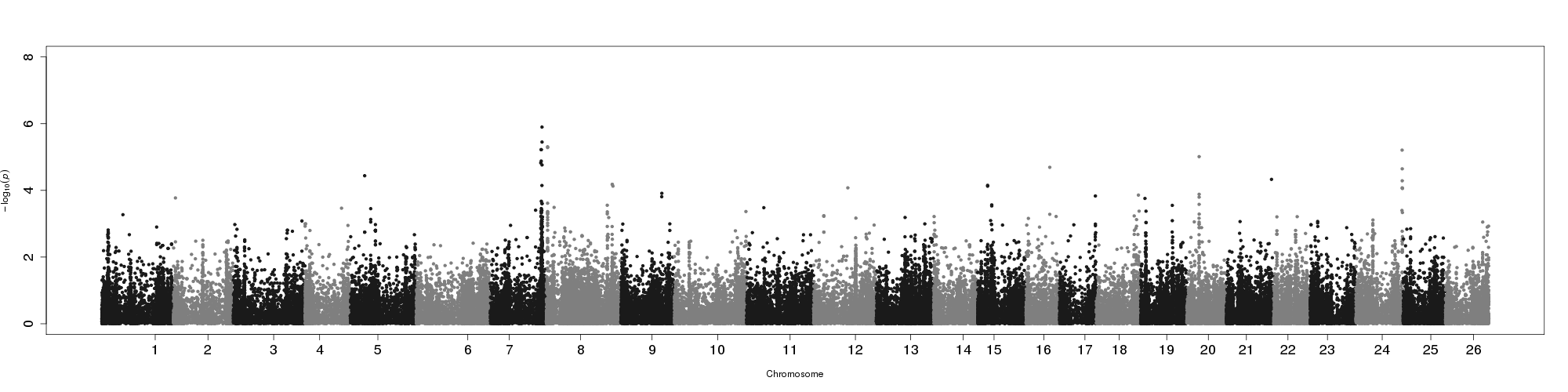

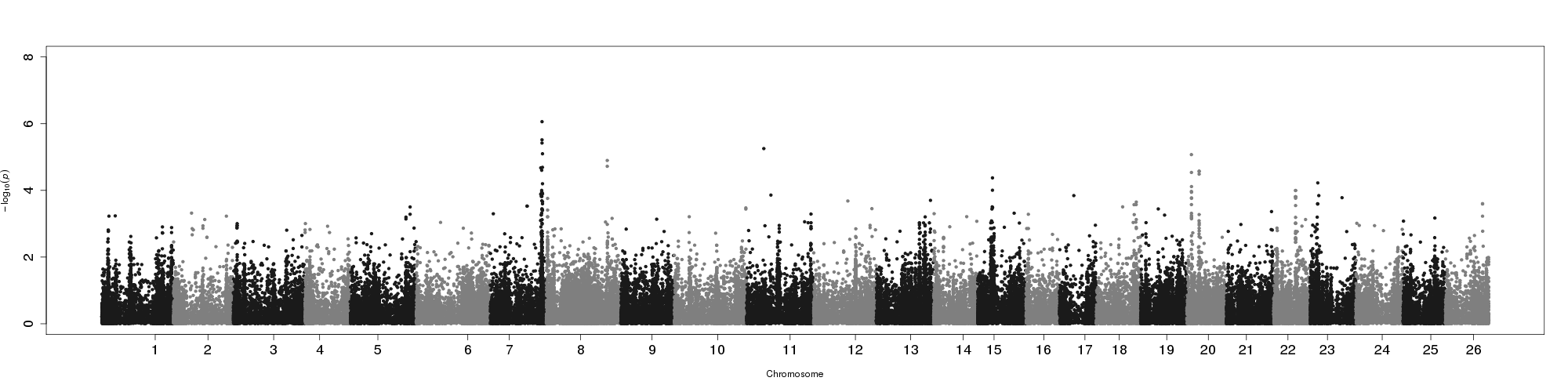

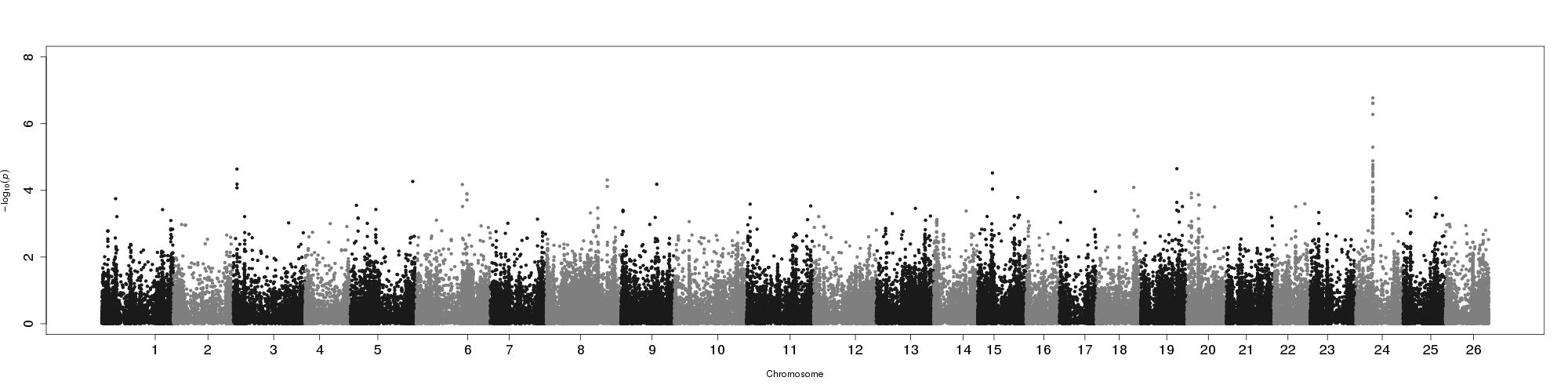

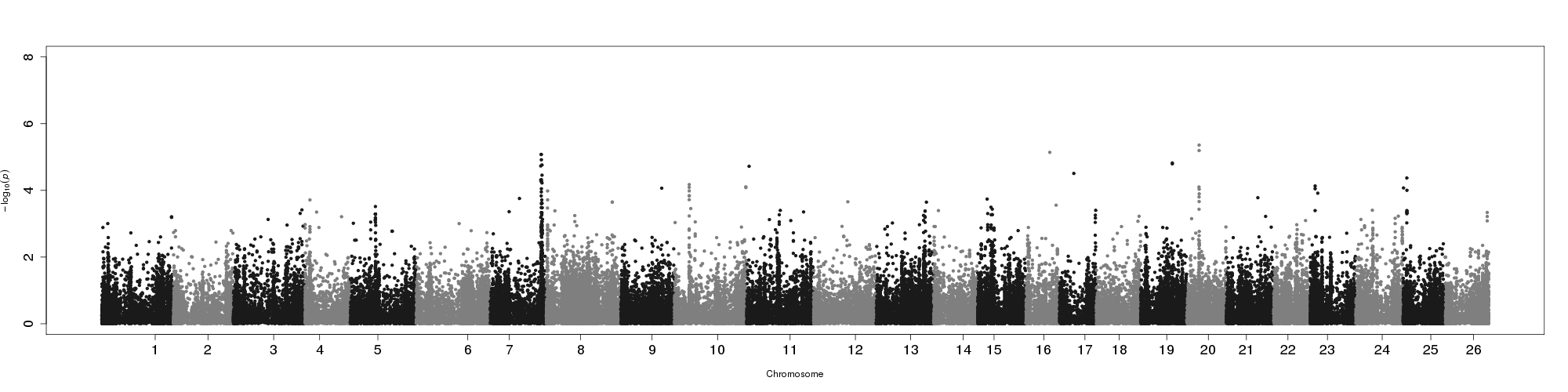

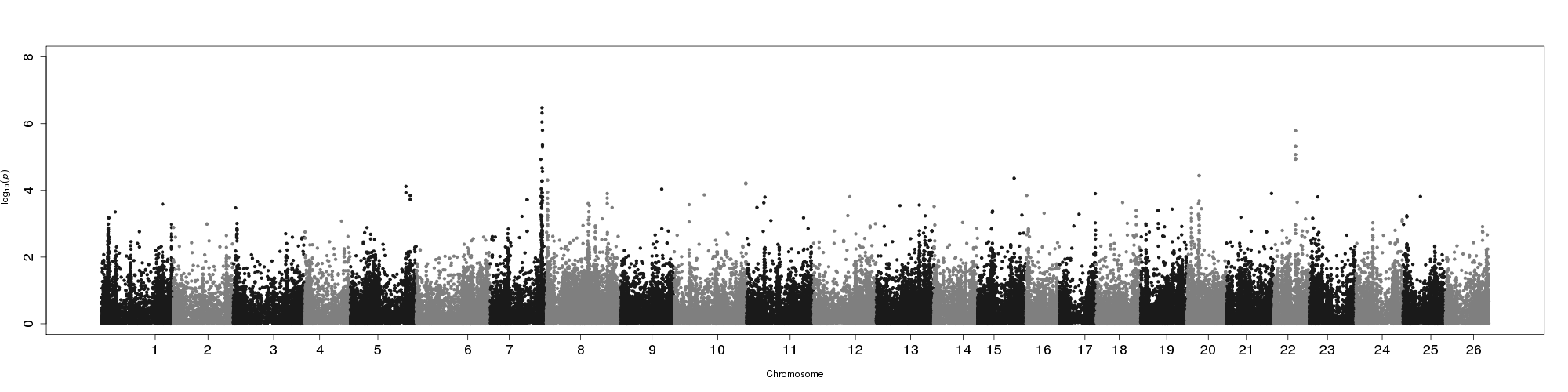

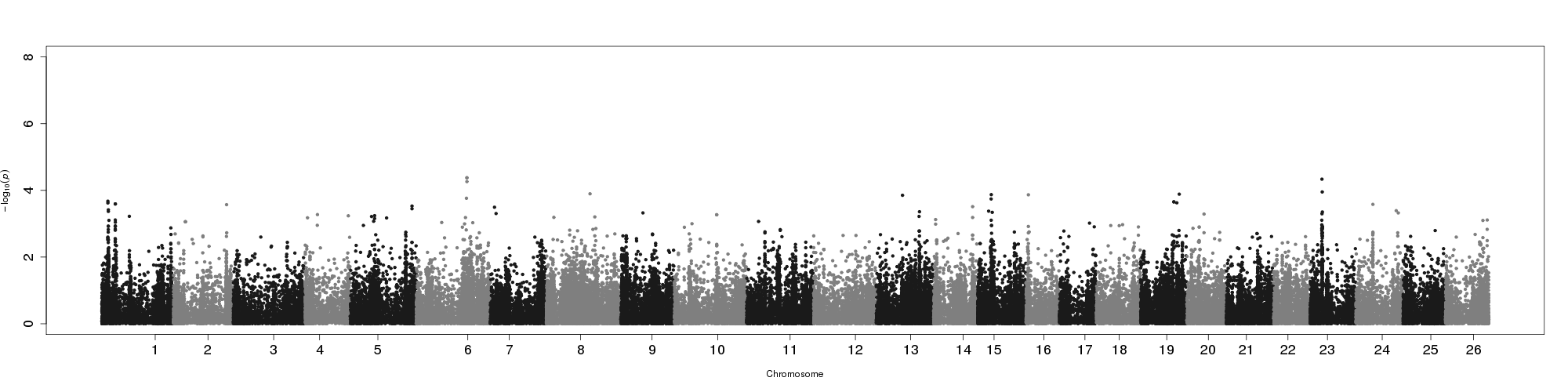


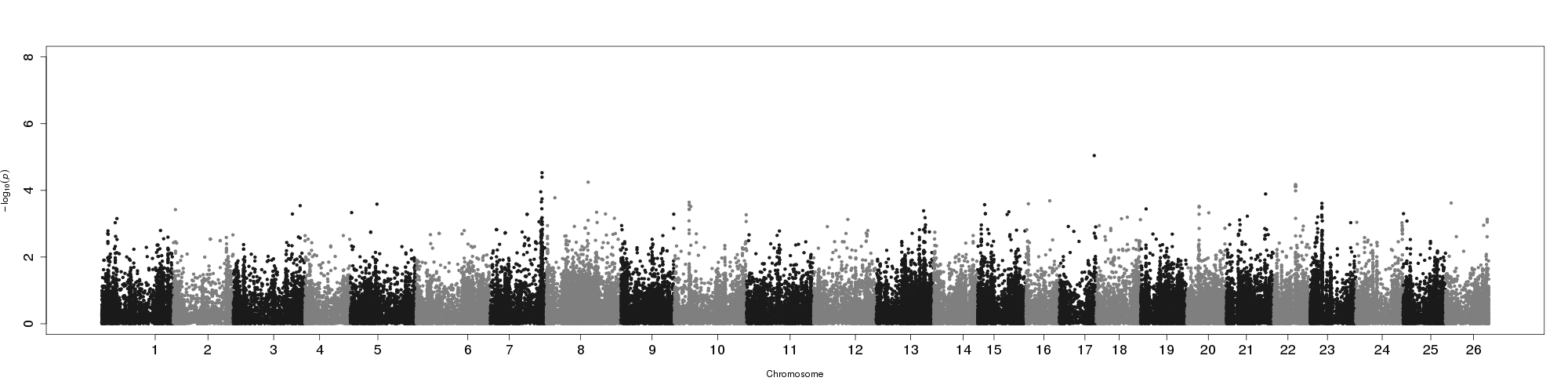

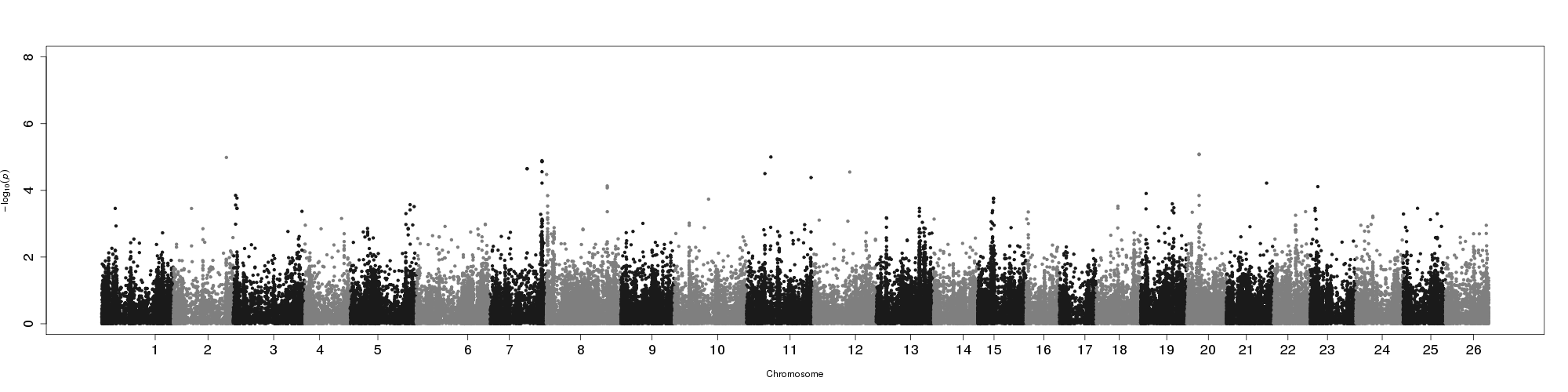

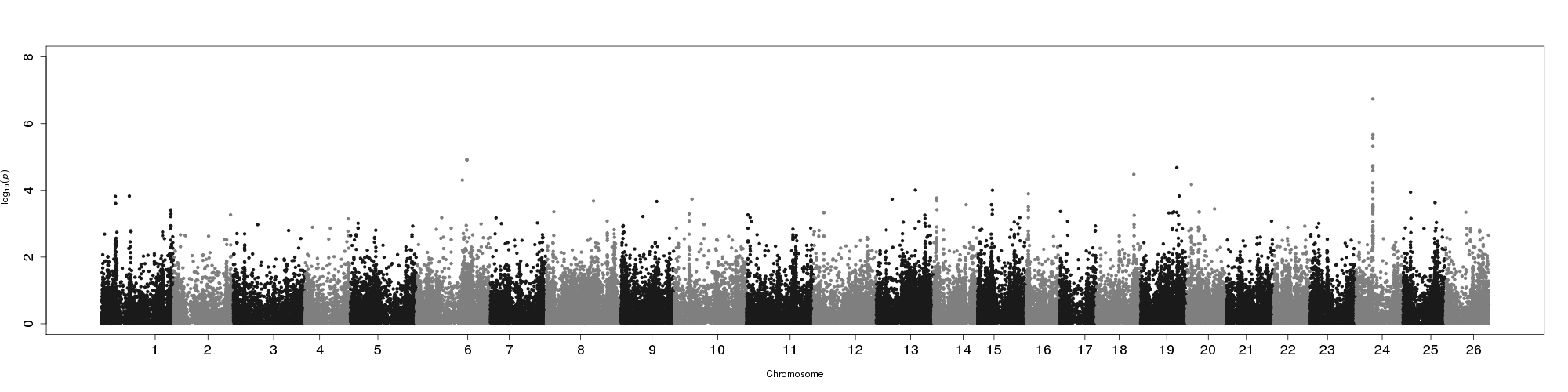


Fig. S1: The Manhattan block of BW under nine environments.


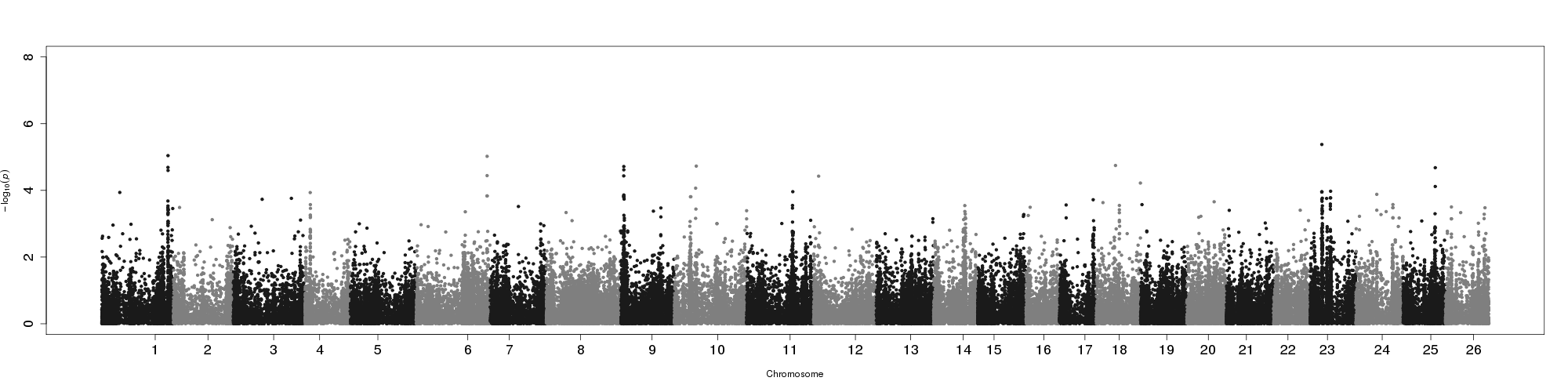

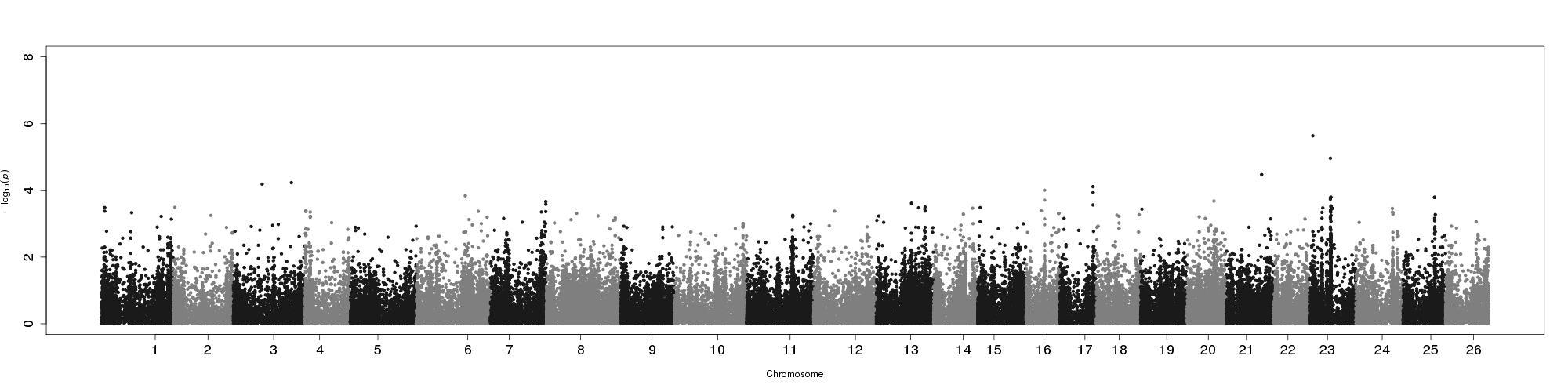

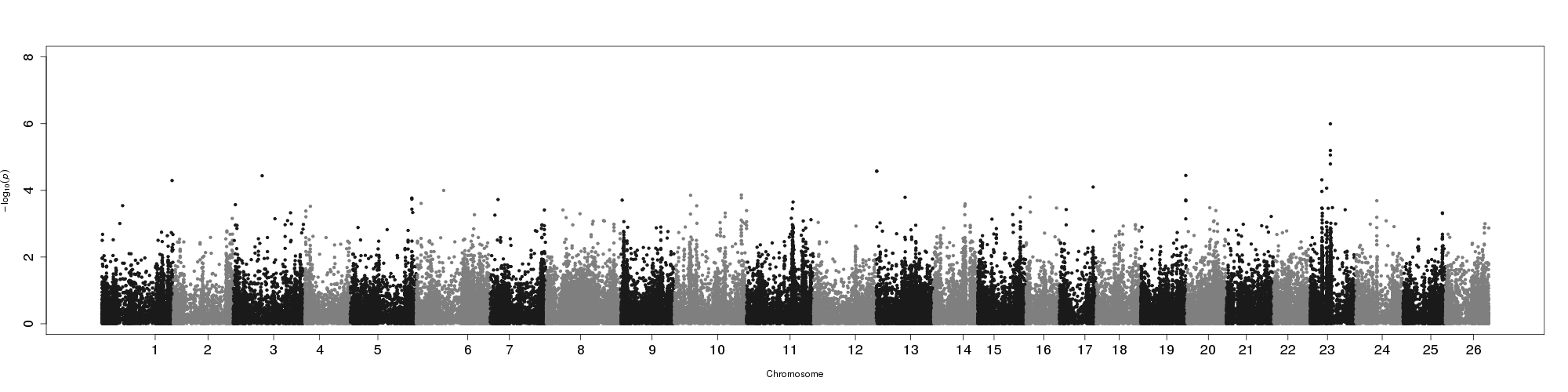

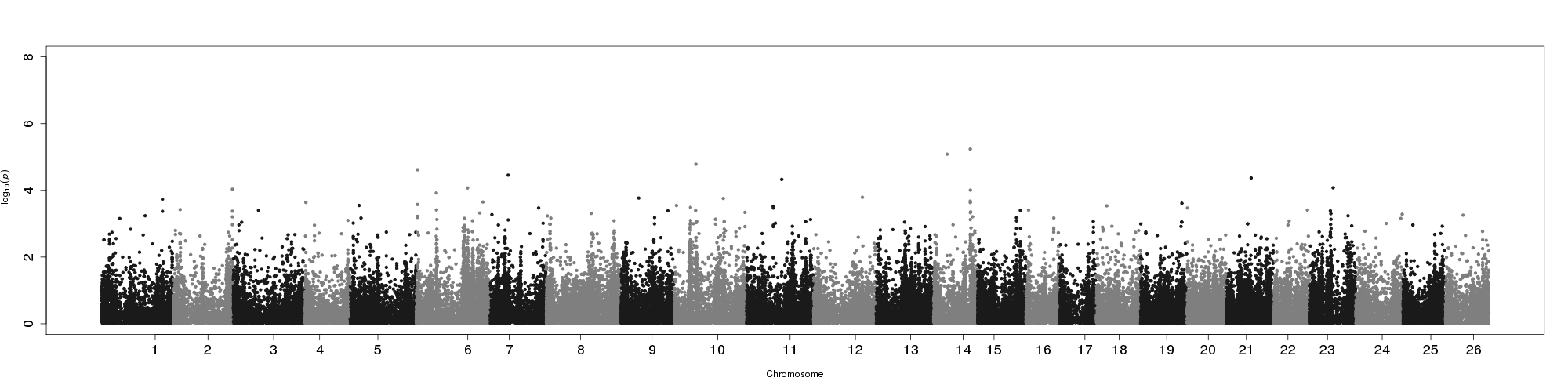

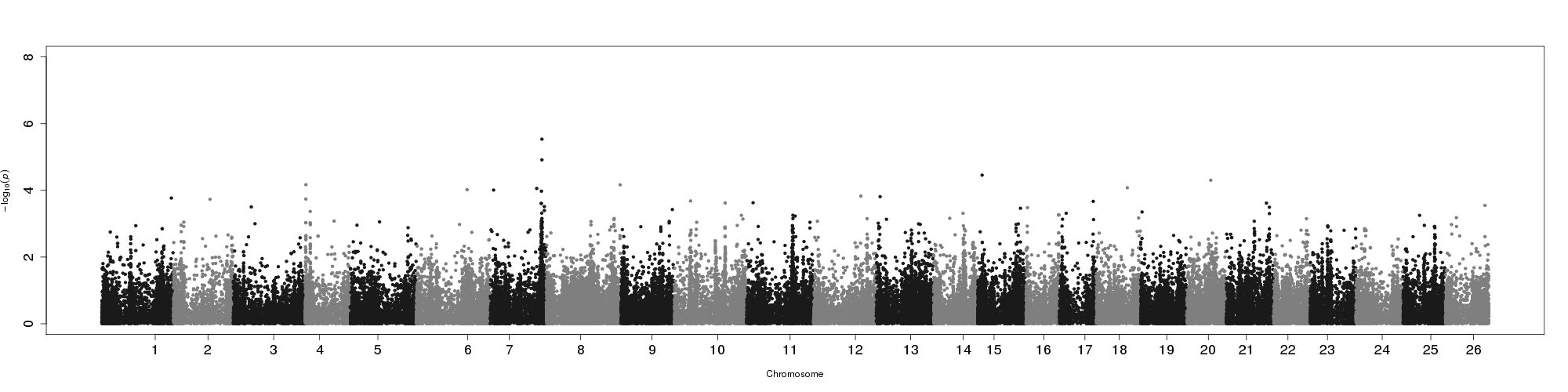

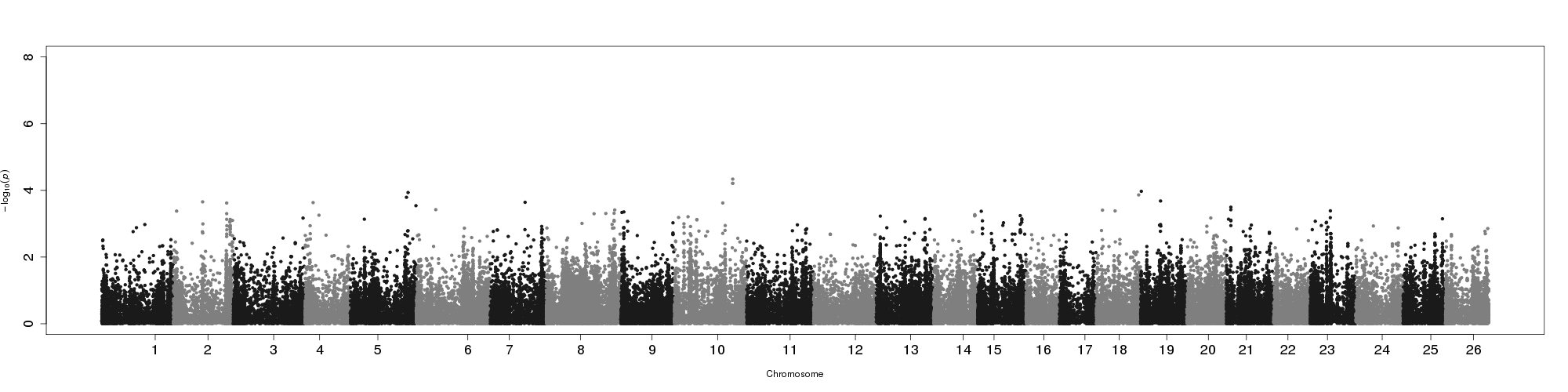

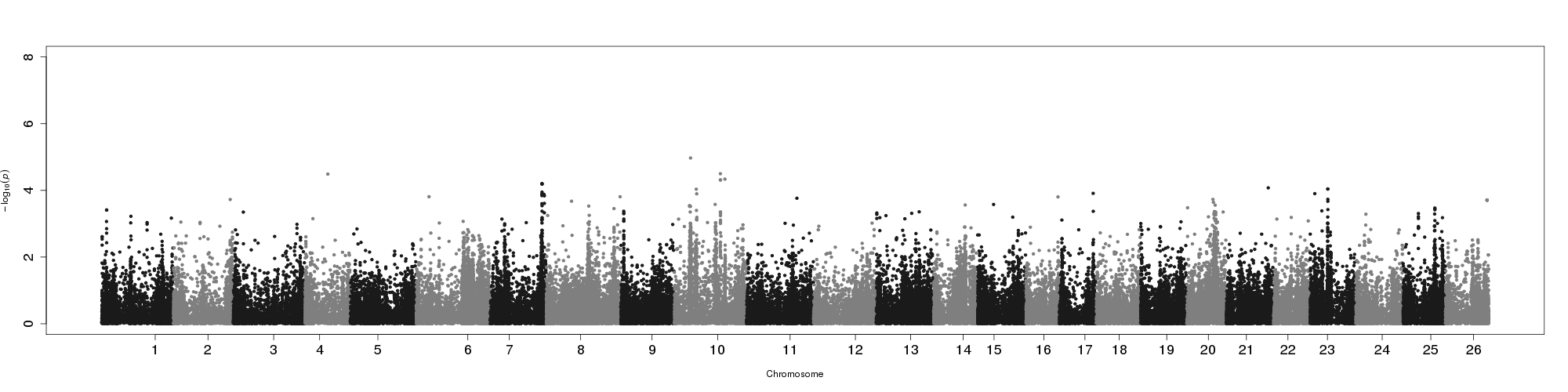

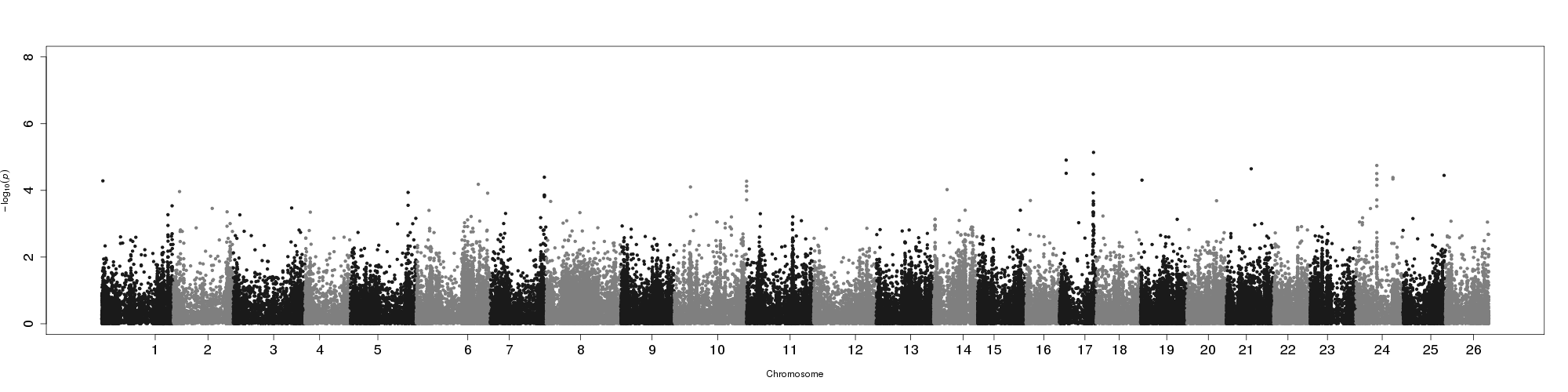

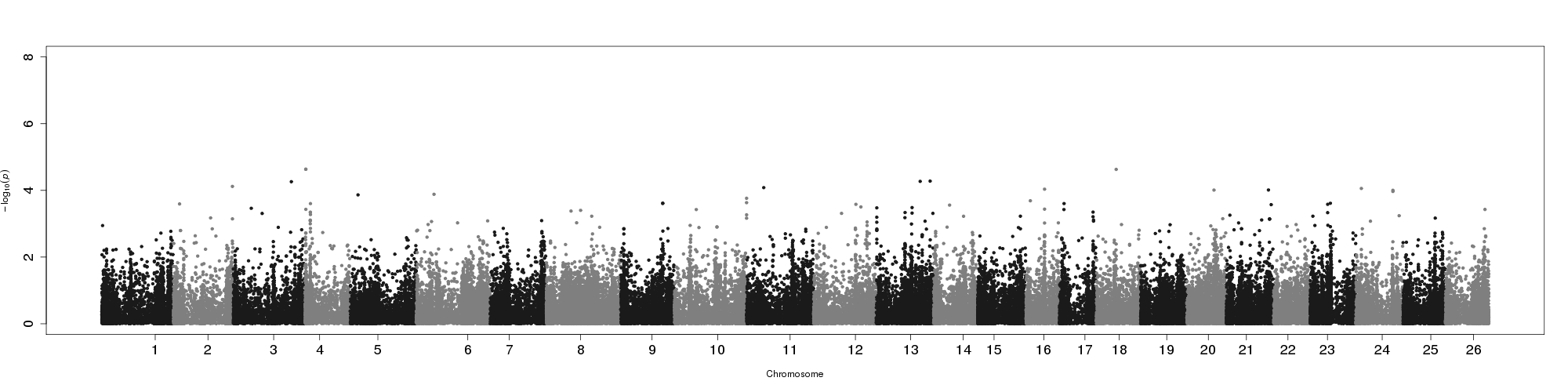
 Fig. S2: The Manhattan block of FE under nine environments.


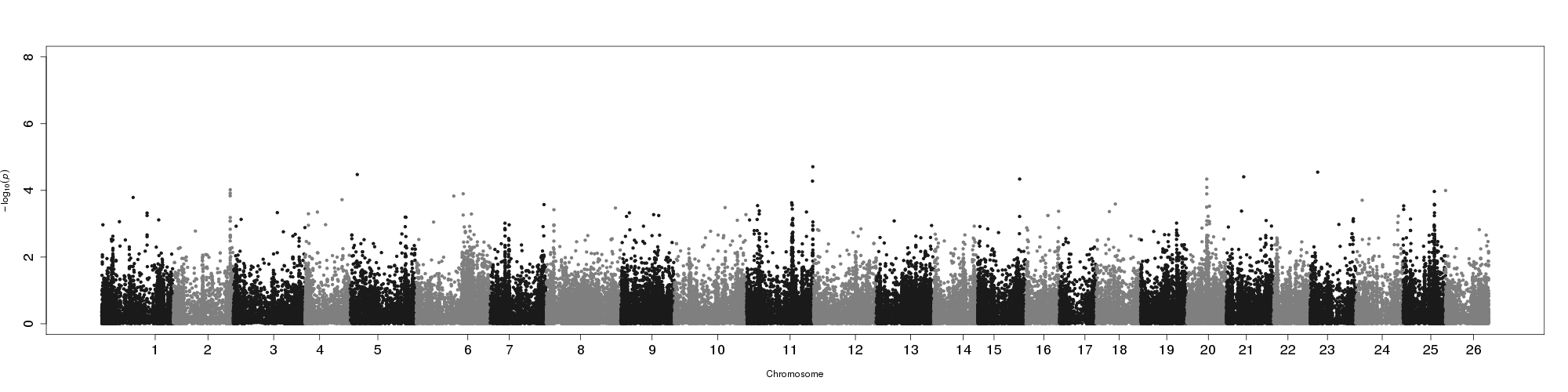


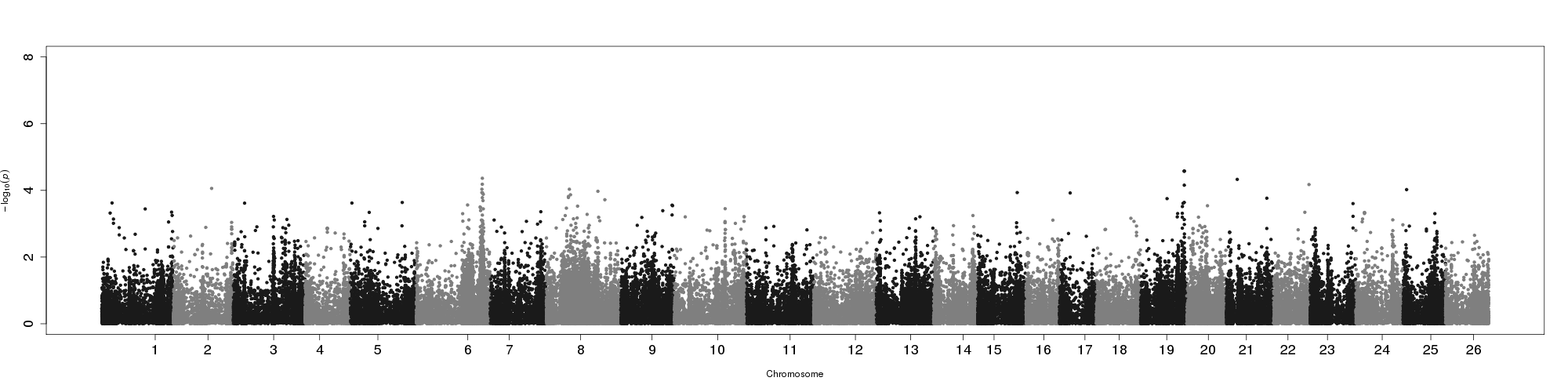

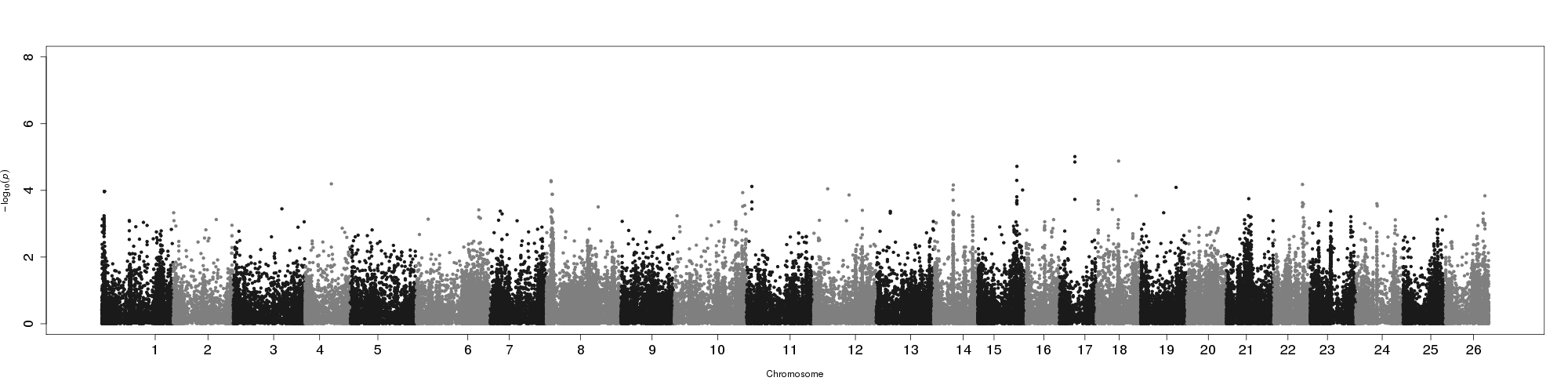

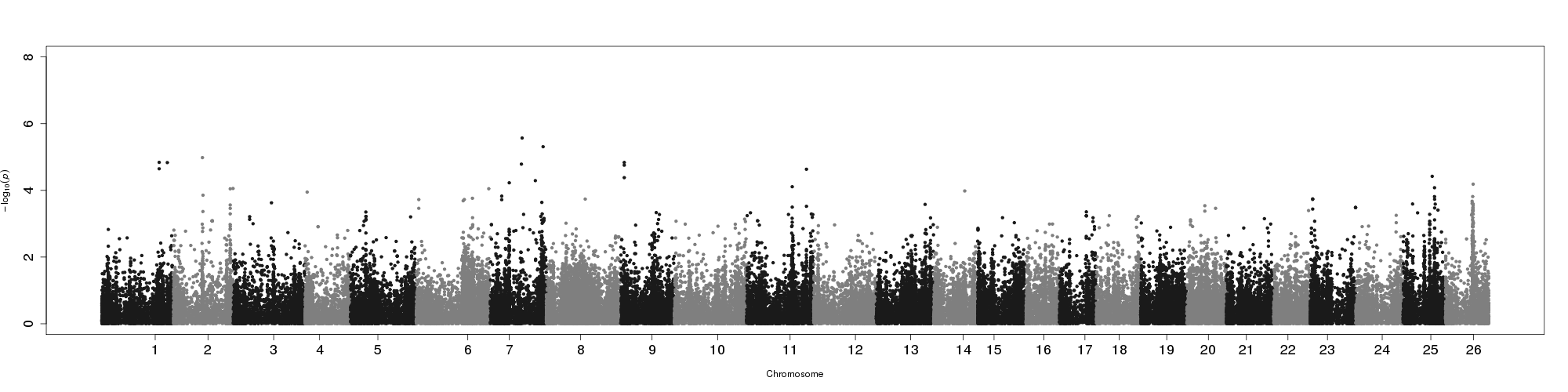

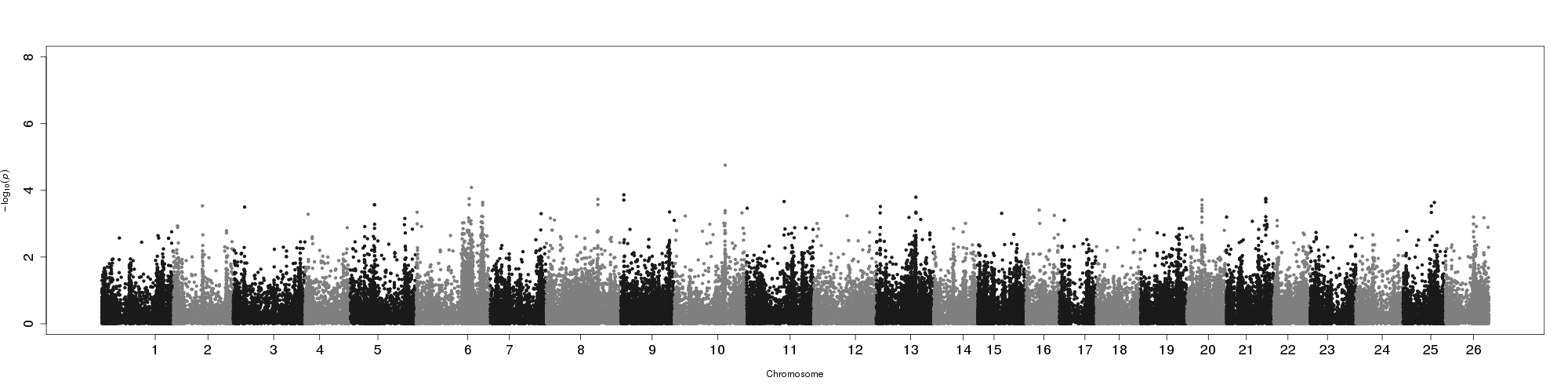

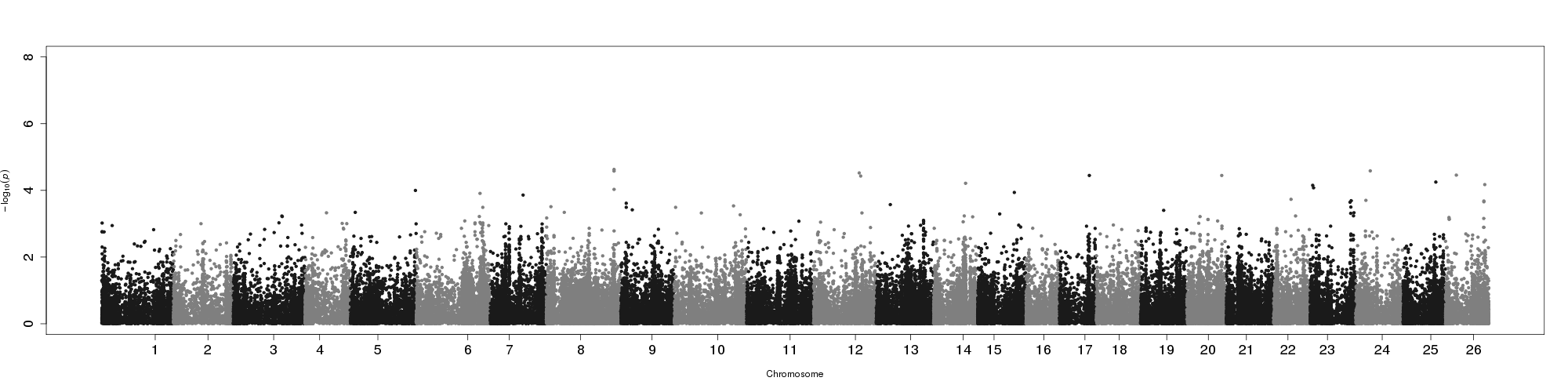

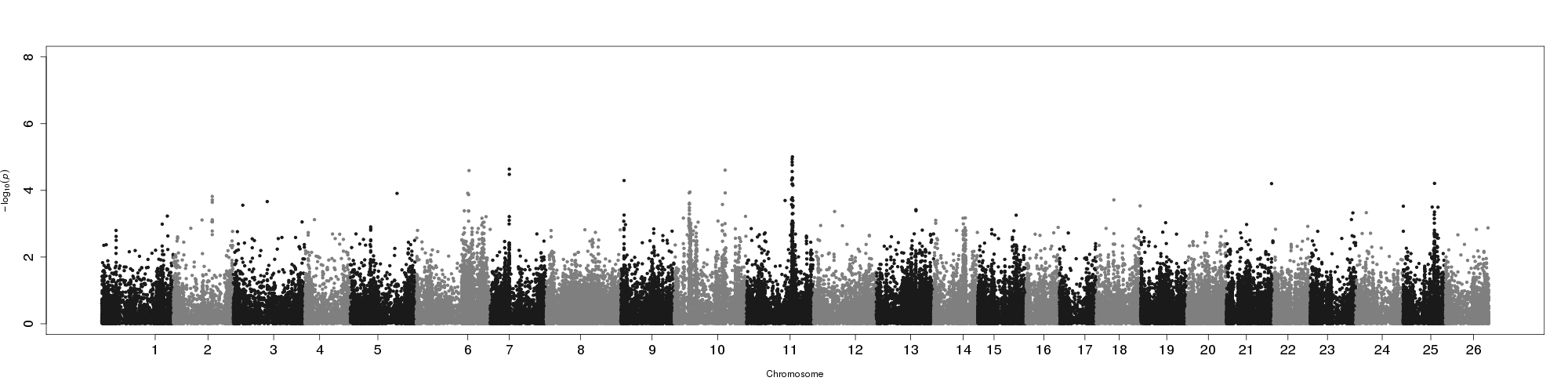

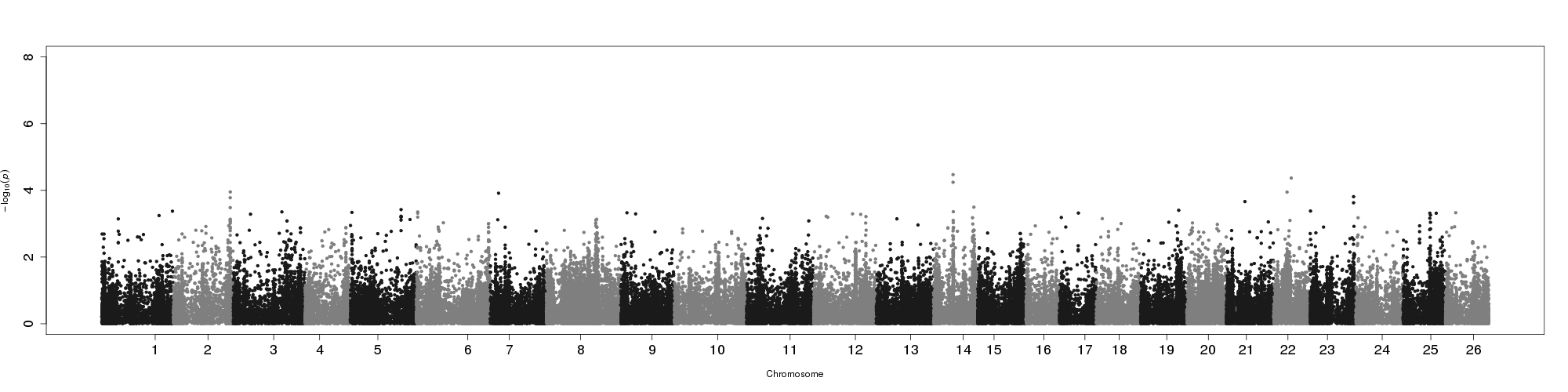
 Fig. S3: The Manhattan block of FD under nine environments.


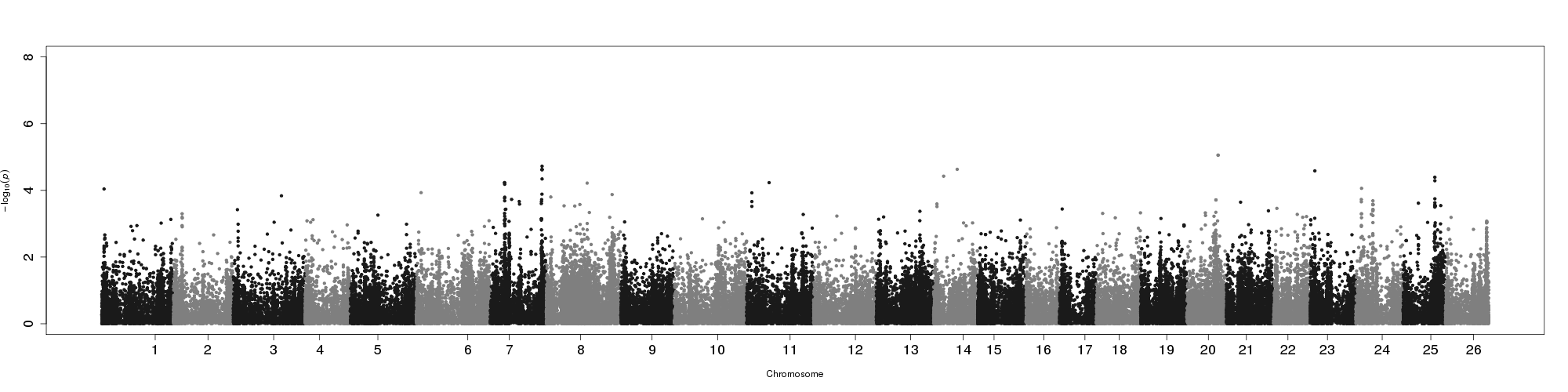

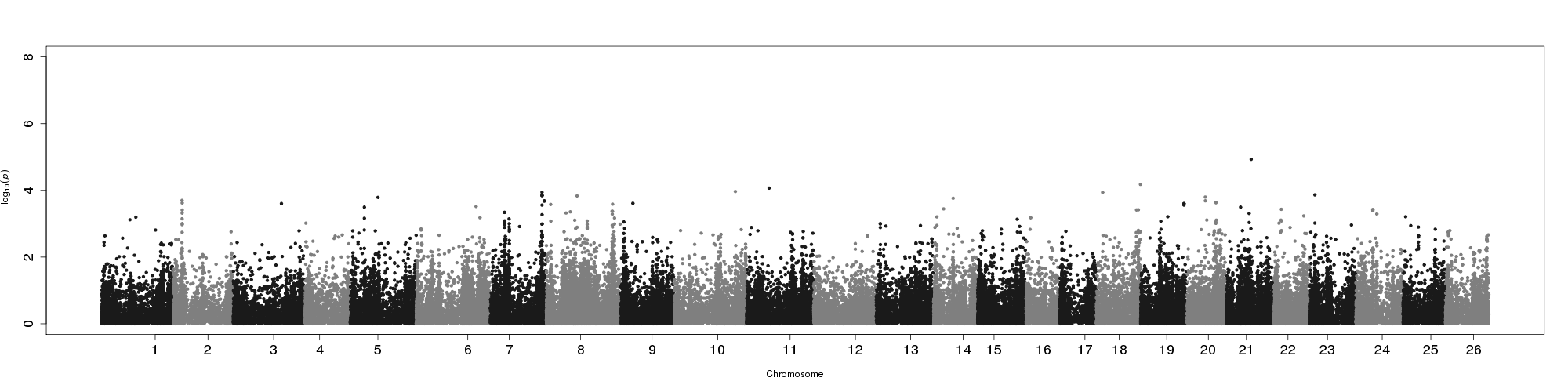

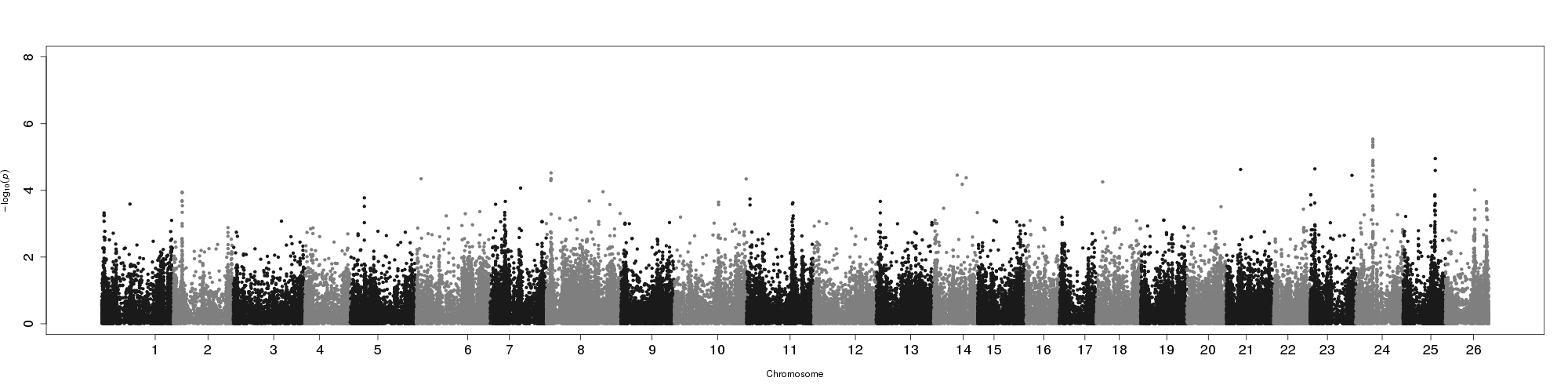

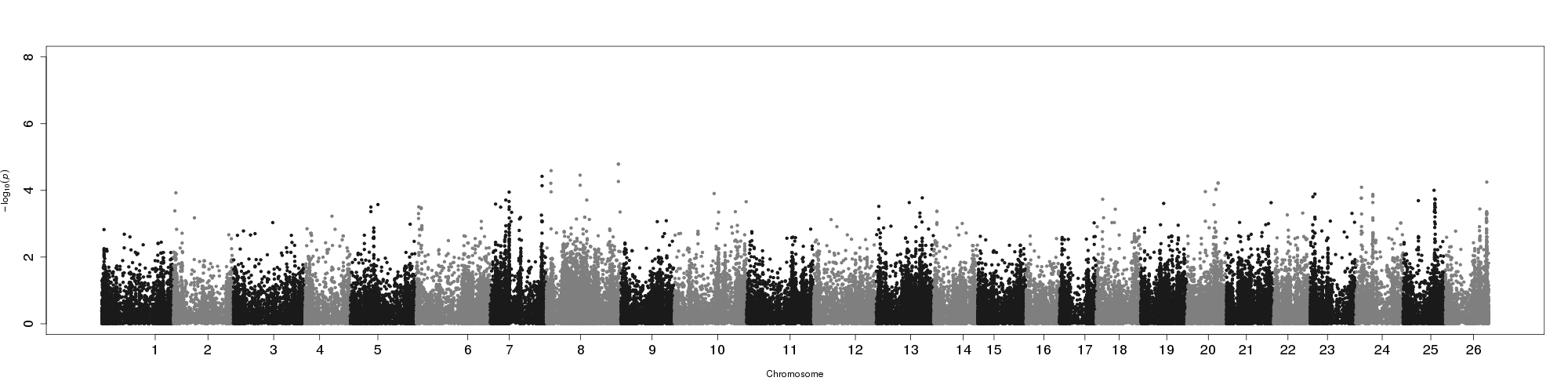

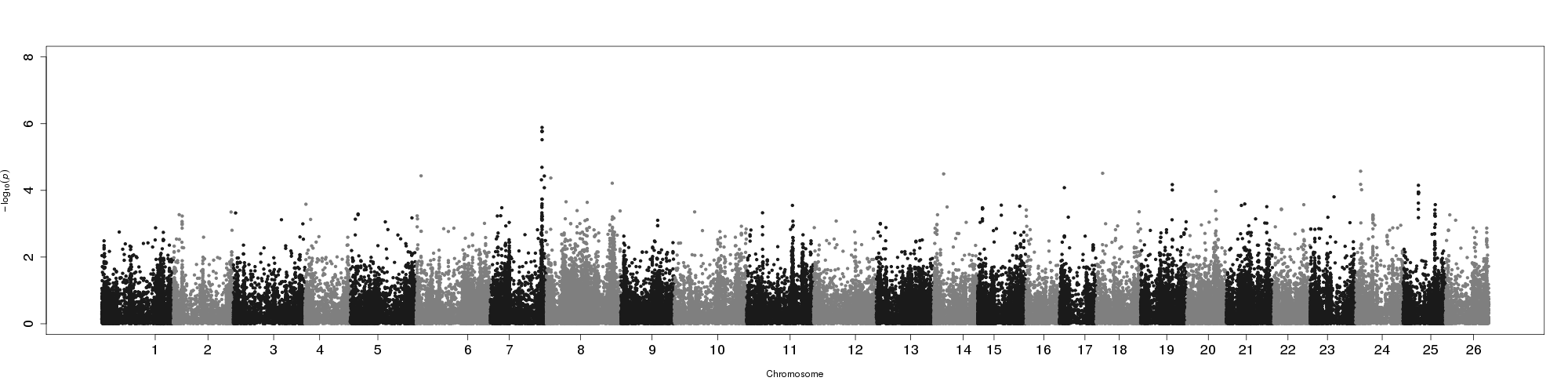

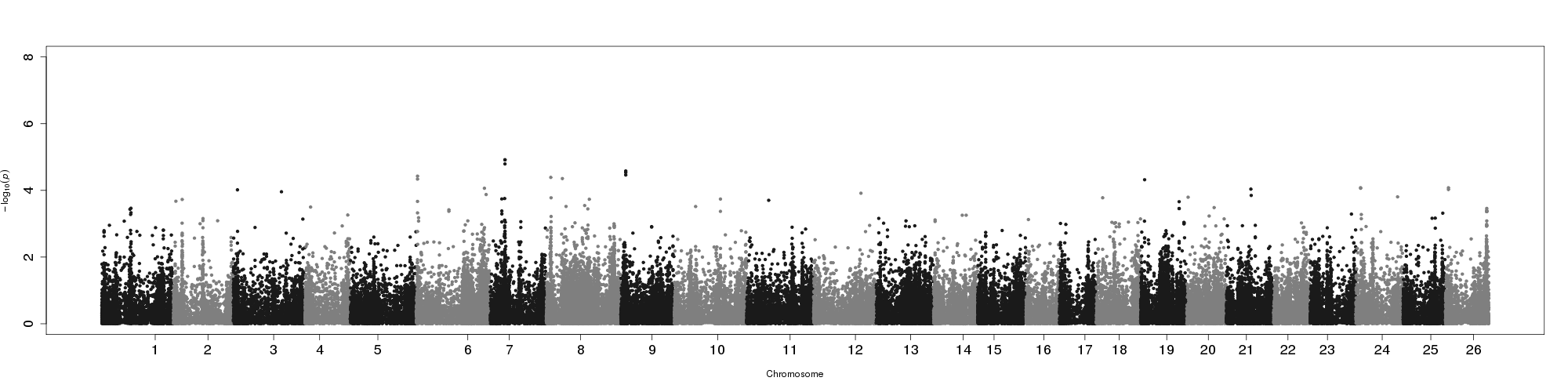

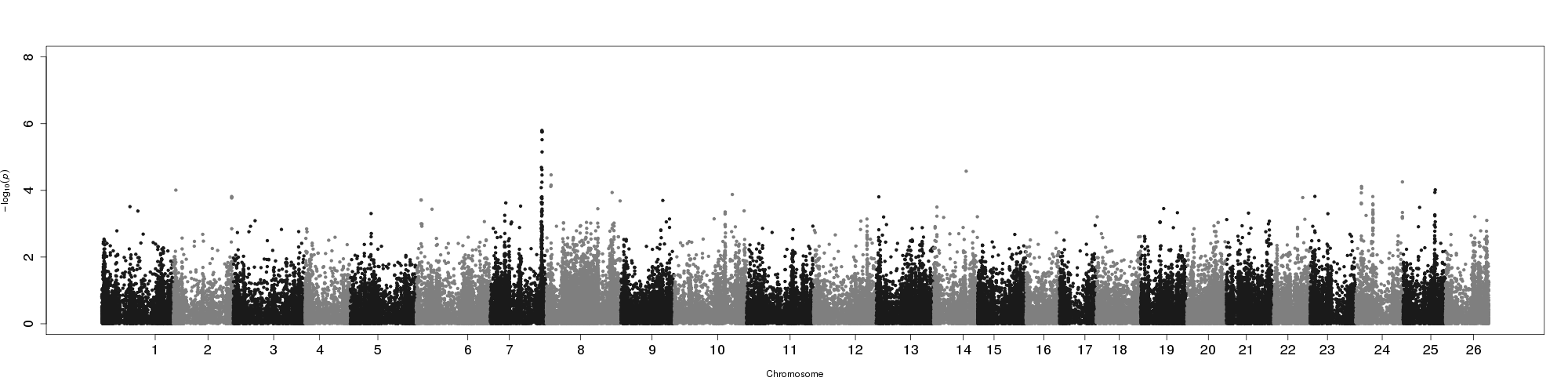

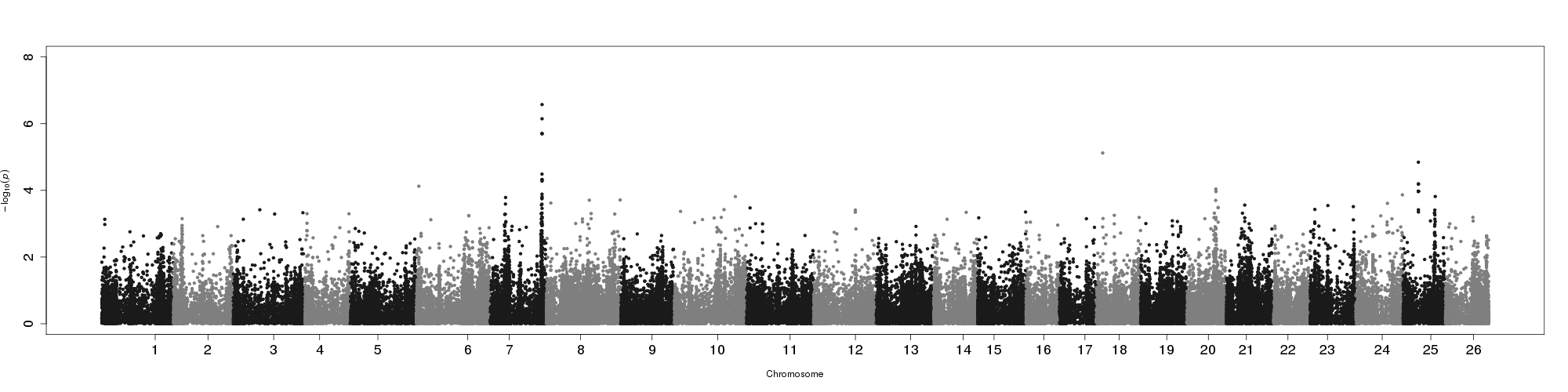

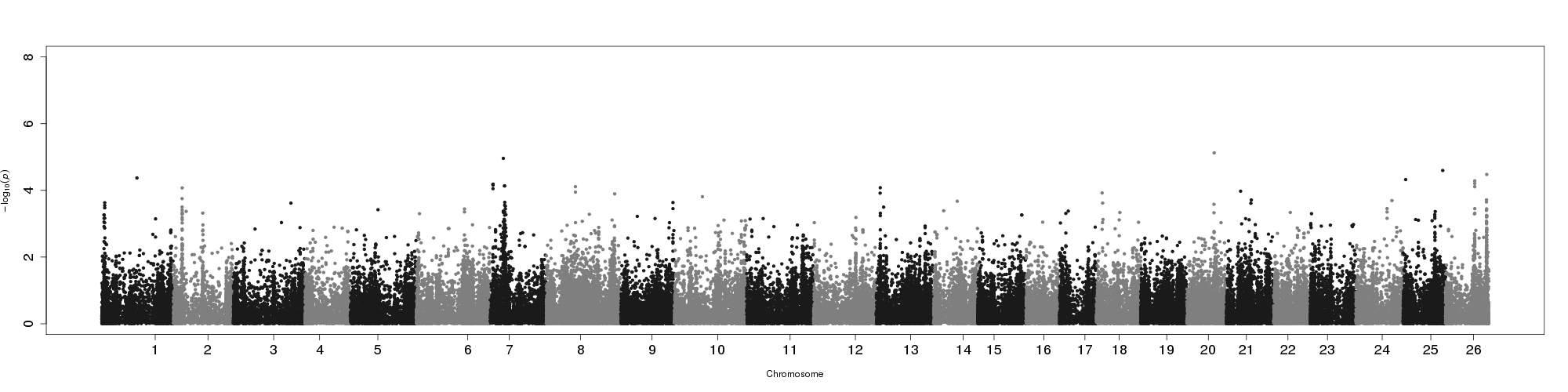
 Fig. S4: The Manhattan block of FL under nine environments.


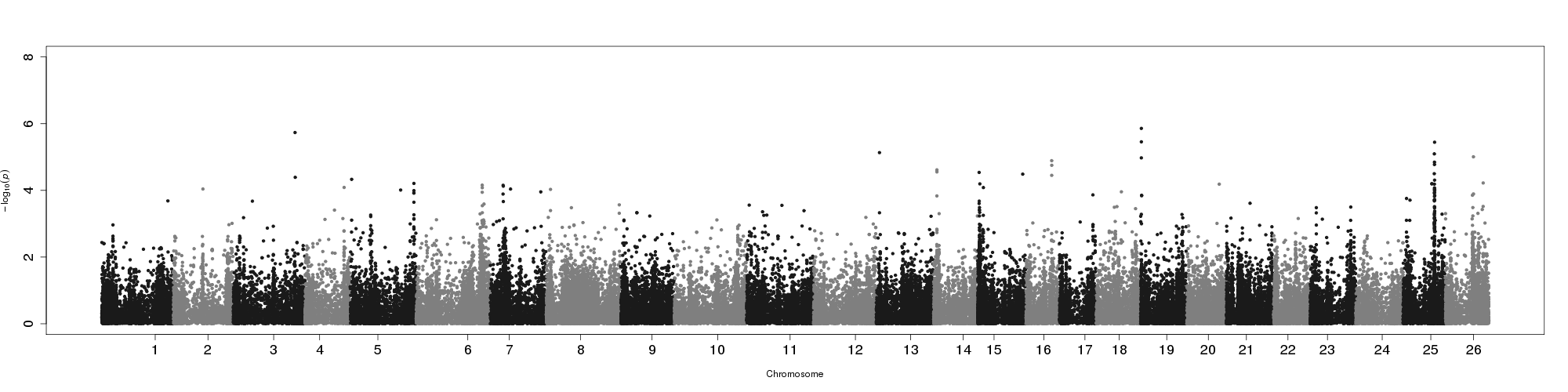

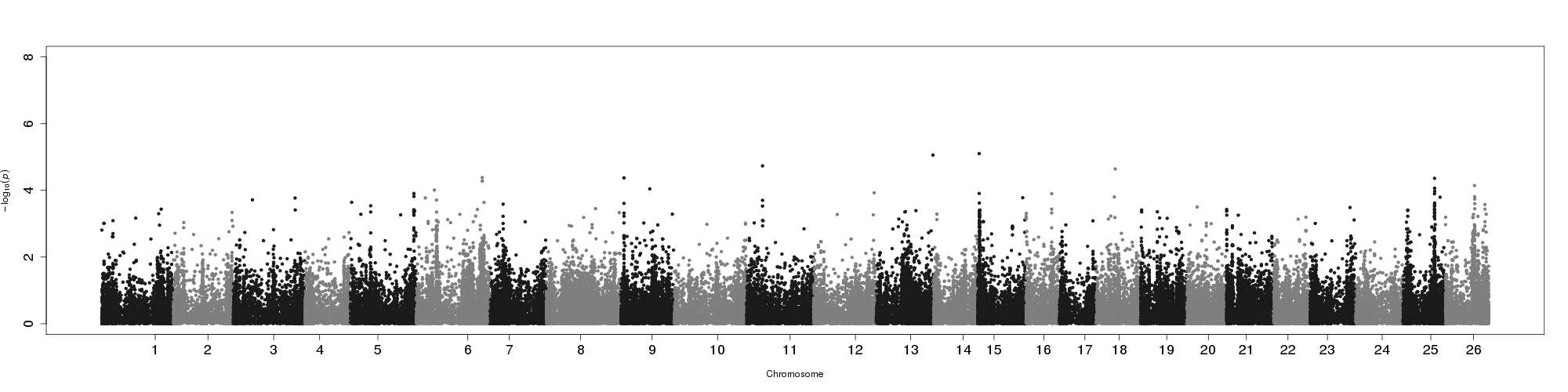

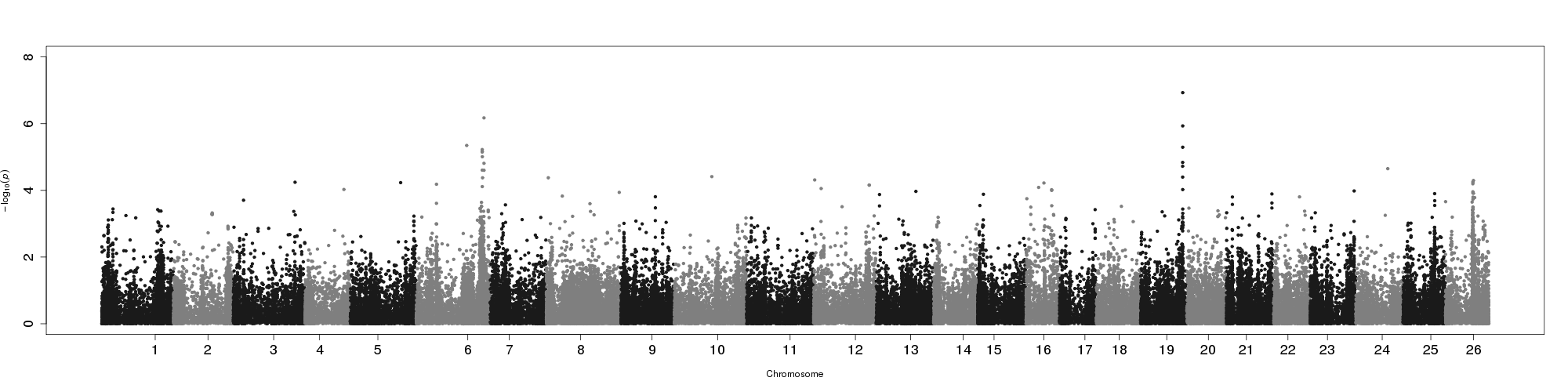

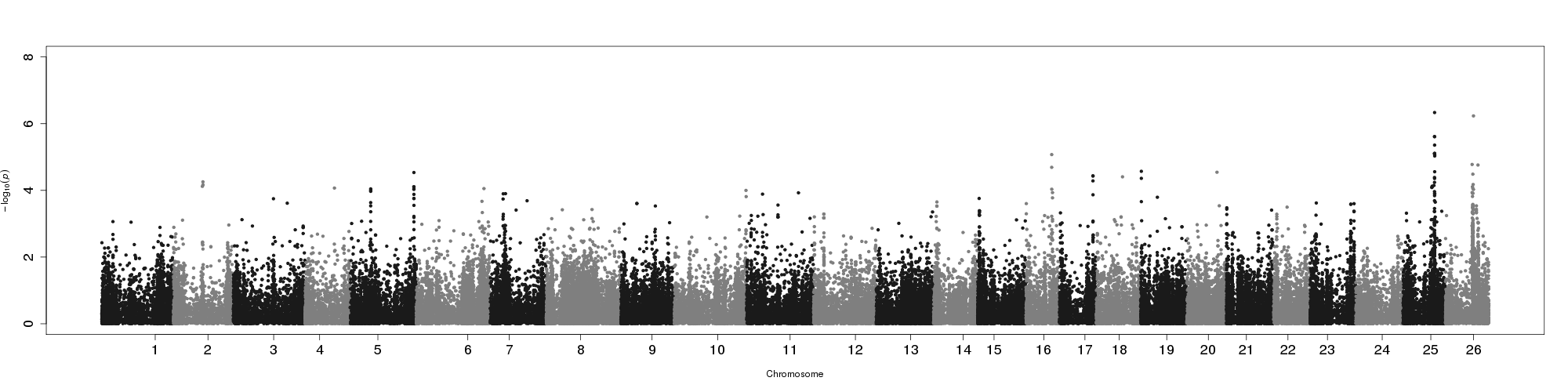

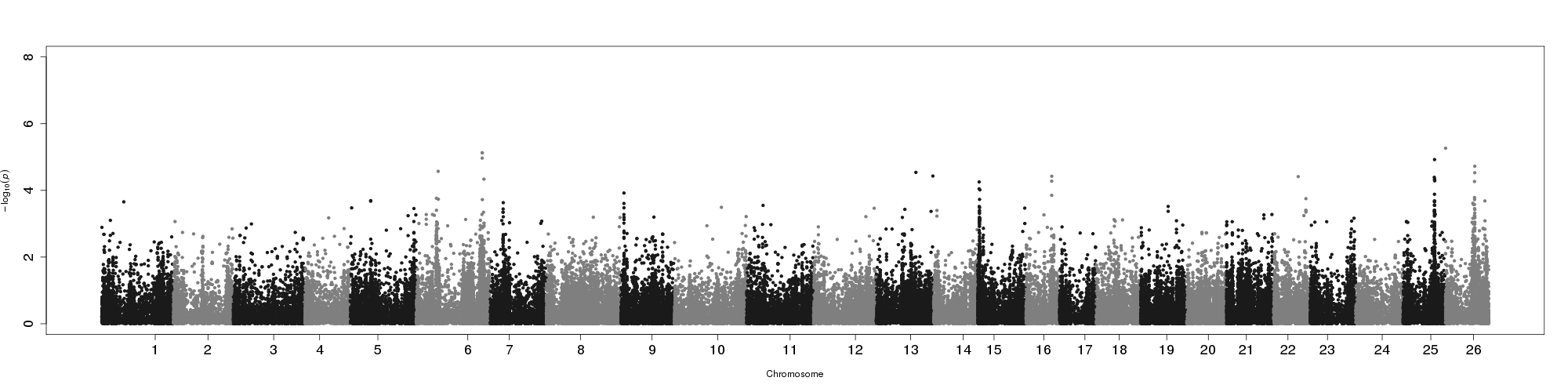

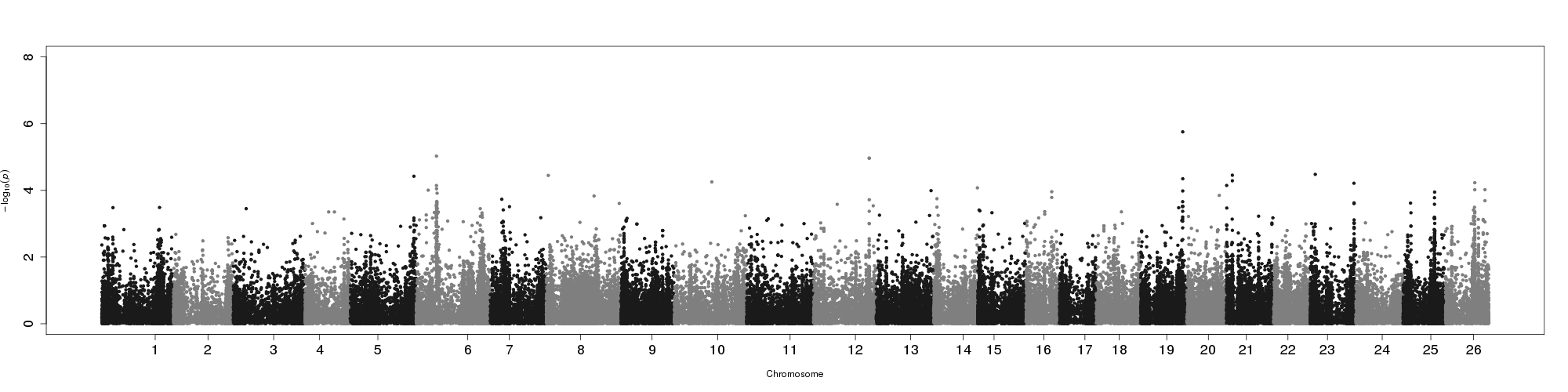

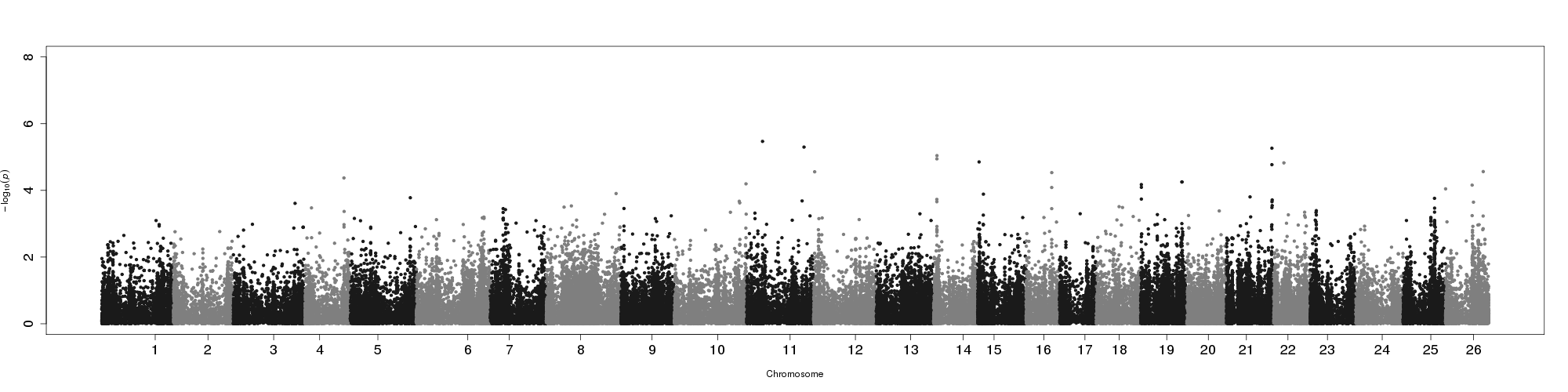

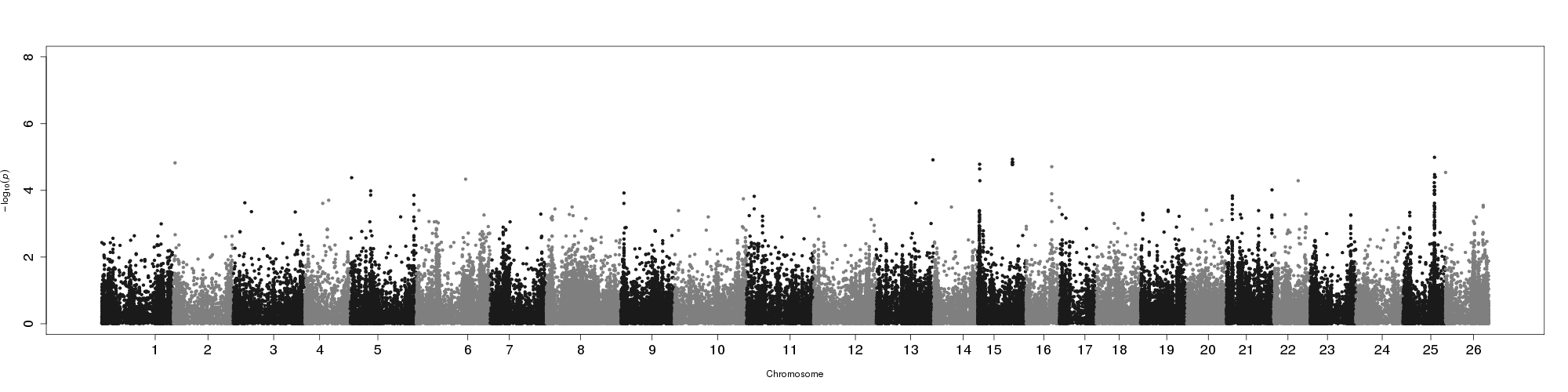

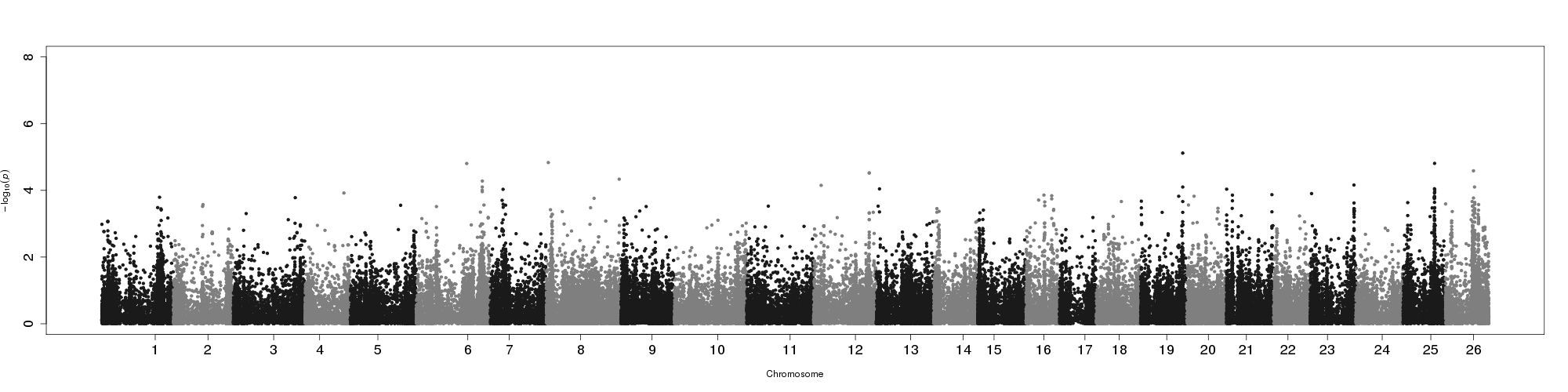
 Fig. S5: The Manhattan block of LP under nine environments.


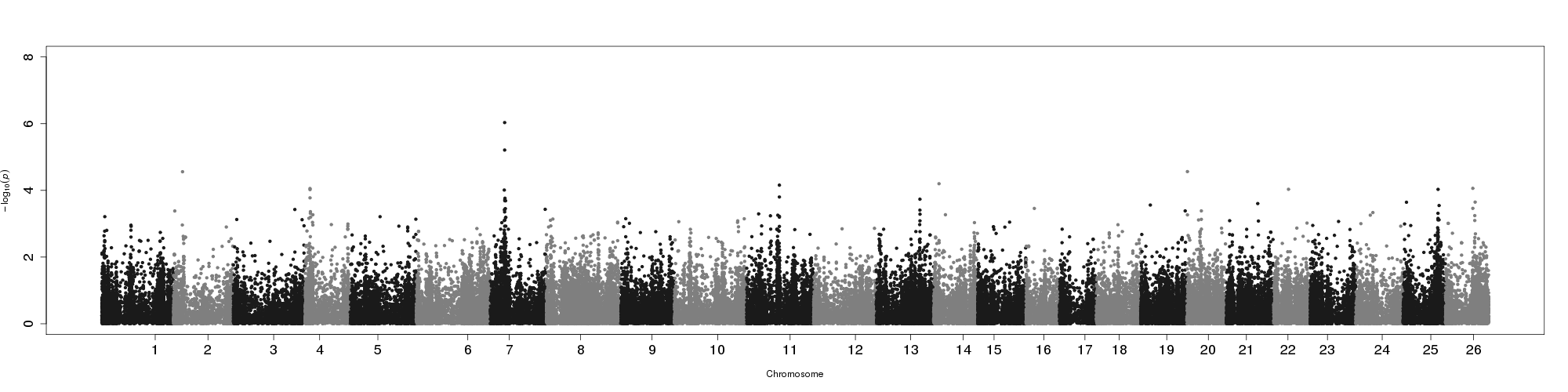

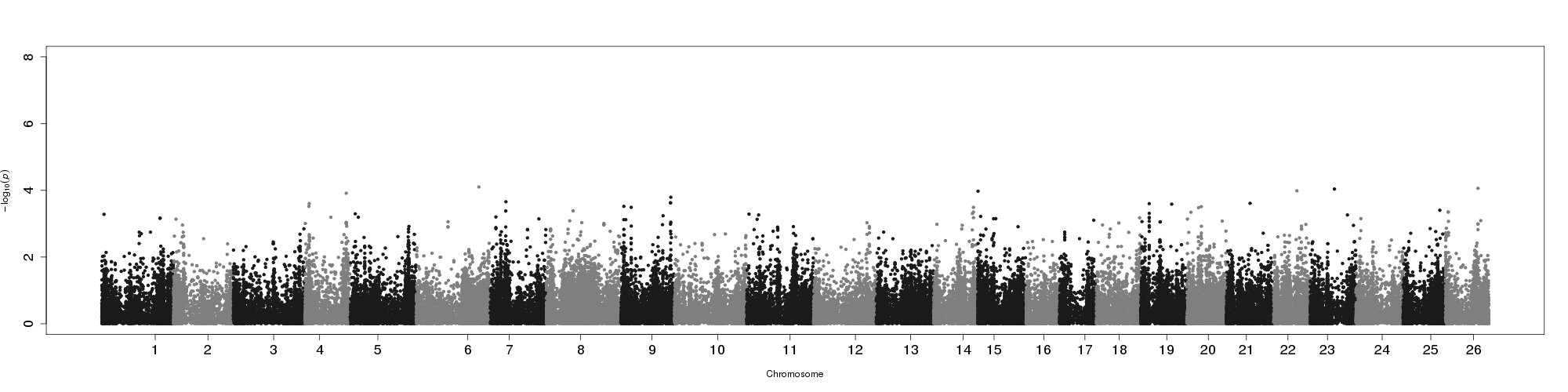

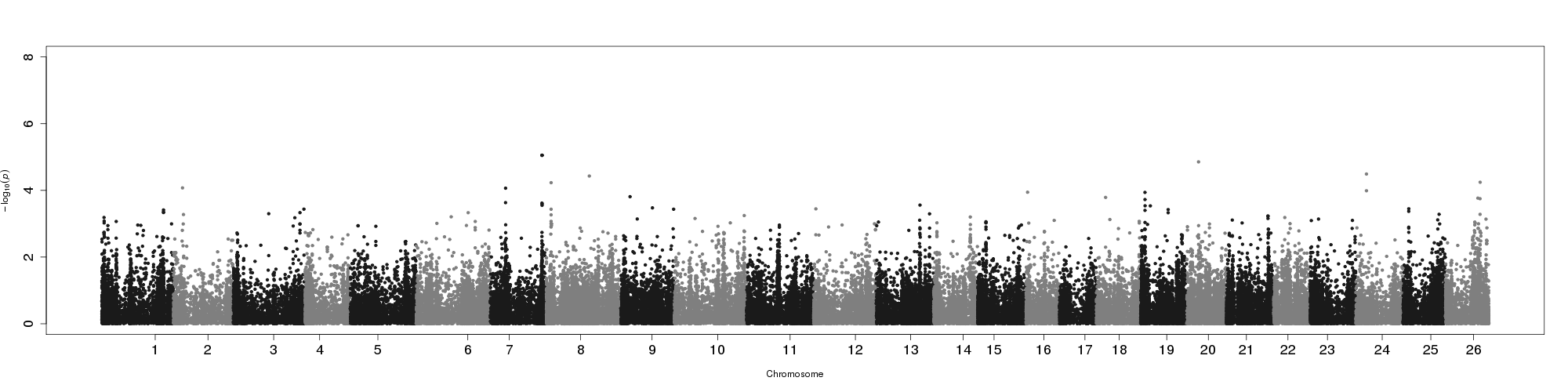

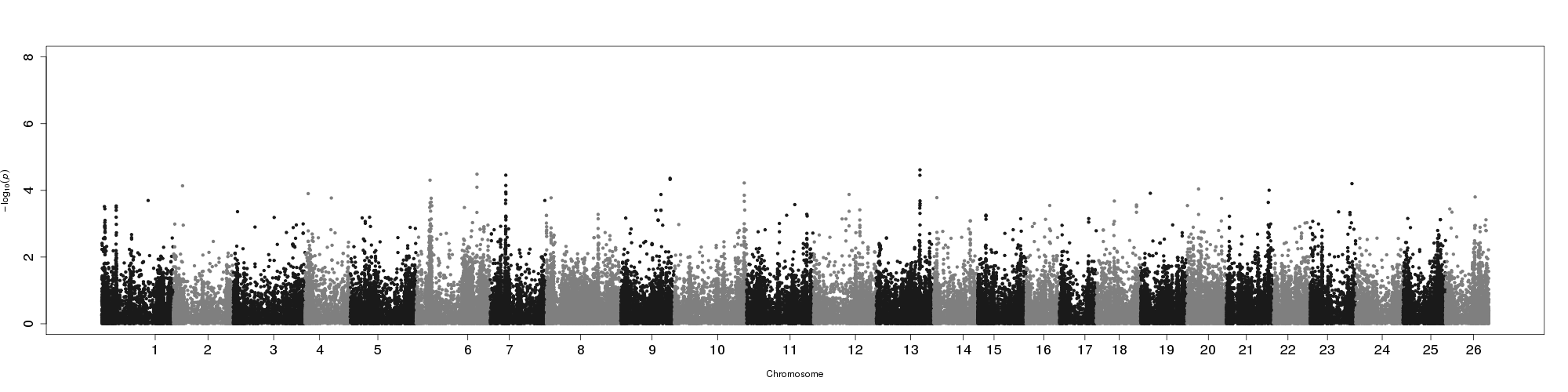

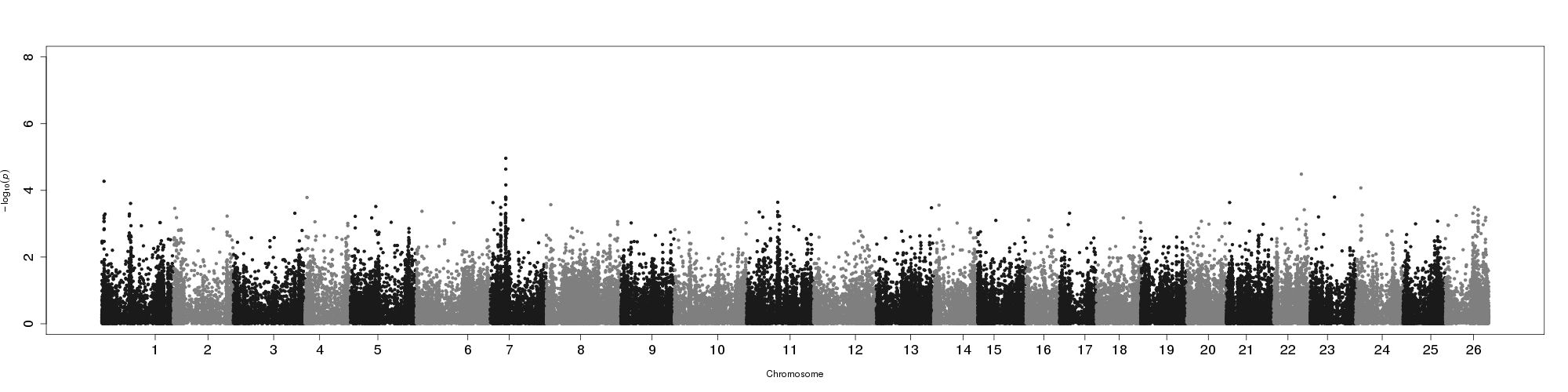

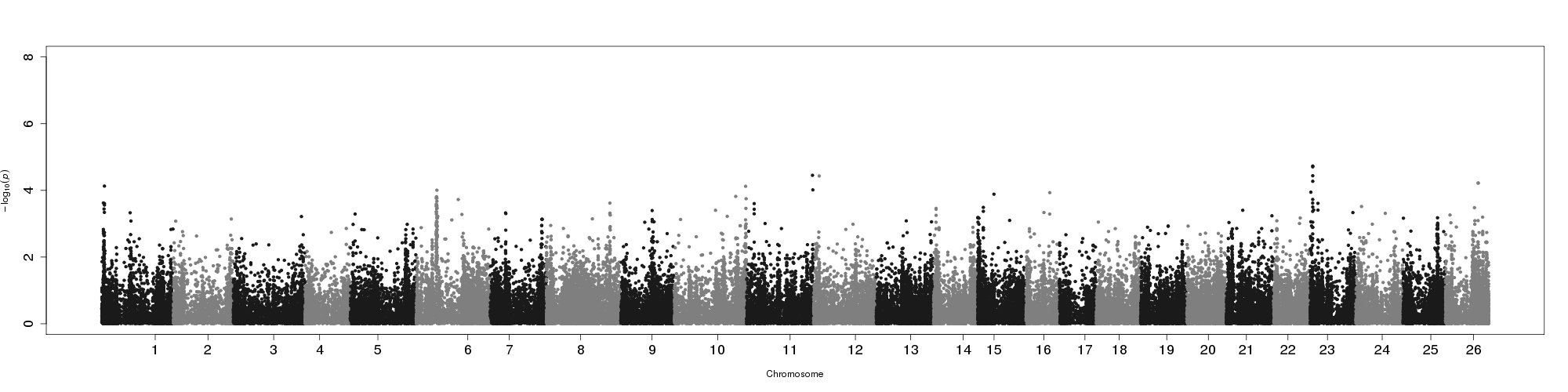

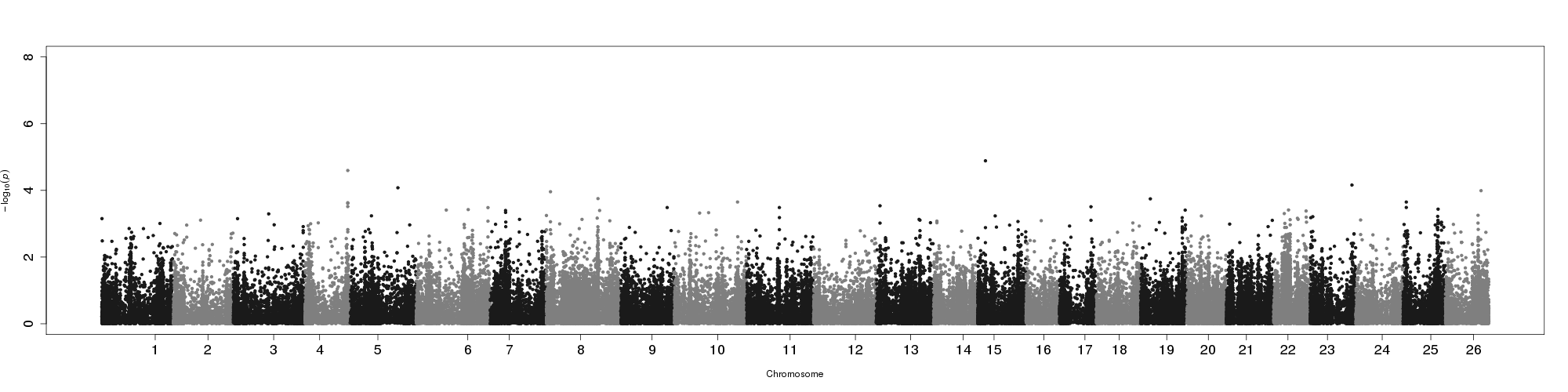

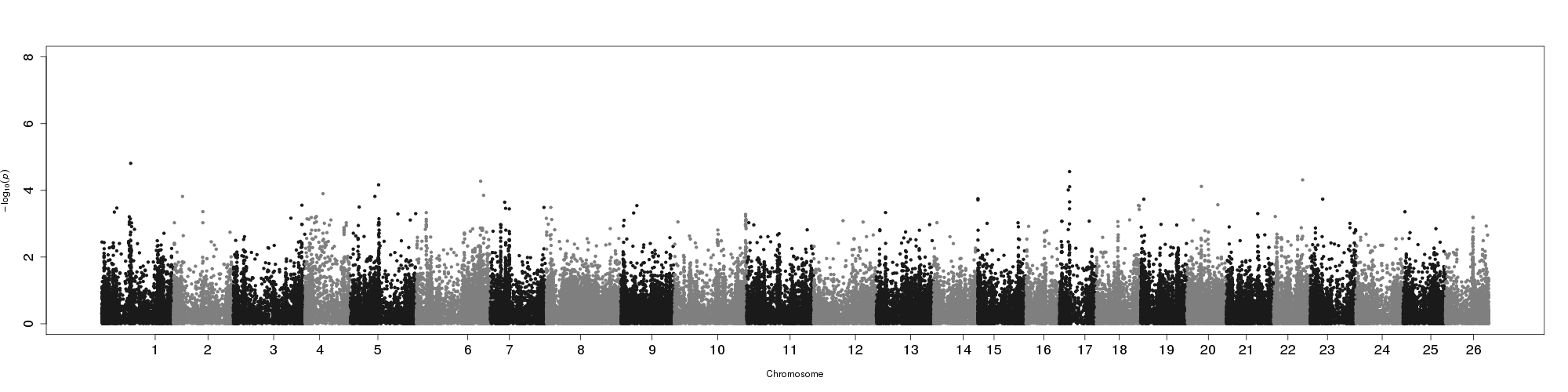

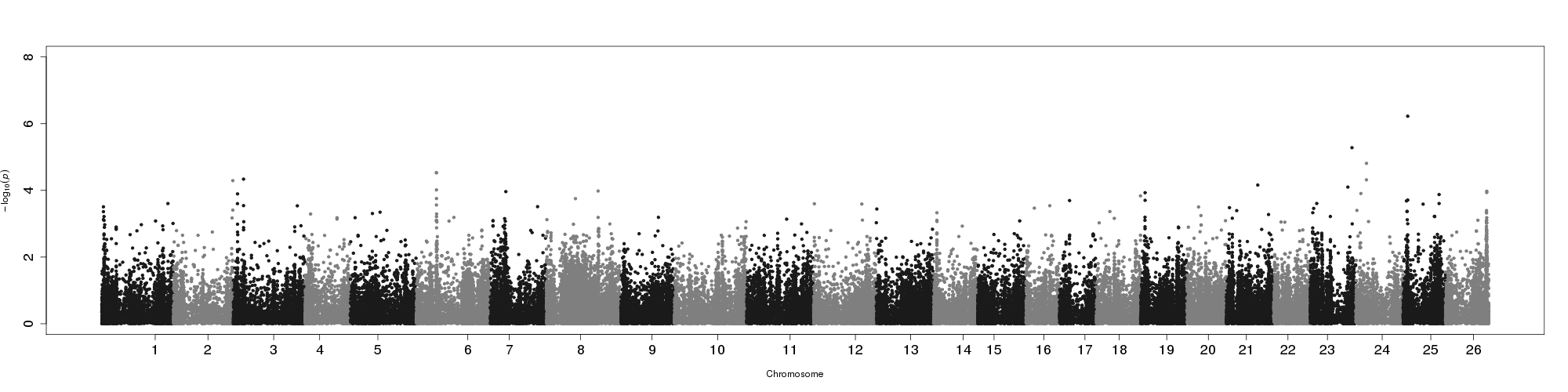
 Fig. S6: The Manhattan block of FM under nine environments.


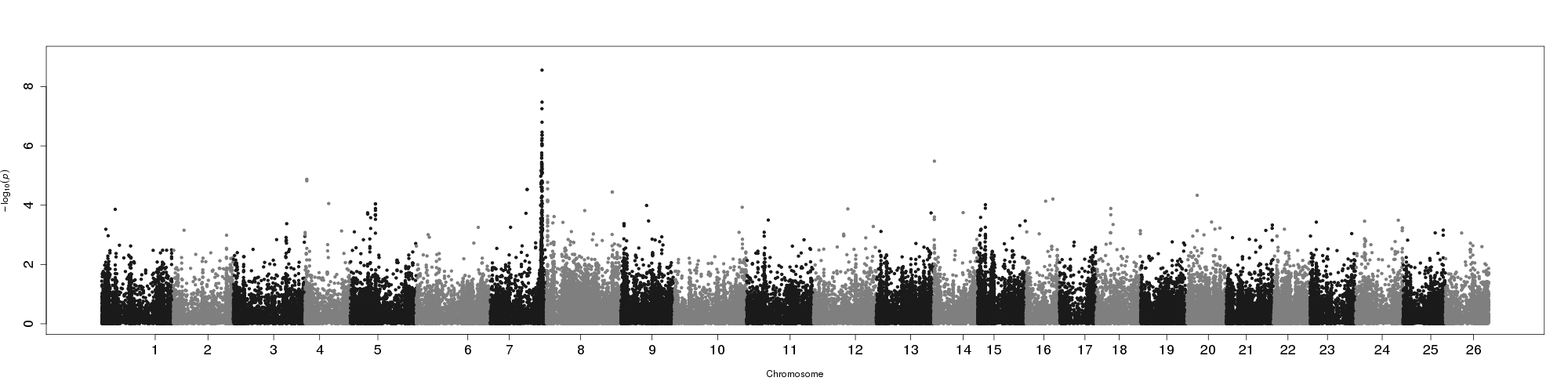

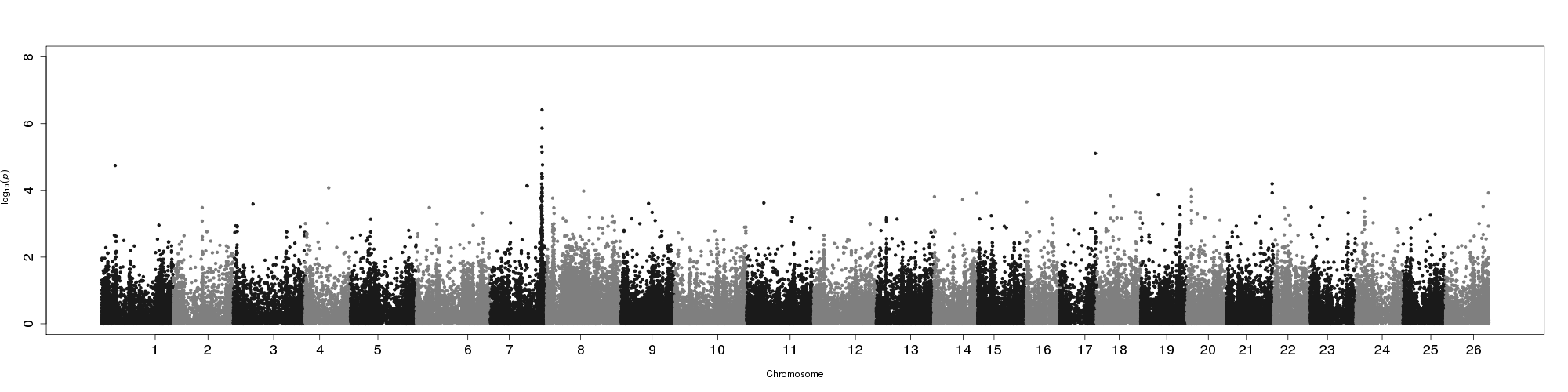

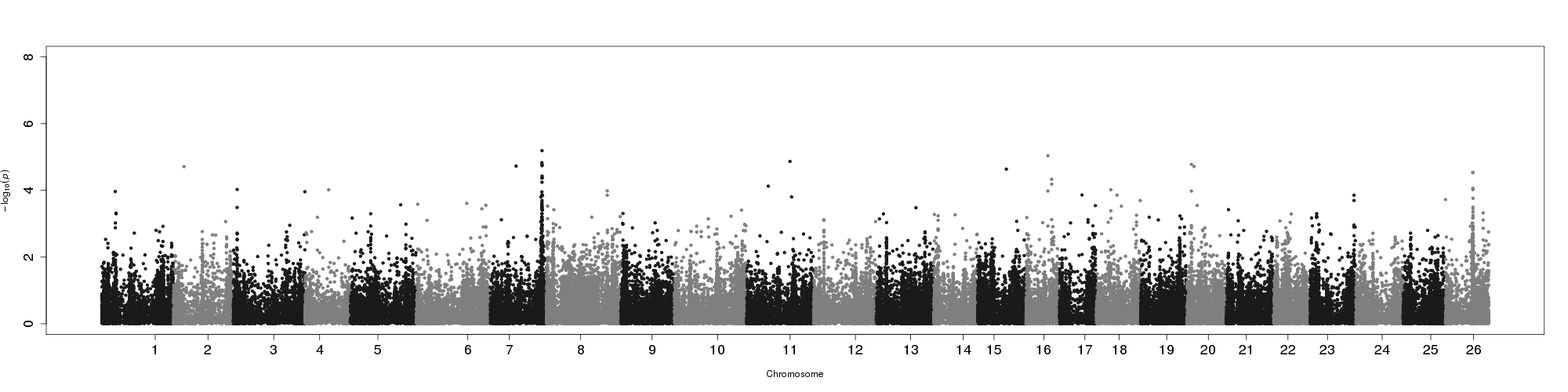

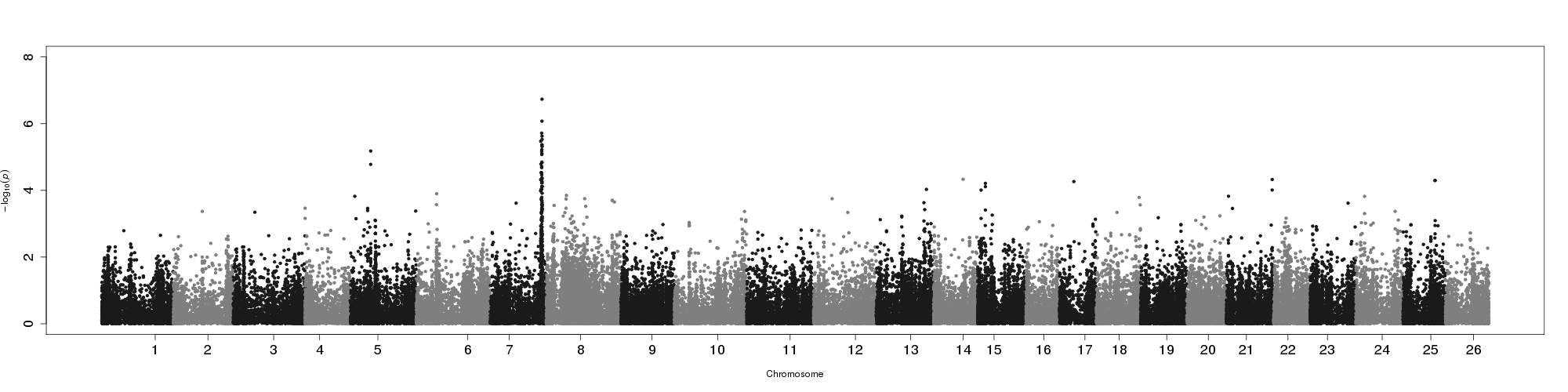

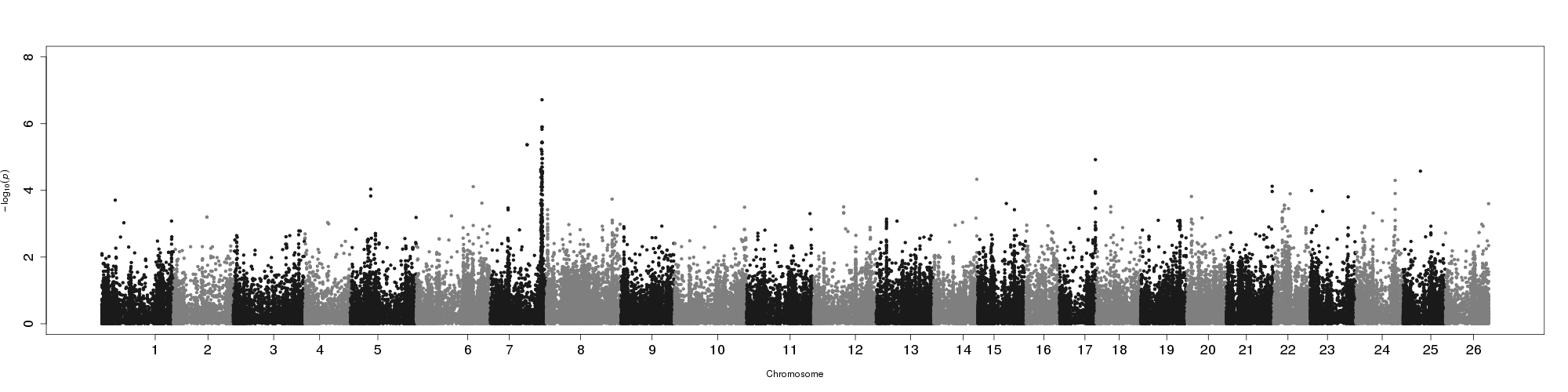

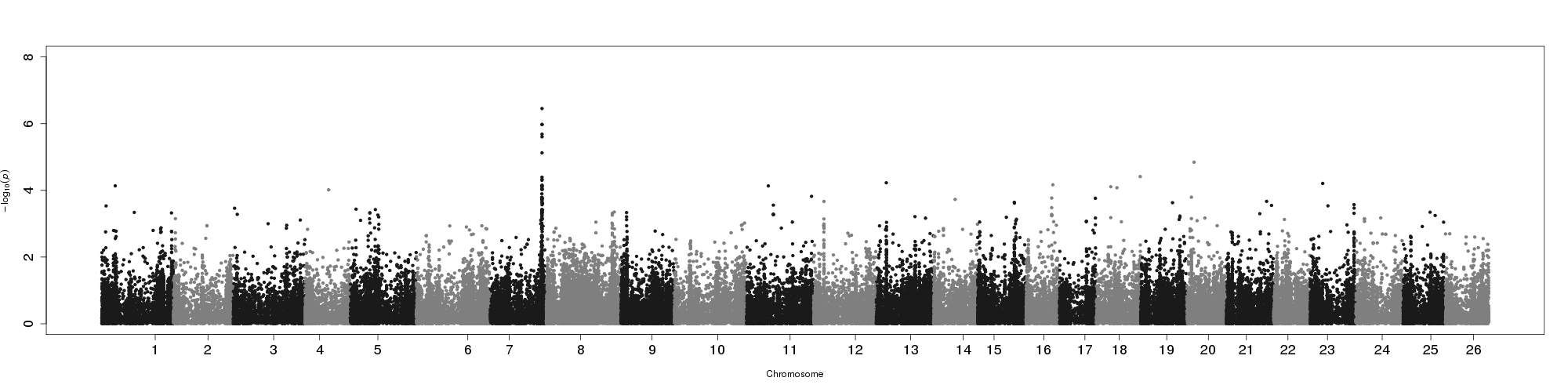

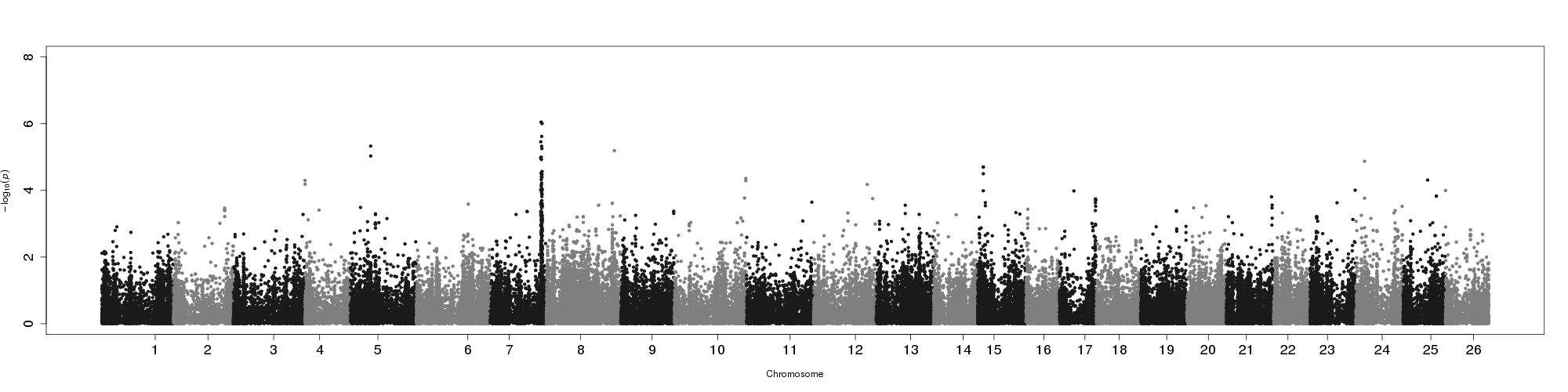

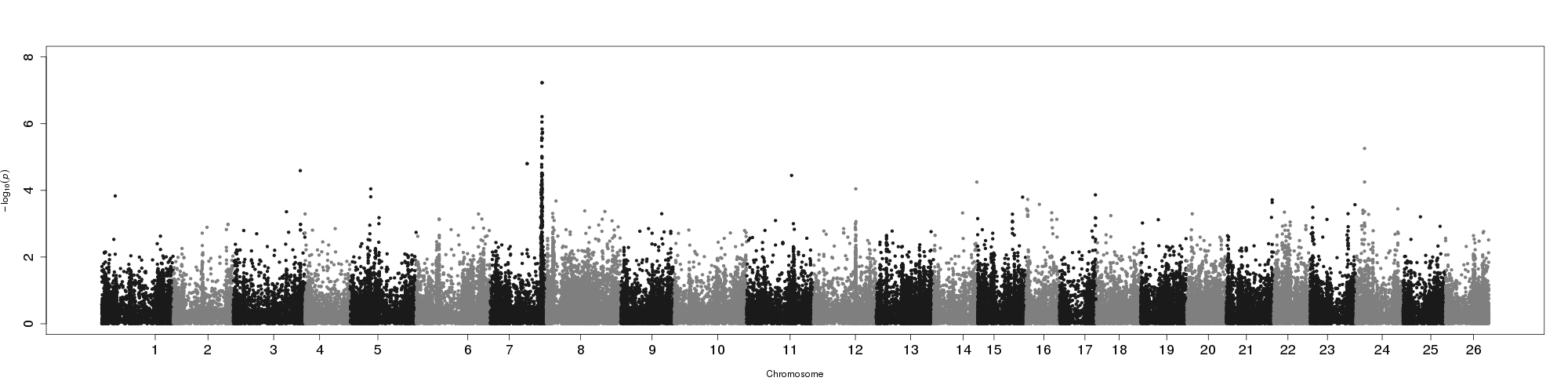

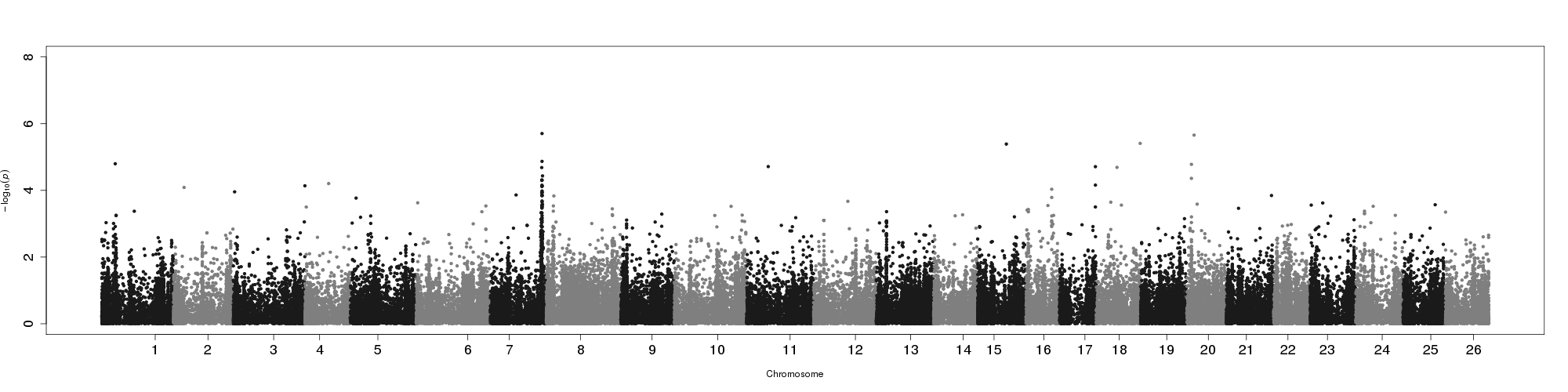


Fig. S7: The Manhattan block of SI under nine environments.


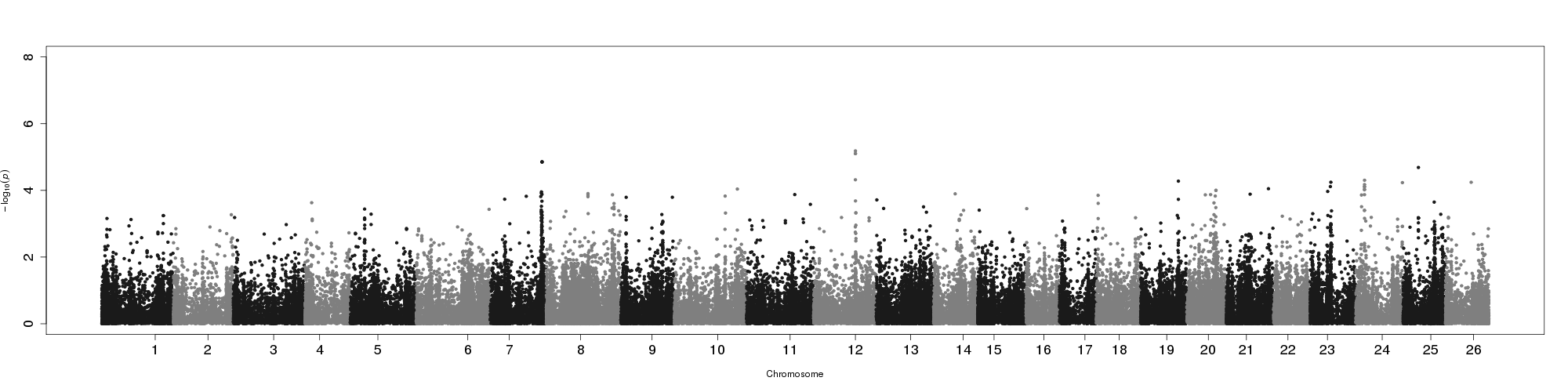

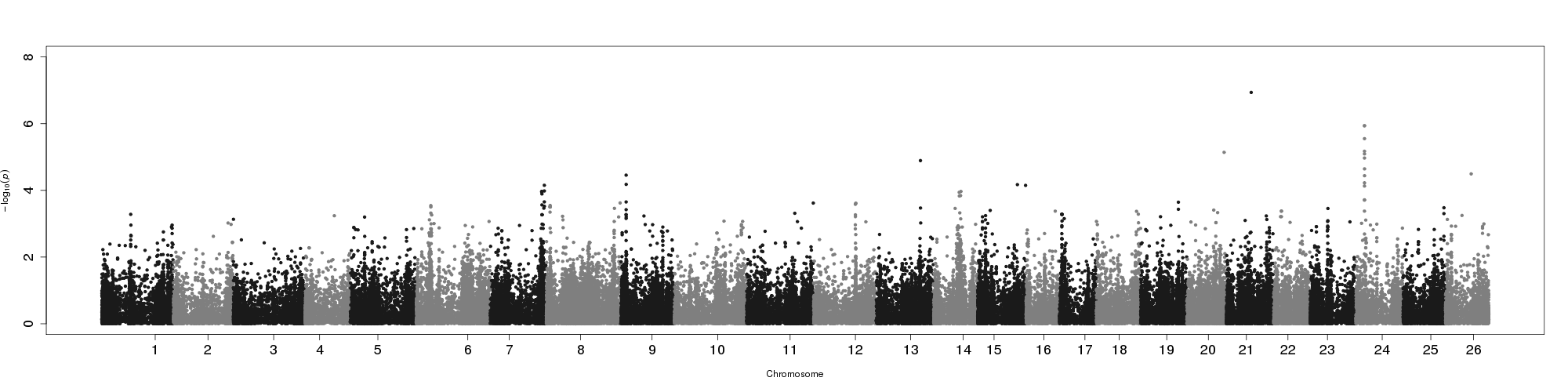

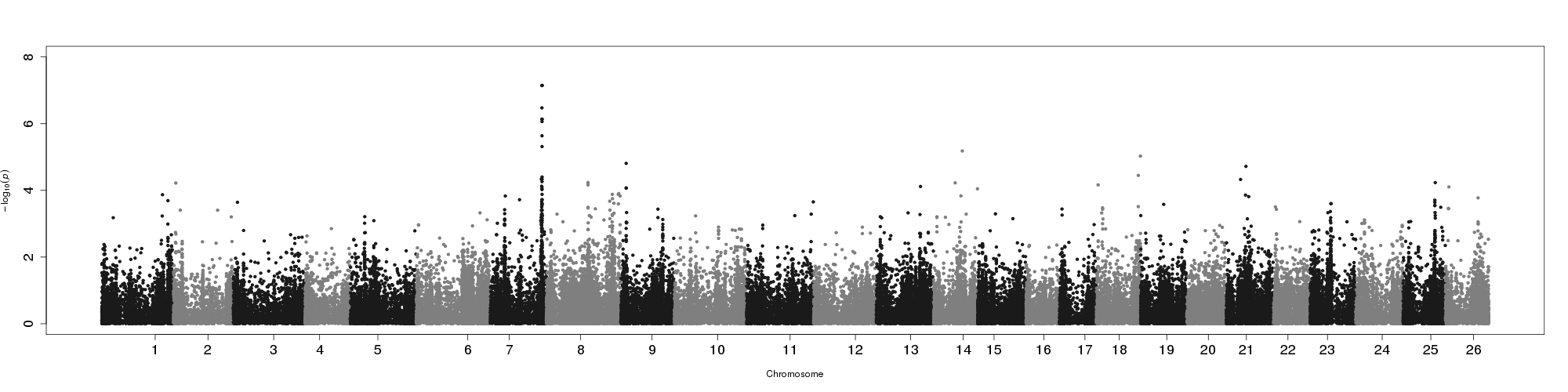

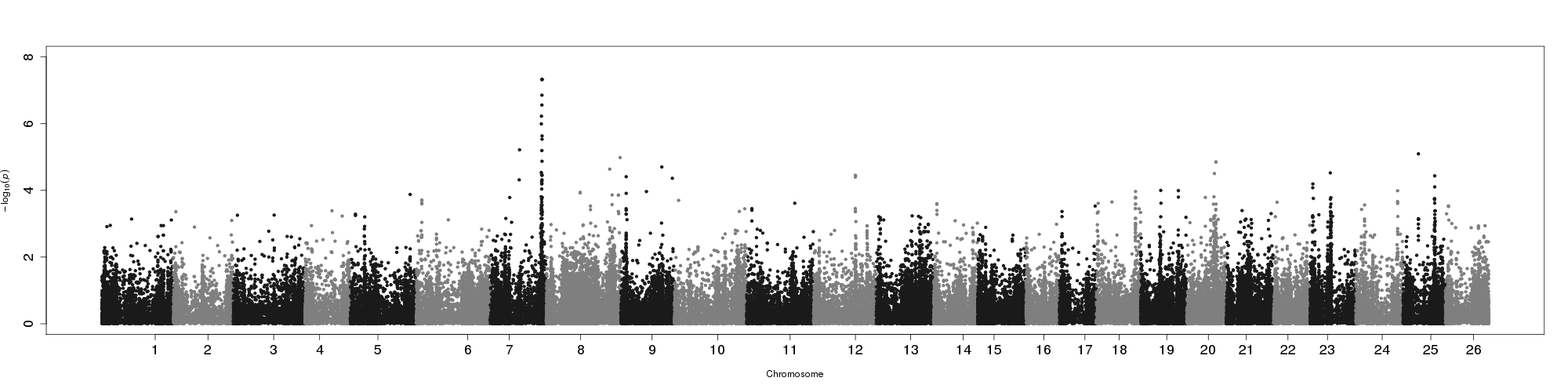

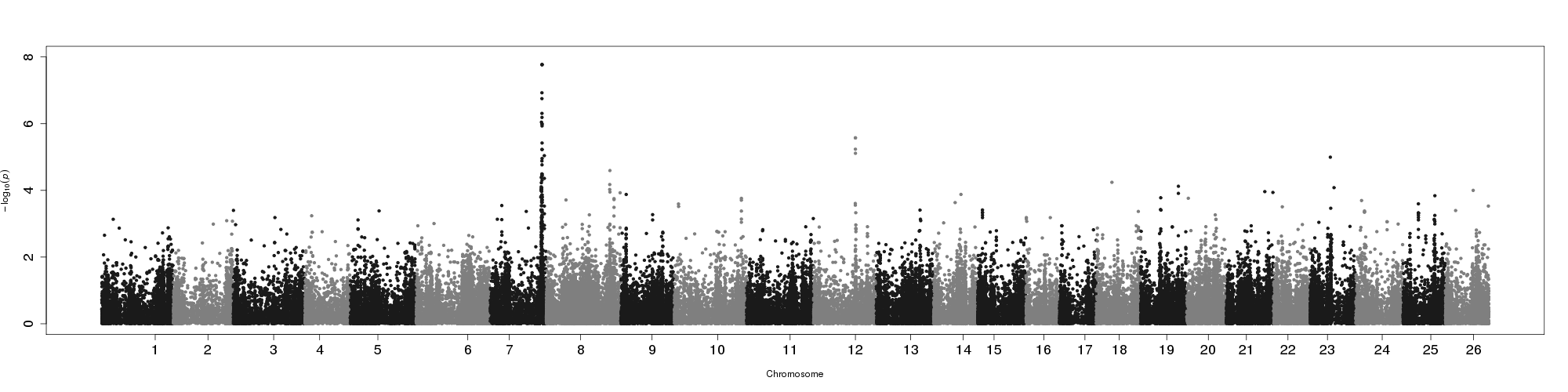

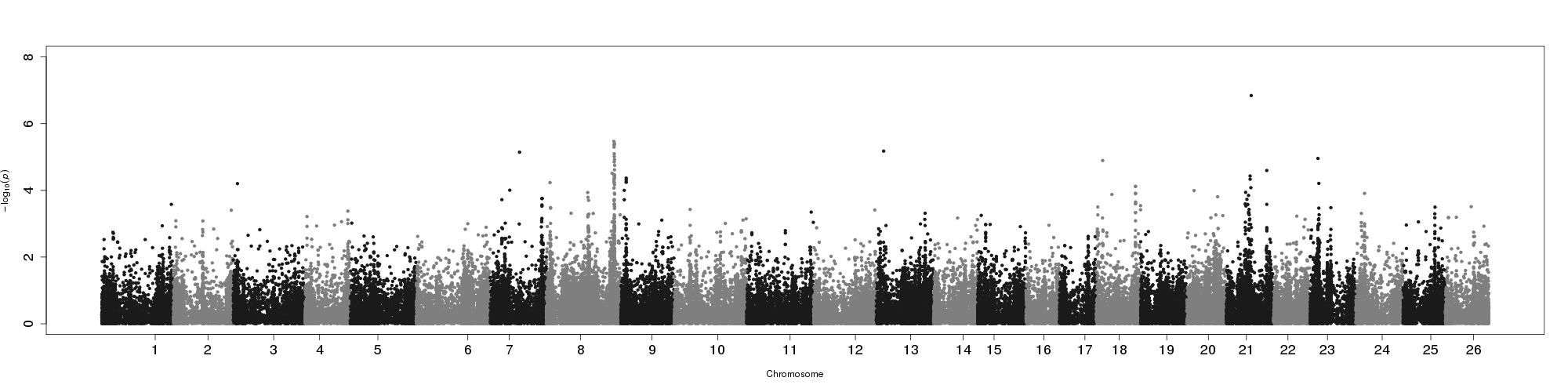

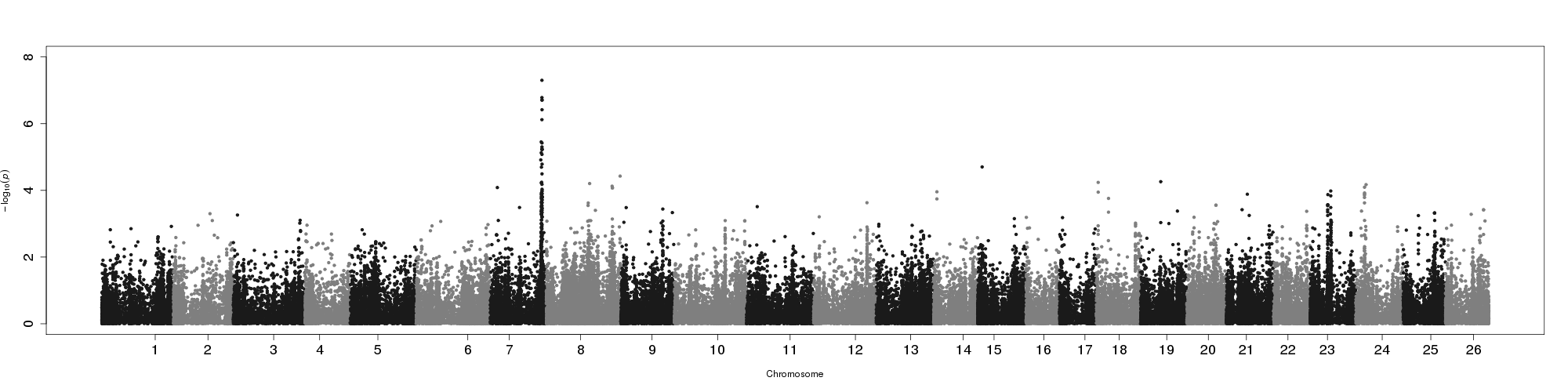

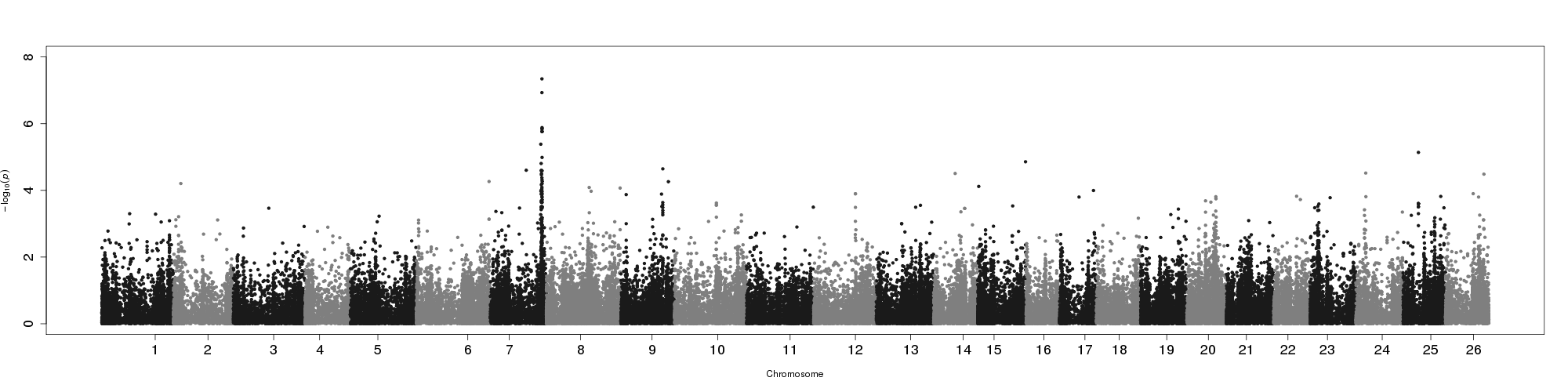

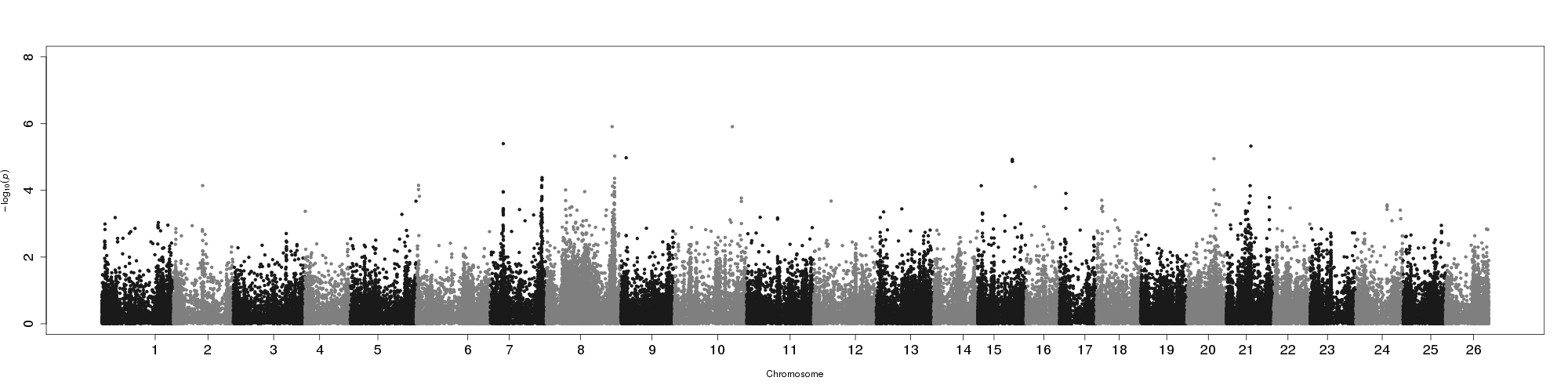
 Fig. S6: The Manhattan block of FS under nine environments.
